# Identifying the Genes Regulated by AtWRKY6 Using Comparative Transcript and Proteomic Analysis under Phosphorus Deficiency

**DOI:** 10.3390/ijms18051046

**Published:** 2017-05-12

**Authors:** Li-Qin Li, Lu-Ping Huang, Gang Pan, Lun Liu, Xi-Yao Wang, Li-Ming Lu

**Affiliations:** College of Agronomy, Sichuan Agricultural University, Chengdu 611130, China; liliqin88@163.com (L.-Q.L.); 18280472482@163.com (L.-P.H.); cqpangang@outlook.com (G.P.); ll2950385@163.com (L.L.); wxyrtl@163.com (X.-Y.W.)

**Keywords:** phosphorus deficiency, AtWRKY6, proteomics analysis

## Abstract

Phosphorus (P) is an important mineral nutrient for plant growth and development. Overexpressing AtWRKY6 (35S:WRKY6-9) was more sensitive and *wrky6* (*wrky6-1*) was more resistant under low Pi conditions. To better understand the function of AtWRKY6 under low phosphate stress conditions, we applied two-dimensional gel electrophoresis (2-DE) to analyse differentially expressed proteins in the shoots and roots between wild type, 35S:WRKY6-9 and *wrky6-1* after phosphorus deficiency treatment for three days. The results showed 88 differentially abundant protein spots, which were identified between the shoots and roots of 35S:WRKY6-9 and *wrky6-1* plants. In addition, 59 differentially expressed proteins were identified in the leaves and roots of 35S:WRKY6-9 plants. After analysis, 9 genes with W-box elements in their promoter sequences were identified in the leaves, while 6 genes with W-box elements in their promoter sequences were identified in the roots. A total of 8 genes were identified as potential target genes according to the quantitative PCR (QPCR) and two dimension difference gel electrophoresis, (2D-DIGE) results, including ATP synthase, gln synthetase, nitrilase, 14-3-3 protein, carbonic anhydrases 2, and tryptophan synthase. These results provide important information concerning the AtWRKY6 regulation network and reveal potential vital target genes of AtWRKY6 under low phosphorus stress. two dimension difference gel electrophoresis, 2D-DIGE

## 1. Introduction

Phosphorus (P) is an indispensable macronutrient involved in many physiological processes, particularly photosynthesis [1]. Although the Pi content in most soils is generally high, Pi availability is extremely low because of its fixed form and slow diffusion [2]. Interestingly, plants have evolved many adaptive strategies to accommodate Pi deficiency. These strategies include morphological, physiological, biochemical, and molecular responses, such as increasing the root/shoot ratio [3]; promoting organic acid synthesis and secretion; and enhancing the expression of acid phosphatases, high-affinity phosphate transporters and transcription factors [4,5,6]. In recent years, some important genes related to low phosphorus have been identified in Arabidopsis. Previous studies have shown that micro RNA399 indirectly regulates PHO2 (E2 conjugase); and PHR1—a MYB (V-myb avian myeloblastosis viral oncogene homolog) transcription factor—binds to the promoters of Pi starvation-induced genes, including micro RNA399 [7]. This finding indicates that PHR1 and micro RNA399 play regulatory roles in the phosphorus-signalling pathway. WRKY45 overexpression in Arabidopsis increases Pi content and uptake, while the suppression of WRKY45 through RNA interference decreases Pi content and uptake. These observations suggest that WRKY45 is involved in the response of Arabidopsis to Pi starvation by the direct upregulation of PHT1; one expression [5]. Other studies have suggested that some protein modifications—such as SUMOylation, phosphorylation, dephosphorylation, and epigenetic modifications [8,9,10,11]—play vital roles in the adaptation of plants to phosphorus deficiency.

Proteome analysis using two dimension difference gel electrophoresis (2D-DIGE) is a well-established technique used to identify genes that respond to environmental stress [12]. Important proteins involved in P signalling and regulatory mechanisms have been identified in maize, *Arabidopsis thaliana*, rice and soybean using proteomics studies [13,14,15,16]. The proteins involved in remodelling the composition of lipid membranes and the activity of the glycolysis alternative pathways showed increased functions in response to phosphorus deficiency in Arabidopsis [15]; and alcohol dehydrogenase, malic enzyme and aconitate hydratase contributed to the contrasting adaptation strategy to Pi deficiency in two ecotypes of *Arabidopsis thaliana* [17].

WRKY (WRKYGQK domain) proteins are plant-specific transcription factors characterized by the presence of one or two highly conserved WRKY domains. The WRKY domain is approximately 60 amino acids in length followed by a Cys_2_His_2_ or Cys_2_HisCys zinc finger motif [18]. Both conserved motifs of the WRKY domain are necessary for the binding affinity of the WRKY protein to the consensus *cis*-acting element W-box (C/T)TGAC(C/T) [19]. AtWRKY6 was first reported to influence the senescence- and pathogen defence-associated PR1 promoter activity in senescence- and defence-related processes [20], and this protein could activate the expression of its target gene SIRK, a receptor-like protein kinase during senescence [21]. AtWRKY6 has also been implicated in phosphorus, boron, and arsenic stress and ABA (abscisic acid) signalling [22,23,24,25]. Overexpressing WRKY6 increases sensitivity under low Pi conditions, and WRKY6 negatively regulates *PHO1* expression by binding to two W-box elements on the *PHO1* promoter. The repression of *PHO1* expression by WRKY6 is released under low Pi conditions, likely reflecting WRKY6 degradation by 26S proteasomes [22].

In the present study, we analysed the differential protein expression profiles of leaves and roots using overexpressing AtWRKY6 lines (35S:WRKY6-9) and knockout mutant (*wrky6-1*) plant materials to identify proteins that are differentially expressed under low Pi conditions. A total of six genes were identified as potential target genes regulated by WRKY6 according to the results of the QPCR (quantitative PCR) and difference in gel electrophoresis (DIGE) experiment. The present study provides valuable information that will lay the foundation for further studies of the functions of genes that respond to phosphorus deficiency.

## 2. Results

### 2.1. Measurements of Biochemical Data in Three Plant Samples

**Figure 1:**
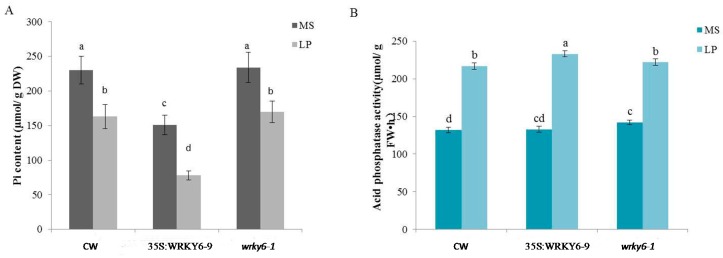
Measurements of Pi content and acid phosphatase activity in three plant samples. Note: (**A**) measurements of Pi content; (**B**) measurements of acid phosphatase activity. Values followed by different lowercase letters mean that data was significantly different among samples in the same treatment at 0.05 level. Bars represent the mean ± SE (*n* = 5).

Seedlings of wild type, 35S:WRKY6-9 and *wrky6-1* were collected after phosphorus deficiency treatment for three days to clarify their physiological status. Firstly, Pi content of seedings were checked. The results demonstrated that under either Pi-sufficient or Pi-deficient conditions, 35S:WRKY6-9 showed similar reduced Pi contents as compared to wild type and *wrky6-1*; there was no difference in wild type and *wrky6-1* under either Pi-sufficient or Pi-deficient conditions (Figure 1A). Acid phosphatase activities in the root of 35S:WRKY6-9 was almost the same as the two samples when growing on MS medium (Pi-sufficient medium); but when growing on LP medium (Pi-deficient medium), phosphatase activities in 35S:WRKY6-9 roots were higher than other materials (Figure 1B). The two results suggested that 35S:WRKY6-9 was more sensitive to low phosphorus stress than wild type and *wrky6-1.* Next, chlorophyll, reducing sugar and sucrose content were also tested to study response difference after phosphorus deficiency treatment in three samples. Chlorophyll content in 35S:WRKY6-9 shoots was higher than wild type under either Pi-sufficient or Pi-deficient conditions. Reducing sugar in 35S:WRKY6-9 shoots was higher than wild type and *wrky6-1* under Pi-sufficient conditions, but there were no differences in the three materials under Pi-deficient conditions. The trend of sucrose content was the opposite; under Pi-sufficient conditions, there was no difference in the three materials, but sucrose content in the 35S:WRKY6-9 shoots was substantially higher than wild type and *wrky6-1* (Table 1). Thus, from these five results we concluded that many physiological changes were produced in 35S:WRKY6-9 in adapting to low Pi stress after three days of treatment. 

### 2.2. 2-D DIGE Determination and MS (Mass spectrometry) Identification of Phosphorus Responsive Proteins in 35S:WRKY6-9 and wrky6-1

**Figure 2:**
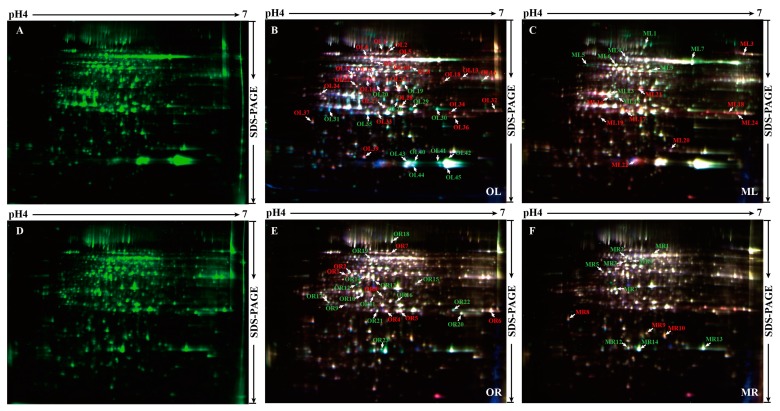
Difference in gel electrophoresis (DIGE) analysis of the differentially abundant proteins between 35S:WRKY6-9 and *wrky6-1.* (**A**) Protein spots were listed in wild type leaves; (**B**) 37 protein spots were listed in 35S:WRKY6-9 leaves; (**C**) 17 protein spots were listed in *wrky6-1* leaves; (**D**) Protein spots were listed in wild type roots; (**E**) 22 protein spots were listed in 35S:WRKY6-9 roots; (**F**) 12 protein spots were listed in *wrky6-1* roots. Red number indicates increase; green number indicates decrease.

The 2-D DIGE experiments were conducted to determine the differentially abundant proteins in the leaves and roots under low phosphorus treatment for 3 days in 35S:WRKY6-9 and *wrky6-1*. We used pH 4–7 IPG (immobilized pH gradient) strips for IEF (isoelectric focusing), and different proteins from three different samples (wild type, 35S:WRKY6-9, and *wrky6-1*) were labelled with different fluorescent dyes. The typical DIGE gels for each low phosphorus treatment and the combined images are presented. A total of 54 protein spots demonstrated a more than 1.5-fold change with statistically significant differences (*p* < 0.05) in 35S:WRKY6-9 and *wrky6-1* leaves. A total of 34 protein spots demonstrated more than a 1.2-fold change with statistically significant differences (*p* < 0.05) in 35S:WRKY6-9 and *wrky6-1* roots (Figure 2). Then, 88 differentially abundant proteins were positively identified using MALDI TOF/TOF MS/MS (Matrix-assisted laser desorption/ionization time of flight mass spectrometry). Every different abundance protein spots were listed in Figures S1 and S2, Mass spectrometry data was collected in Supplementary File S1.

### 2.3. Functional Analysis of the Phosphorus Responsive Proteins

**Figure 3:**
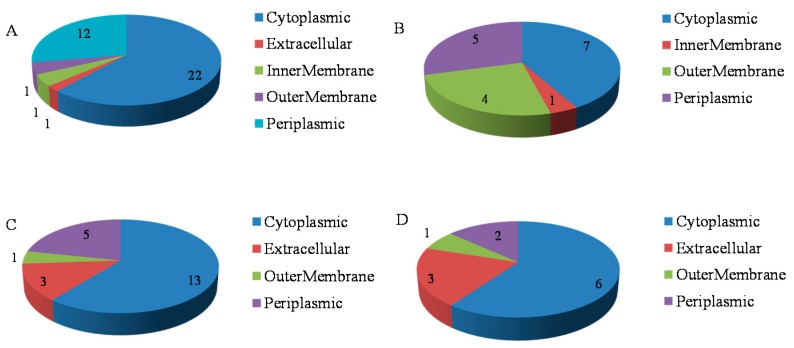
Localization analysis of the 88 differentially abundant proteins identified in 35S:WRKY6-9 and *wrky6-1*. (**A**) 37 protein localizations were shown in shoots of 35S:WRKY6-9; (**B**) 17 protein localizations were shown in shoots of *wrky6-1*; (**C**) 22 protein localizations were shown in roots of 35S:WRKY6-9; (**D**) 12 protein localizations were shown in roots of *wrky6-1*.

The 88 proteins identified had their subcellular locations predicted by TargetP1.1. In 35S:WRKY6-9 shoots, 22 proteins showed cytoplasmic localization, 12 proteins showed periplasmic localization, 1 showed outer membrane and 1 showed inner membrane localization, 1 protein was extracellular (Figure 3A). In *wrky6-1* shoots, 7 proteins showed cytoplasmic localization, 5 proteins showed periplasmic localization, 4 showed outer membrane and 1 showed inner membrane localization, 1 protein was extracellular (Figure 3B).

13 proteins showed cytoplasmic localization, 5 proteins showed periplasmic localization, 3 proteins were extracellular, 1 showed outer membrane in 35S:WRKY6-9 roots (Figure 3C) and 6 proteins showed cytoplasmic localization, 3 proteins were extracellular, 2 proteins showed periplasmic localization, and 1 showed outer membrane in *wrky6-1* roots (Figure 3D). 

**Figure 4:**
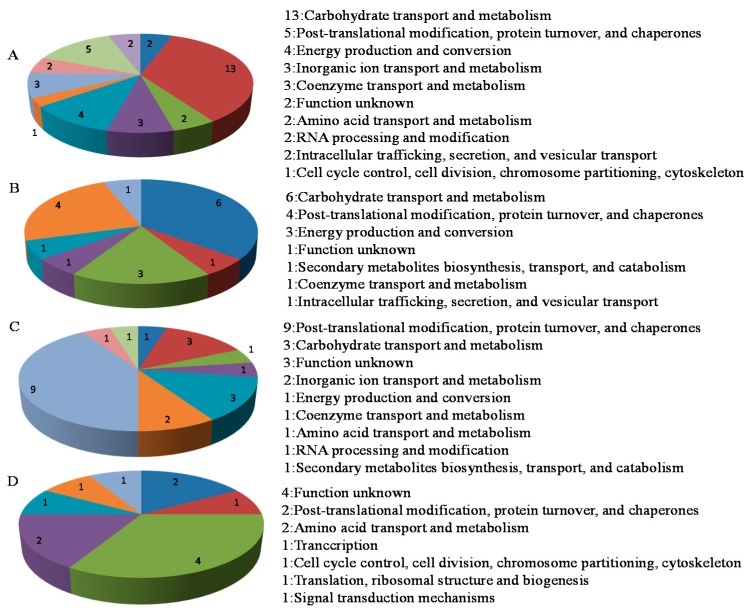
Functional analysis of the 88 differentially abundant proteins identified in35S:WRKY6-9 and *wrky6-1*. (**A**) Function classification of 37 proteins were shown in shoots of 35S:WRKY6-9; (**B**) Function classification of 17 proteins were shown in shoots of *wrky6-1*; (**C**) Function classification of 22 proteins were shown in roots of 35S:WRKY6-9; (**D**) Function classification of 12 proteins were shown in roots of *wrky6-1*.

The 88 proteins were analysed using the EggNOG (evolutionary genealogy of genes: Non-supervised Orthologous Groups) classification system based on their main functions. In 35S:WRKY6-9 shoots, most proteins were related to carbohydrate transport and metabolism, followed by post-translational modification, protein turnover and chaperones, energy production and conversion with protein numbers of 13, 5, and 4 (Figure 4A). In *wrky6-1* shoots, 6 proteins belong to carbohydrate transport and metabolism; 4 belong to post-translational modification, protein turnover and chaperones; and 3 belong to energy production and conversion (Figure 4B). In 35S:WRKY6-9 roots, post-translational modification, protein turnover and chaperones, carbohydrate transport and metabolism, and function unknown were the most common groups; the numbers were 9, 4 and 3 (Figure 4C). In *wrky6-1* roots, 4 proteins belong to function unknown, 3 belong to post-translational modification, protein turnover and chaperones, and 2 belong to amino acid transport and metabolism (Figure 4D).

### 2.4. Analysis of the Differentially Abundant Proteins in wrky6-1

**Figure 5:**
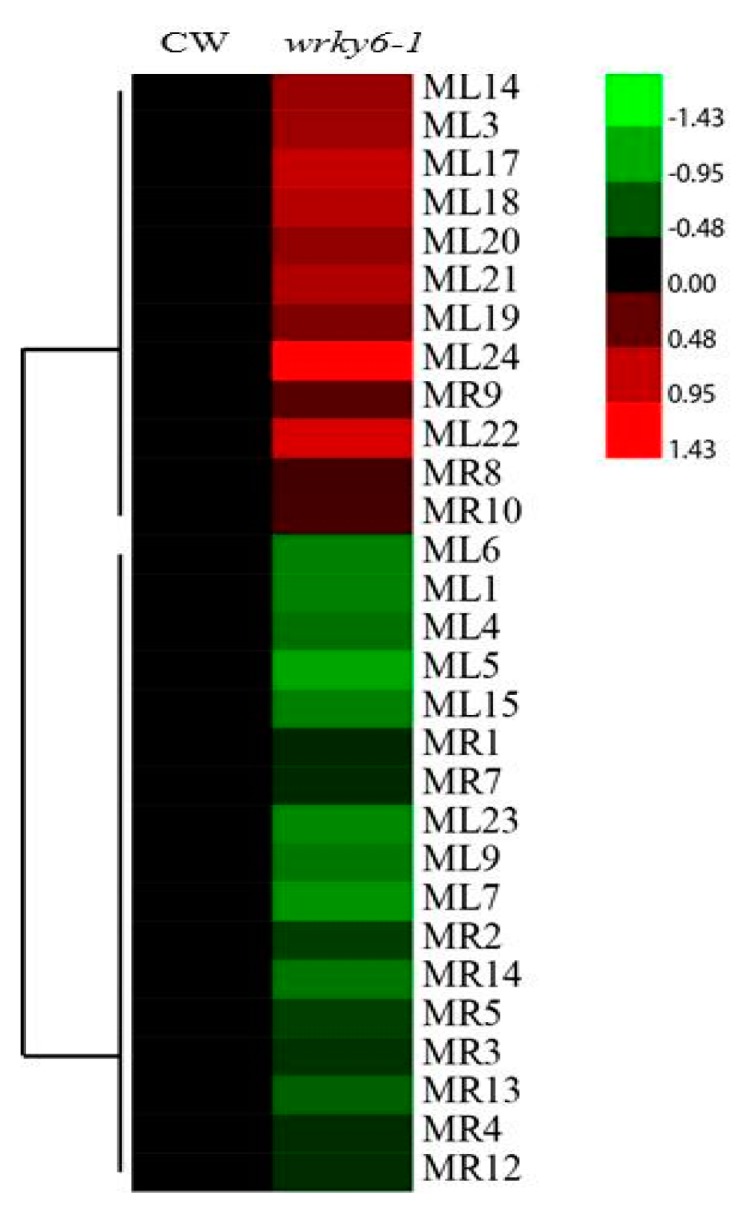
Cluster analysis of differentially abundant proteins in *wrky6-1.* Red colour indicates increase; green colour indicates decrease.

A total of 17 and 12 differentially abundant proteins in leaves and roots were identified between *wrky6-1* and wild type in Table S1. Cluster analysis of differentially abundant proteins in *wrky6-1* were listed (Figure 5). Nine proteins increased in abundance in *wrky6-1* leaves. Three proteins increased, including glutathione *S*-transferase, Ribulose bisphosphate carboxylase, and 2-Cys peroxiredoxin, showing a 2.99-, 2.03-, and 1.89-fold increase, respectively. Three proteins predominantly decreased, including fructose-bisphosphatase, ribulose bisphosphate carboxylase, and myrosinase 1, showing a −1.93, −1.8, and −1.67-fold decrease, respectively. Four proteins increased in abundance in *wrky6-1* roots. Three proteins predominantly increased, including 40S ribosomal protein, and two translationally-controlled tumour proteins, showing a 1.32-, 1.25-, and 1.23-fold increase, respectively. Three proteins, including MLP (myristoylated alanine-rich C kinase substrate like protein)-like protein 328, PYL5 (polyketide cyclase/dehydrase and lipid transport) and glutamine synthetase, predominantly decreased in abundance among most proteins identified, showing a −1.61-, −1.48-, and −1.31-fold decrease, respectively. In leaves, two protein spots identified as 2-Cys peroxiredoxin increased in abundance; three ribulose bisphosphate carboxylase decreased in abundance and one increased in abundance; in roots, two protein spots identified as the same JA (Jasmonates)-responsive protein 1 were decreased, and two translationally-controlled tumour proteins also increased in abundance. 

### 2.5. Analysis of the Differentially Abundant Proteins in 35S:WRKY6-9

**Figure 6:**
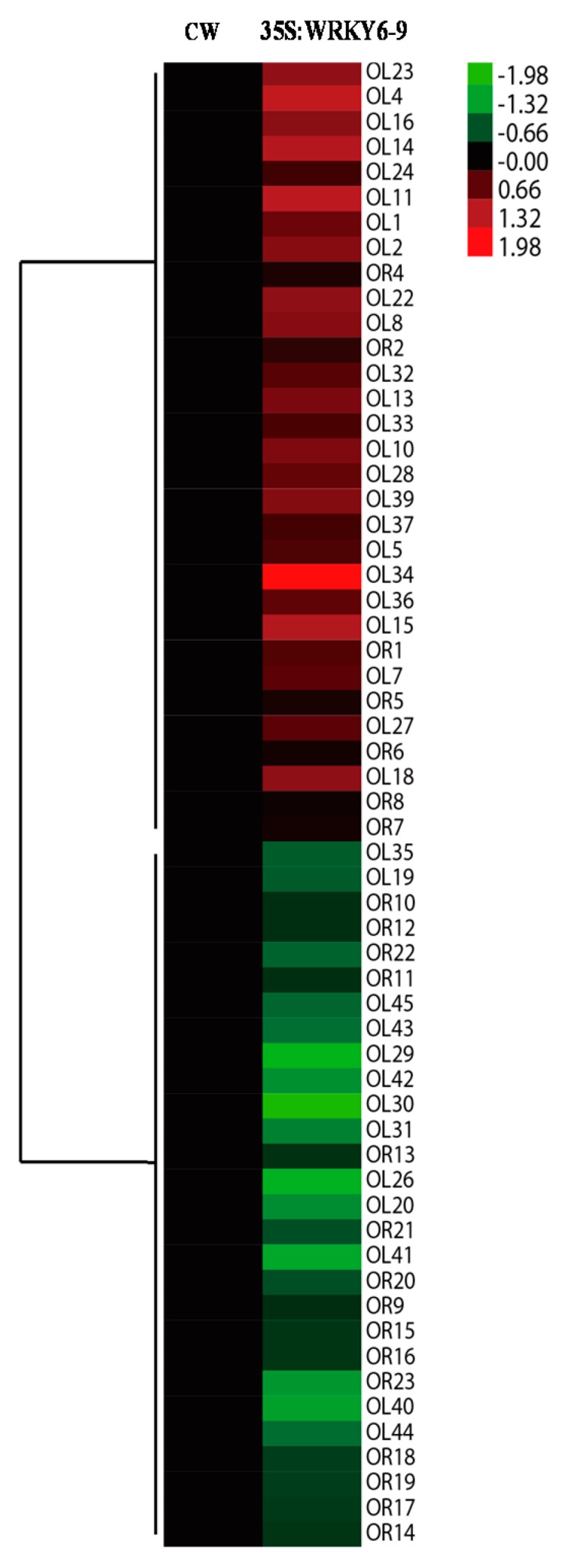
Cluster analysis of differentially abundant proteins in 35S:WRKY6-9. Red colour indicates increase; hreen colour indicates decrease.

A total of 37 differentially abundant proteins were identified between 35S:WRKY6-9 and wild type leaves, and 22 differentially expressed proteins were identified between 35S:WRKY6-9 and wild type roots (Table 2). Cluster analysis of differentially abundant proteins in 35S:WRKY6-9 were listed (Figure 6). The results show that 31 proteins increased in abundance in 35S:WRKY6-9 leaves. Three proteins, including glutathione S-transferase, the ATP synthase CF1 alpha subunit, and Gln synthetase predominantly increased, showing a 3.89-, 2.82-, and 2.73-fold increase, respectively. Three proteins predominantly decreased, including two carbonic anhydrase 1 proteins and carbonic anhydrase 2, showing a −4.04-, −2.98-, and −2.69-fold decrease, respectively. Eight proteins increased in abundance in 35S:WRKY6-9 roots. Three proteins increased, including carbohydrate kinase, ACC oxidase, and Chaperonin 10, showing a 1.62-, 1.37-, and 1.27-fold increase, respectively. Three proteins, including glycine-rich RNA-binding protein 8, 2 glutathione S-transferases and ferritin 1 decreased, showing a −2.16-, −1.6-, and −1.4-fold decrease, respectively.

After analysis, one 14-3-3 protein (AT4G09000) increased in abundance in 35S:WRKY6-9 leaves and one (AT5G10450) decreased in abundance in roots. One mercaptopyruvate sulfurtransferase 1 protein increased in abundance in 35S:WRKY6-9 leaves and decreased in abundance in roots. Two glutathione transferases increased in 35S:WRKY6-9 leaves, and two glutathione transferases decreased; glutathione transferases increased in 35S:WRKY6-9 roots. Two protein spots identified as the same ferritin 1 increased in abundance in 35S:WRKY6-9 leaves and decreased in roots.

### 2.6. Potential Target Genes Regulated by WRKY6 in the Leaves and Roots

A total of 59 differentially abundant proteins were identified in the leaves and roots of 35S:WRKY6-9 plants. Further analysis revealed that these proteins corresponded to 59 genes. Firstly, 29 genes in leaves were checked by quantitative RT-PCR between 35S:WRKY6-9, *wrky6-1* and wild type plants, 20 gene expression levels were consistent with DIGE experiment. Nine genes were not consistent. OL4 was not checked, because it was a chloroplast genome gene. Also, 22 genes in the roots were checked; 18 expression levels were consistent with DIGE experiment; 4 genes were not consistent. These gene expression patterns were listed in Table S2. 

WRKY protein activates or depresses target gene expression by binding to the W-box in the promoter region. To identify target genes of WRKY6, 52 genes in 35S:WRKY6-9 leaves and roots were examined to find W-box elements in their promoter regions (2 kb); the results suggested there were 17 genes with 1–2 W-box elements (Table S3. Glutathione transferase has 2 W-box elements in the roots; 14-3-3 protein has 2 W-box elements. The other gene has one W-box element in the promoter regions.

**Figure 7:**
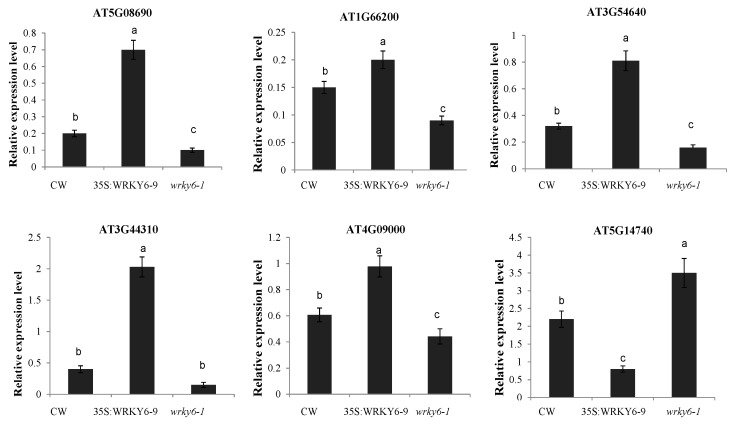
The expression levels of 8 potential target genes are listed. Bars represent the mean ± SE (*n* = 3), the lowercase letters indicate a significant difference between the groups. Notes: AT5G08690: Mitochondrial F1 ATP synthase β subunit; AT1G66200: Gln synthetase; AT3G44310: Nitrilase I; AT4G09000: 14-3-3 protein; AT5G14740: Carbonic anhydrase 2; AT3G54640: Tryptophan synthase.

**Figure 8:**
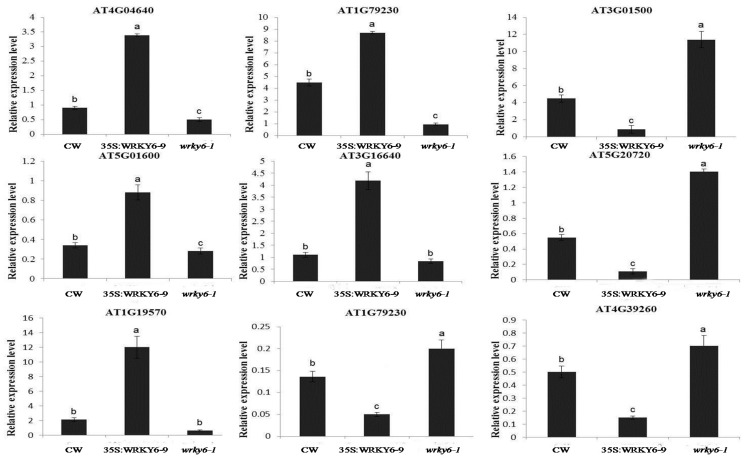
The expression levels of 9 indirect regulated genes are listed. Bars represent the mean ± SE (*n* = 3), the lowercase letters indicate a significant difference between the groups.Notes: AT4G04640: ATP synthase gamma; AT1G79230: Mercaptopyruvate sulfurtransferase 1; AT3G01500: Carbonic anhydrase 1; AT5G01600: Ferritin 1; AT3G16640: Translationally-controlled tumour protein-like protein; AT5G20720: Chaperonin 10; AT1G19570: Dehydroascorbate reductase; AT4G39260: Glycine-rich RNA-binding protein 8.

According to gene expression patterns and W-box element numbers, we found 6 genes as potential target genes because they showed contrasting expression levels in 35S:WRKY6-9 and *wrky6-1*, including mitochondrial F1 ATP synthase, gln synthetase, nitrilase, 14-3-3 protein, carbonic anhydrases 2, and tryptophan synthase (Figure 7). Also, 35 genes in leaves and roots without W-box elements on the promoter had their expression level checked among three materials using qRT-PCR to find indirect regulated genes by WRKY (Table S2). The results revealed ATP synthase, mercaptopyruvate sulfurtransferase 1, carbonic anhydrase1, ferretin 1, and translationally-controlled tumour proteins as indirect regulated genes in the leaves. Chaperonin 10, dehydroascorbate reductase, mercaptopyruvate sulfurtransferase 1, and glycine-rich RNA-binding protein 8 were indirect regulated genes in the roots (Figure 8).

## 3. Discussion

### 3.1. Physiological Changes Were Induced after Phosphorus Deprivation

Three plant materials (wild type, 35S:WRKY6-9, and *wrky6-1*) were collected to test physiological changes after phosphorus deprivation for three days. Protein gel blot analysis suggested WRKY6 protein was degraded after low Pi stress for three days [22], so we speculated that many downstream genes of WRKY6 were regulated at transcription level. At the same time, abundance of the encoding proteins of these genes was changed to play a vital role in response to low Pi stress in Arabidopsis. With both growing on MS medium and LP medium, 35S:WRKY6-9 seedings had high chlorophyll content and less Pi content among three plant material. This indicated that the regulatory effect of WKRY6 overexpression on two contents could be independent of the Pi status of the plant. Shoot P concentration was thought to be an important factor in Arabidopsis when overexpressing MYB62. The chlorophyll content was higher and the total Pi content was significantly decreased relative to the wild-type plants under both P+ and P− conditions [26]. This is consistent with 35S:WRKY6-9 plants. Maybe transcriptional regulators as coordinated spatio-temporal regulations had many of the same functions in the regulation of Pi-responsive genes to maintenance of Pi homeostasis in plants [27]. Sugar and sucrose was reduced more in the seedings of the three materials after LP treatment than in the control, because under P starvation, plants accumulate sugars and starch in their leaves to respond to low Pi stress [28]. The sucrose content was higher in 35S:WRKY6-9 shoots than wild type and *wrky6-1* under Pi-deficient conditions. Hermans et al. reported that an increased leaf sucrose concentration also results in more transport proteins delivering sucrose to the phloem, which promoted sucrose transport to roots to increase root shoot ratio under P starvation [29]; the result was consistent with more roots of 35S:WRKY6-9 in either Pi-sufficient or Pi-deficient conditions [22]. 

Increased root sucrose content appear to result in high phosphatases activity [30]; the overexpression of SUC2 (a SUC transporter) had high sucrose concentrations in both shoot and root tissues, and enhanced sensitivity to low P availability, including increased acid phosphatase activity [31]. Acid phosphatase activities in 35S:WRKY6-9 was higher than the other samples after low Pi stress treatment. This was probably due to sucrose concentration differences in roots.

These data indicated that the distribution of the photosynthetic carbon metabolism was altered in 35S:WRKY6-9 under low phosphate conditions.

### 3.2. Proteomics Revealed That the Enzymes Primarily Involved in Photosynthesis Are Differentially Abundant in 35S:WRKY6-9 and wrky6-1

**Figure 9:**
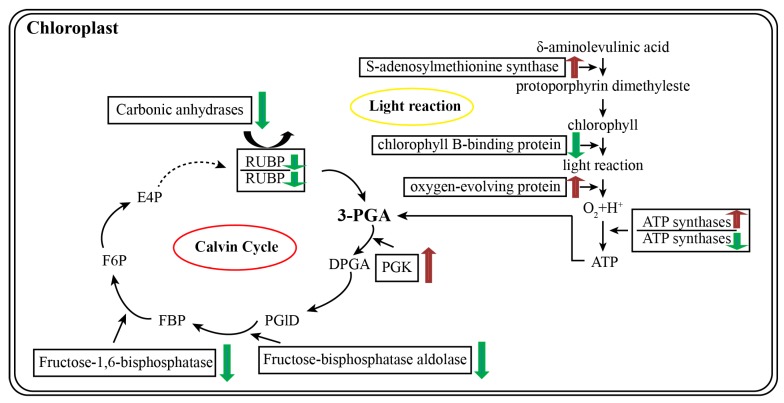
Schematic representation of the differentially abundant proteins involved in photosynthesis in Arabidopsis. Red arrow indicates increase; green arrow indicates decrease; dashed arrow indicates reactions of multiple steps.

Photosynthesis is significantly decreased during phosphorus deprivation [32]. A total of 16 proteins related to photosynthesis have been identified in 35S:WRKY6-9. Among these proteins, 5 proteins increased in abundance and 11 proteins decreased in abundance. A total of 7 differentially expressed proteins were identified in *wrky6-1*, and among these proteins, 1 proteins increased in abundance and 6 proteins were decreased (Figure 9). The ATP synthase CF1 α subunit (OL4) increased in abundance in 35S:WRKY6-9 leaves and decreased 1.57-fold in *wrky6-1* compared to wild-type plants. ATP synthase synthesizes ATP to power the dark reaction of photosynthesis, and ATP levels in the leaves decreased 29.32% under phosphorus deficiency stress. Decreased ATP synthase activity reduced carbon assimilation in maize [33,34], in addition to the function of this protein of Arabidopsis in mitochondrial oxidative phosphorylation, and also as a key negative regulator of plant cell death [35]. However, its expression levels were opposite in 35S:WRKY6-9 and *wrky6-1* leaves. The ATP synthase CF1 α subunit e is a member of the chloroplast genome and has no promoter; thus, this protein is likely an indirect target gene of WRKY6. However, which gene can directly regulate ATP synthase expression? Answering this question requires additional studies.

A carbonic anhydrase 2 (OL26) and two carbonic anhydrase 1 (OL29, 30) proteins decreased 2.98-, 4.04- and 2.69-fold in 35S:WRKY6-9, respectively. Six ribulose bisphosphate carboxylase proteins (OL40, 41, 42, 43, 44, 45) were downregulated; this is possibly related to high sucrose concentration in 35S:WRKY6-9 shoots, as many genes encoding photosystesubunits and small subunits of Rubisco showed decreased expression when there were high sucrose concentrations in Arabidopsis leaves, resulting in a reduction of photosynthesis [36,37]. Physiological analysis showed that phosphorus deficiency reduces the carboxylase activity of RuBisCO 65.02% [38]. AtRBCS1A exhibits light-dependent expression [39]. Decreased RBCS3B expression causes severe photoinhibition, reflecting the accumulation of ROS, which accelerates the photodamage of PSII and inhibits the repair of PSII in RBCS3B-7 (cosuppression material) [38]. Phosphorus starvation reduces the rate of RuBP regeneration and inhibits the Calvin cycle. Among the six proteins related to the Calvin cycle, five proteins decreased and phosphoglycerate kinase (PGK) (OL10) increased 1.99-fold in 35S:WRKY6-9 leaves; currently, there are no reports concerning this gene as being involved in low Pi stress. PGK (phosphoglycerate kinase) is involved in ATP production and demonstrated as stress responsive in sunflowers [40], rice [41] and soybeans [42]. PGK has also been identified in responses against abiotic stresses in crops using organ-specific proteome analyses [43]. Overexpression of OsPgk2a-P in transgenic tobacco plants increased salt tolerance [44]. 

*S*-adenosylmethionine synthase (OL8) increased; this protein was also accumulated during phosphorus starvation in maize [34]. These data suggest that this enzyme was involved in the responses of Arabidopsis leaves to phosphorus starvation. Ferredoxin (OL35)—which is involved in photosynthetic electron transport—was downregulated 1.55-fold in 35S:WRKY6-9 leaves, and this protein was also downregulated in abundance in maize under phosphorus deprivation, which caused vital effects on NADPH electron transfer [34]. Fructose-bisphosphate aldolase (OL19, OL20) was decreased 1.53-fold and 2.02-fold, suggesting that sucrose synthesis was reduced in 35S:WRKY6-9 leaves; perhaps sucrose degradation was not decreased, so sucrose content was higher in 35S:WRKY6-9 shoots than wild type and *wrky6-1* under phosphorus deprivation. The 33 kDa oxygen-evolving protein (ML23) increased, and this protein shows increased expression in rice infected with the rice stripe virus [45].

### 3.3. Proteins Related to Energy Metabolism and Stress Were Identified in 35S:WRKY6-9

In the current study, proteins related to energy metabolism and stress were differently expressed in 35S:WRKY6-9 using proteomics analysis. The enzyme 2,3-bisphosphoglycerate-independent phosphoglycerate mutase is key in glucose metabolism during the conversion between 3- and 2- acid-catalysed ADP, and two phosphoglycerate mutases (OL1 and OL2) increased 1.82- and 2.07-fold in the leaves. Malate dehydrogenase (OL13) increased 1.86-fold in the leaves, and the overexpression of the rice malic enzyme gene in Arabidopsis conferred tolerance to salt, osmotic and drought stresses [46]. Two mercaptopyruvate sulfurtransferase proteins (MST) (OL22, OR12) increased and decreased in abundance in 35S:WRKY6-9 leaves and roots. Their expression patterns were confirmed by qRT-PCR analysis. The different expression pattern in the leaves and roots suggests that these proteins have different functions in plants. Previous studies have suggested that MST activity is higher in older rather than in younger Arabidopsis plants [47], and this protein functions in Arabidopsis senescence and cyanide detoxification [48], consistent with the research paper suggesting that 35S:WRKY6-9 leaves exhibit early senescence symptoms [21].

Triose phosphate isomerase plays an important role in glycolysis, is essential for efficient energy production, and primarily functions in the catalytic conversion of triose phosphate isomers between dihydroxyacetone phosphate. Cytosolic triose phosphate isomerase (OL27 and OL28) increased 1.68- and 1.77-fold in the leaves. A triose phosphate isomerase from rice (OscTPIn) was induced in response to various abiotic stresses and methylglyoxal [49]. Several enzymes involved in oxidative stress have been described in various stress proteomics studies [50]. In the leaves, two glutathione peroxidases (OL32, OL34) increased 1.65- and 3.85-fold; these enzymes can reduce H_2_O_2_ to H_2_O to protect plants from oxidative damage under abiotic stress [51]. One SOD (Superoxide dismutase) (OL36) increased 1.71-fold in response to phosphorus deficiency. Furthermore, SOD increased approximately three-fold in a phosphorus-tolerant rice variety but increased 30% in phosphorus-sensitive rice [52]. 

Three ferredoxin protein (OL33, OL35, OR21) spots decreased. Ferritin 1 (*AtFer1*) regulates the phosphate starvation response 1 (AtPHR1) transcription factor and plays a vital role in iron and phosphate homeostasis under phosphate starvation stress [53]. A tCTP (translationally-controlled tumour protein from *Arabidopsis thaliana*) (OL37) increased 1.52-fold; in tobacco, NtTCTP (translationally-controlled tumour protein from *Nicotiana tabacum*) interacts with NtHK1 (histidine kinase 1 [ethylene receptor]), which stabilizes NtHK1 protein and reduces the sensitivity of plants to ethylene to promote cell proliferation and avoid excess ethylene in normal plant growth and development inhibition [54]. Therefore, we speculated that the leaves of 35S:WRKY6-9 plants grow bigger on MS medium because of high AtCTP expression levels. MLP-like protein 423 (OL 39) was upregulated 2.04-fold in 35S:WRKY6-9, and its function as a positive regulator during ABA (abscisic acid) responses confers drought tolerance in *Arabidopsis thaliana* [55].

Proteins such as A pfkB-like carbohydrate kinase (OR1) increased 1.62-fold, and two studies have suggested that this induction occurred in response to salt and drought in maize and sunflower in proteomics analysis [56,57]. Aminocyclopropane-1-carboxylic acid (ACC) oxidase (OR2) increased 1.37-fold in the roots. ACC oxidase is a key enzyme in the ethylene biosynthesis pathway. The ACC synthesis inhibitor AVG from *Arabidopsis thaliana* inhibits the occurrence of adventitious roots, and the root morphology was changed [58,59]. Ethylene regulates root hair growth and root elongation development [60]; thus, the high expression of ACC oxidase could contribute to root morphology changes in 35S:WRKY6-9 lines.

The expression of the chaperonin 10 gene (OR4) was not consistent between the results of the QPCR and DIGE experiment, as this protein increased in 35S:WRKY6-9 according to the DIGE experiment, but decreased in 35S:WRKY6-9 and increased in *wrky6-1* according to the QPCR experiment. Thus, we speculated that WRKY6 negatively regulates chaperonin 10 expression under low phosphorus. This gene is reportedly induced under heat-shock abiotic stress [61]. Dehydroascorbate reductase (OR5) was upregulated 1.25-fold. In tobacco, this protein was reported to affect the level of foliar ROS and photosynthetic activity during leaf development; at least, the protein influences the rate of plant growth and leaf aging [62]. Thus, high expression levels of the protein may be related to the leaf senescence symptoms of 35S:WRKY6-9. Glutathione peroxidase (OR6) increased 1.23-fold, and 3 glutathione peroxidases (OR16, 20, 22) decreased 1.3-, 1.4- and 1.6-fold in the present study; these proteins were important and participated in a variety of abiotic stresses [63,64]. A mitochondrial chaperonin (HSP60) (OR7) was upregulated, and previous studies have suggested that this protein can combine with the F-ATPase alpha subunit in maize. HSP60 is not only involved in the nuclear gene encoding the protein into mitochondria and protein folding and assembly [65], but perhaps it is also vital to the synthesis of new proteins under low phosphorus. Glycine-rich RNA-binding protein 8 (OR23) decreased in abundance; this gene is downregulated after treatment with the stress-associated hormone, abscisic acid (ABA) [66], and rapidly increased in response to peroxide-induced oxidative stress [67], suggesting that this protein plays a vital role in stress.

### 3.4. Potential Target Genes of WRKY6 Identified Using Proteomics and qRT-PCR Analysis

A total of 6 genes were identified as potential target genes using DIGE and qRT-PCR analysis. First, a mitochondrial F1 ATP synthase showed increased abundance in 35S:WRKY6-9 leaves and decreased in *wrky6-1* compared to wild type. In addition, one W-box element was identified in the promoter of this gene. Thus, WRKY6 may activate this gene expression to adopt to low phosphorus stress. Overexpression of a mitochondrial ATP synthase (AtMtATP6) confers tolerance to several abiotic stresses in *Saccharomyces cerevisiae* and *Arabidopsis thaliana* [68]. 

In leaves, glu synthetase (OL11) showed an increased expression in 35S:WRKY6-9 and decreased expression in *wrky6-1*; in roots two protein spots (MR5 and MR6) corresponding to the same gene decreased in wrky6-1 by DIGE analysis. Thus, this gene expression pattern was consistent in two experiments. In rice, GS1 2-overexpressed lines exhibited higher sensitivity to salt, drought, and cold stress [69]; this phenotypic is similar to 35S:WRKY6-9 and sensitive to abiotic stress. Nitrilase (OL18) showed the opposite expression pattern in 35S:WRKY6-9 and *wrky6-1* leaves. Nitrilase was involved in the IAA biosynthesis pathway and the defence response to microbial pathogens [70]. The 14-3-3 proteins (OL24, OR17) were upregulated and downregulated. As a 14-3-3 family member, *GRF9* plays a role in Pi starvation-induced responses [71]. Zhou et al. reported that the interaction between 14-3-3 proteins and SOS2 activates the SOS pathway for salt tolerance [72]. Carbonic anhydrase 2 (CA2,OL26) proteins decreased 2.98- fold in 35S:WRKY6-9. CA (Carbonic anhydrase) accelerates a conversion of carbon dioxide and water to bicarbonate and protons in the cell and CO_2_ transport to active photosynthetic cells, thereby improving the photosynthesis rate [73]. Under low phosphorus stress, 35S:WRKY6-9 leaves exhibit yellowing, and senescence was likely affected by decreased carbonic anhydrase expression. A tryptophan synthase (OR8) increased in abundance; in response to low Pi starvation this protein can promote the formation of tryptophan, the auxin precursor, for auxin-induced lateral root formation in the siz1 mutant in response to low Pi starvation [8]; this agrees with more lateral root formation in 35S:WRKY6-9 under low phosphorus stress.

## 4. Materials and Methods

### 4.1. Plant Materials and Low Phosphorus Treatment

The WRKY6 overexpression line (35S:WRKY6-9) and the WRKY6 knockout mutant (*wrky6-1*) were kindly provided by ImreE. Somssich (Max-Planck-Institute, Munchen, Germany). The *Arabidopsis thaliana* seeds (wild type, 35S:WRKY6-9, and *wrky6-1*) were surface sterilized using mixed solutions of NaClO (0.5%) and Triton X-100 (0.01%) for 15 min, followed by washing five times with sterilised distilled water on a clean working table. The sterilised seeds were first incubated in Petri dishes containing MS agar (0.8%) medium (containing 1.25 mM KH_2_PO_4_ and 3% sucrose) at 4 °C for 2 days prior to germination. The seeds were germinated at 22 °C under constant illumination at 40 µmol·m^−2^·s^−1^ and the 7-day-old seedlings were transferred to Pi-deficient medium (LP) for 3 days. The LP medium was generated by the modification of MS medium, such that the Pi (supplied with KH_2_PO_4_) concentration in LP medium was 10 µM, and agar was replaced with agarose (Promega Corporaion, Madison, WI, USA) to avoid the contamination of phosphorous. After 3 days of LP treatment, three plant materials were collected for measurements of biochemical data such as Pi content, reducing sugar content, sucrose content, chlorophyll content in seedlings, and acid phosphatase activity in the root. For Pi content measurements, the seedings were oven dried at 80 °C for 48 h and ashed in a muffle furnace at 300 °C for 1 h and 575 °C for an additional 5 h and then dissolved in 0.1 N HCl. The total Pi content in the samples was quantified as described previously [74]. Extraction and content determination of the chlorophyll experiment were performed according to the method [75]. Measurements of reducing sugar and sucrose content were carried out following the methods described [76,77]. Approximately 0.5 g of root tissue was collected to check acid phosphatase activity using p-nitrophenol phosphate as the substrate. Activity was quantified by comparing the absorption at 410 nm to a standard curve of diluted p-nitrophenol solutions and NaOH [78]. At the same time, the leaves and roots of three plant materials were collected for the extraction of total protein and RNA.

### 4.2. Protein Extraction and 2-D DIGE Analysis

Leaf and root proteins of six Arabidopsis samples were extracted using the Borax/PVPP (Polyvinyl polypyrrolidone)/Phenol (BPP) protocol [79]. The protein concentration was determined using a Bradford assay. Bovine serum albumin (BSA) was used as a standard. DIGE (two-dimensional difference in gel electrophoresis) was performed as previously described [80], and the details of 2D-DIGE experiments were shown in Table S4; wild type was used as a control. Each protein sample extracted from the leaves and roots was treated with Pi-deficient medium and labelled with a ratio of 250 pmol Cy2, Cy3 and Cy5 minimal labelling dye (GE Healthcare, Little Chalfont, UK) per 50 μg of protein. The reactions were quenched after the addition of 1 μL of 10 mM lysine, followed by mixing and incubation on ice for 10 min in the dark. The three labelled and quenched samples were combined, and a total of 150 μg of protein was added to rehydration buffer (7 M urea, 2 M thiourea, and 2% *w*/*v* CHAPS) containing 0.5% IPG buffer to a final volume of 450 μL. IEF was performed on pH 4–7, 24 cm IPG strips (GE Healthcare). Two-dimensional electrophoresis was performed using 12.5% SDS-PAGE (dodecyl sulfate sodium salt polyacrylamide gel electrophoresis). The Cy2-, Cy3- and Cy5-labelled 2-DE images were subsequently acquired using a Typhoon Trio scanner (GE Healthcare), and the DIGE images were analysed using DeCyder 7.0 software (GE Healthcare). A differential in-gel analysis module was used for spot detection, and a biological variation analysis module was applied to the three biological repeats to identify the differentially abundant spots with higher than 95% confidence in 35S:WRKY6-9 and *wrky6-1* plant samples. 

### 4.3. Protein Identification via Mass Spectrometry and Bioinformatics Analysis

The target protein spots were manually excised and in-gel digested using bovine trypsin as previously described [80]. The mass spectra of the peptides were acquired on an AB 5800 MALDI-TOF/TOF mass spectrometry (MS) instrument (AB SCIEX, Foster City, CA, USA) equipped with a neodymium laser with a laser wavelength of 349 nm. The measured tryptic peptide masses were transferred to Protein Pilot Software (AB SCIEX, Foster City, CA, USA) and used for a Mascot Algorithm (version2.2, Matrix Science, Boston, USA) search of the taxonomy of Viridiplantae in the nonredundant NCBI (NCBInr) database (NCBIprot 20170414; 119815302 sequences; 43934382767 residues). Proteins with protein score confidence intervals above 95% (total protein score higher than 50) were considered as confident identifications; at the same time, at least two peptides or a coverage of 5% is requested to accept the protein identification. The identified proteins were searched using EggNOG (https://eggnog.embl.de) to confirm their functions, followed by TargetP1.1 (http://www.cbs.dtu.dk/services/TargetP/) to predict their subcellular locations. Subsequently, a gene ontology (GO) analysis was performed using the Blast software 2GO as previously described [81]. Finally, in-house BLAST searching of UniProtKB was performed for each protein to identify its homology and confirm its detailed cellular component, biological process, and molecular function.

### 4.4. Gene Expression Analysis by qRT-PCR

For qRT-PCR analysis, total RNA in leaves and roots after 3 days of LP treatment were isolated using TRIzol reagent (Invitrogen, Carlsbad, CA, USA) according to the manufacturer’s protocol. RNA samples were treated with RNasefree DNase I (Takara, Tokyo, Japan) to digest genomic DNA. The quality of the RNA was checked using an Agilent 2100 RNA Bioanalyzer (Agilent, Santa Clara, CA, USA). Then, the cDNA was synthesized from the treated total RNA by SuperScript II Reverse Transcriptase (Invitrogen) using Random Hexamer Primers (Promega). The qRT-PCR was performed on a 7500 Real Time PCR System machine (Applied Biosystems, Carlsbad, CA, USA) following the manufacturer’s protocols. The 20 μL reaction mixture included 10 μL of 2× SYBR Green Master Mix Reagent (TaKaRa Corporaion, Dalian, China), 10 ng cDNA template, and 0.3 μM each of gene-specific primers. The thermal treatment was 10 min at 95 °C, then 40 cycles of 15 s at 95 °C, 1 min at 60 °C. Amplification was followed by a melt curve analysis. The 2^−ΔΔ*C*t^ method was used for relative quantification. Actin 2/8 expression was used as an internal control. The statistical significance was evaluated by Paired *t*-test analysis. The primers used are listed in Table S5.

### 4.5. Statistical Analysis

The data were subjected to the unpaired student *t* test at levels of *p* ≤ 0.01 and *p* ≤ 0.05. Data are shown as mean ± SE (*n* = 5 or 3) and n represents the biological replicates. Excel 2010 (Microsoft Corporaion, Redmond, WA, USA) and SPSS14.0 software (IBM, New York, NY, USA) were used for data statistics. 

## Figures and Tables

**Table 1:** Measurements of three biochemical data results in three plant samples.

Samples	Chlorophyll Content (μg/g FW)	Reducing Sugar Content (μg/g FW)	Sucrose Content (mg/g FW)
CW (MS)	107 ± 8.7 c	0.56 ± 0.05 b	10.1 ± 0.98 c
35S:WRKY6-9 (MS)	132 ± 10.1 b	0.78 ± 0.06 a	13.2 ± 0.88 c
*wrky6-1* (MS)	113 ± 9.8 c	0.61 ± 0.05 b	11.5 ± 1.12 c
CW (LP)	136 ± 10.6 b	0.83 ± 0.07 a	28.3 ± 2.22 b
35S:WRKY6-9 (LP)	155 ± 11.3 a	0.88 ± 0.09 a	40.6 ± 3.65 a
*wrky6-1* (LP)	142 ± 10.4 a,b	0.86 ± 0.08 a	27.8 ± 2.37 b

Values followed by different lowercase letters means that data was significantly different among samples in the same treatment at 0.05 level. Bars represent the mean ± SE (*n* = 3).

**Table 2:** The 59 differentially expressed proteins identified in 35S:WRKY6-9.

Spot	Name	Score	Version	Pi/Mr (kDa)		Ttest (P)	Cov. (%)	Matched	Change Fold
				Experimental	Theoretical				
OL1	iPGAM	105	AAL09820.1	5.48/59	5.27/61	9.3 × 10^−3^	3	2	1.82
OL2	iPGAM	150	AAL09820.1	5.54/58	5.27/61	3.1 × 10^−5^	3	2	2.07
OL4	ATP synthase CF1 alpha subunit	200	NP_051044.1	5.29/56	5.19/55	1.5 × 10^−3^	7	3	2.82
OL5	Mitochondrial F1 ATP synthase beta subunit	339	CAC81058.1	5.65/54	6.52/64	2.4 × 10^−4^	10	5	1.58
OL7	AT4g23100/F7H19_290	228	AAL08228.1	5.38/54	6.16/59	9.7 × 10^−5^	6	3	1.68
OL8	S-adenosylmethionine synthetase like protein	291	BAF01944.1	5.7/54	5.60/43	5.9 × 10^−5^	10	3	2.06
OL10	Phosphoglycerate kinase, putative	519	AAM61185.1	5.62/41	5.49/42	5.6 × 10^−11^	24	6	1.99
OL11	Glutamine synthetase like protein	180	BAE98486.1	5.11/44	5.14/39	5.0 × 10^−9^	3	2	2.73
OL13	AT1G04410	50	BAH20341.1	5.79/53	6.59/26	3.4 × 10^−8^	8	1	1.96
OL14	Annexin-like protein	270	AAC49472.1	5.28/41	5.34/36	6.0 × 10^−5^	17	5	2.63
OL15	Annexin, partial	350	CAA67608.1	5.35/40	5.19/36	1.6 × 10^−4^	18	4	2.6
OL16	AT4G04640	299	BAH20433.1	6.68/35	5.06/17	1.4 × 10^−3^	31	3	2.12
OL18	AT3g44310/T10D17_100	149	AAL16248.1	6.2/35	5.78/39	3.6 × 10^−3^	7	3	2.16
OL19	AT4g38970/F19H22_70	133	AAL16224.1	5.68/36	6.79/43	7.6 × 10^−5^	7	2	−1.53
OL20	AT2G21330	111	BAH56881.1	5.39/37	6.34/41	7.3 × 10^−8^	6	2	−2.02
OL22	Mercaptopyruvate sulfurtransferase 1	197	NP_565203.1	5.17/37	5.95/42	2.1 × 10^−1^	9	3	2.15
OL23	AT3G16420	576	BAH57171.1	5.64/31	5.44/30	3.9 × 10^−7^	28	6	2.2
OL24	GF14chi isoform	354	AAA96254.1	4.68/30	4.68/30	5.8 × 10^−8^	22	6	1.5
OL26	AT5G14740	367	BAH20249.1	5.61/27	5.94/31	3.0 × 10^−6^	22	4	−2.69
OL27	Cytosolic triose phosphate isomerase	363	AAA03449.1	5.46/23	5.24/27	3.6 × 10^−6^	20	5	1.68
OL28	Cytosolic triose phosphate isomerase	513	AAA03449.1	5.66/22	5.24/27	8.0 × 10^−7^	20	5	1.77
OL29	AT3G01500	388	BAH56801.1	5.75/21	6.14/32	1.2 × 10^−4^	22	5	−2.98
OL30	AT3G01500	344	BAH56801.1	6.05/20	6.14/32	5.0 × 10^−6^	22	5	−4.04
OL31	AT5G54270	63	BAH56768.1	4.8/22	4.76/18	4.5 × 10^−5^	6	2	−1.89
OL32	Glutathione transferase	68	CAA72973.1	6.72/19	7.03/24	3.5 × 10^−7^	5	1	1.65
OL33	Ferritin 1	78	BAD94306.1	5.39/21	4.74/6	5.6 × 10^−6^	18	1	1.55
OL34	Glutathione S-transferase	297	AAG30126.1	6.35/20	6.31/24	4.6 × 10^−3^	18	4	3.89
OL35	Ferritin 1	107	AAM61077.1	5.27/21	5.73/28	4.1 × 10^−5^	7	2	−1.55
OL36	Fe-superoxide dismutase	159	AAA32791.1	6.48/19	6.30/25	4.2 × 10^−6^	15	3	1.71
OL37	Translationally-controlled tumor protein	115	AAM66134.1	4.62/19	4.52/19	2.0 × 10^−5^	11	2	1.52
OL39	MLP-like protein 423	281	NP_173813.1	5.2/16	5.1/17	1.3 × 10^−8^	29	3	2.04
OL40	Ribulose bisphosphate carboxylase	261	CAA32701.1	5.72/15	7.59/21	1.4 × 10^−9^	19	3	−2.26
OL41	Ribulose bisphosphate carboxylase	133	CAA32701.1	6.17/15	7.59/21	5.4 × 10^−7^	14	2	−2.37
OL42	Ribulose bisphosphate carboxylase	197	CAA32701.1	5.72/15	7.59/21	7.7 × 10^−6^	14	2	−2.07
OL43	Ribulose bisphosphate carboxylase	68	CAA32701.1	5.76/15	7.59/21	7.0 × 10^−9^	6	1	−1.71
OL44	Ribulose bisphosphate carboxylase	303	CAA31948.1	5.77/15	7.59/21	1.8 × 10^−8^	19	4	−1.7
OL45	Ribulose bisphosphate carboxylase	324	CAA31948.1	6.2/15	7.59/21	4.6 × 10^−8^	19	4	−1.63
OR1	PfkB-like carbohydrate kinase family protein	343	NP_199996.1	5.04/43	4.99/37	1.31 × 10^−2^	16	4	1.62
OR2	ACC oxidase	254	AAC27484.1	5.13/45	4.97/36	1.4 × 10^−2^	15	3	1.37
OR4	Chaperonin 10	111	AAC14026.1	5.52/26	8.86/27	1.42 × 10^−2^	7	2	1.27
OR5	Dehydroascorbate reductase	356	NP_173387.1	5.68/25	5.56/24	2.04 × 10^−2^	36	5	1.25
OR6	Glutathione S-transferase PHI 9	432	NP_180643.1	6.78/25	6.17/24	2.66 × 10^−2^	23	6	1.23
OR7	Mitochondrial chaperonin (HSP60)	260	AAC04902.1	5.55/61	5.30/56	2.66 × 10^−2^	11	4	1.23
OR8	TSA1	219	OAP06349.1	5.47/32	7.68/33	2.96 × 10^−2^	13	2	1.21
OR9	Putative cysteine proteinase AALP	161	AAN31820.1	5.03/28	6.26/39	3.22 × 10^−2^	9	3	−1.21
OR10	Putative lactoylglutathione lyase	292	AAL07227.1	5.21/32	5.11/32	3.26 × 10^−2^	16	5	−1.22
OR11	Glyoxalase II	455	AAB17995.1	5.37/33	5.58/28	3.27 × 10^−2^	24	5	−1.22
OR12	Mercaptopyruvate sulfurtransferase 1	343	NP_565203.1	5.19/39	5.95/42	3.37 × 10^−2^	12	4	−1.22
OR13	Transketolase family protein	409	NP_199898.1	5.36/40	5.67/39	3.61 × 10^−2^	18	5	−1.24
OR14	MLP-like protein 34	329	NP_850976.1	5.3/43	5.14/36	3.61 × 10^−2^	20	6	−1.25
OR15	NmrA-like negative transcriptional regulator	336	NP_565107.1	5.85/38	5.66/34	3.61 × 10^−2^	18	4	−1.26
OR16	GSTU26	106	OAP16650.1	5.63/30	5.53/26	3.62 × 10^−2^	8	2	−1.26
OR17	RCI1B	58	CAA52238.1	4.8/29	4.97/28	4.46 × 10^−2^	6	1	−1.29
OR18	Mannose-binding lectin superfamily protein	560	NP_188267.1	5.58/74	5.31/72	4.59 × 10^−2^	11	6	−1.31
OR19	F23N19.3	334	AAF19535.1	5.33/55	7.26/82	4.64 × 10^−2^	7	5	−1.31
OR20	GSTF8	341	OAP11759.1	6.39/24	8.50/29	4.72 × 10^−2^	22	6	−1.44
OR21	Ferritin 1	384	AAM61077.1	5.4/26	5.73/28	4.8 × 10^−2^	14	5	−1.44
OR22	Glutathione S transferase	526	AAM63854.1	6.28/26	6.08/24	4.81 × 10^−2^	36	6	-1.6
OR23	Glycine-rich RNA-binding protein 8	528	NP_195637.1	5.46/15	5.58/17	4.82 × 10^−2^	37	4	−2.16

iPGAM: 2,3-bisphosphoglycerate-independent phosphoglycerate mutase; AT4g23100/F7H19_290: Gamma-glutamylcysteine synthetase; AT1G04410: Malate dehydrogenase; AT4G04640: ATP synthase gamma; AT3g44310/T10D17_100: Nitrilase I; AT4g38970/F19H22_70: Fructose-bisphosphate aldolase; AT2G21330: Putative fructose bisphosphate aldolase; AT3G16420: PYK10-binding protein 1; AT5G14740: Carbonic anhydrase 2; AT3G01500: Carbonic anhydrase 1; AT5G54270: Light-harvesting chlorophyll B-binding protein; F23N19.3: Beta-fructofuranosidase; TSA1: Tryptophan synthase beta subunit.

## Supplementary Materials

Supplementary materials can be found at www.mdpi.com/1422-0067/18/5/1046/s1.
