# Supplementary material for: Identifying the Genes Regulated by AtWRKY6 Using Comparative Transcript and Proteomic Analysis under Phosphorus Deficiency
Source: Int J Mol Sci. 2017 May 12;18(5):1046. doi: 10.3390/ijms18051046 (PMC5454958; doi:10.3390/ijms18051046)
Supplement: Supplementary file 1 [file ijms-18-01046-s001.zip › Supplementary Figures.pdf]

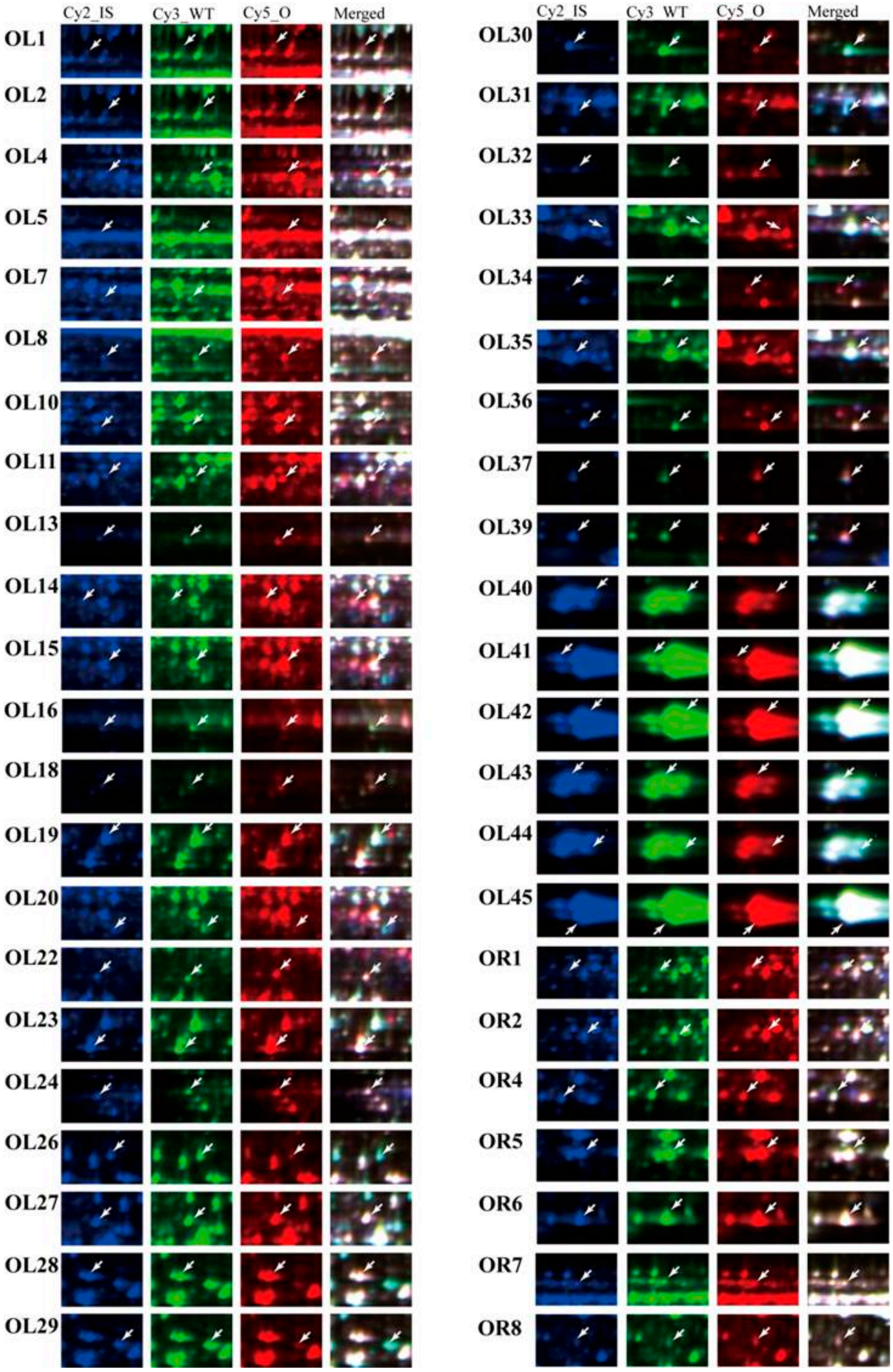


**Figure S1.** Some protein spots from leaves and roots of 35S:WRKY6-9 were listed.

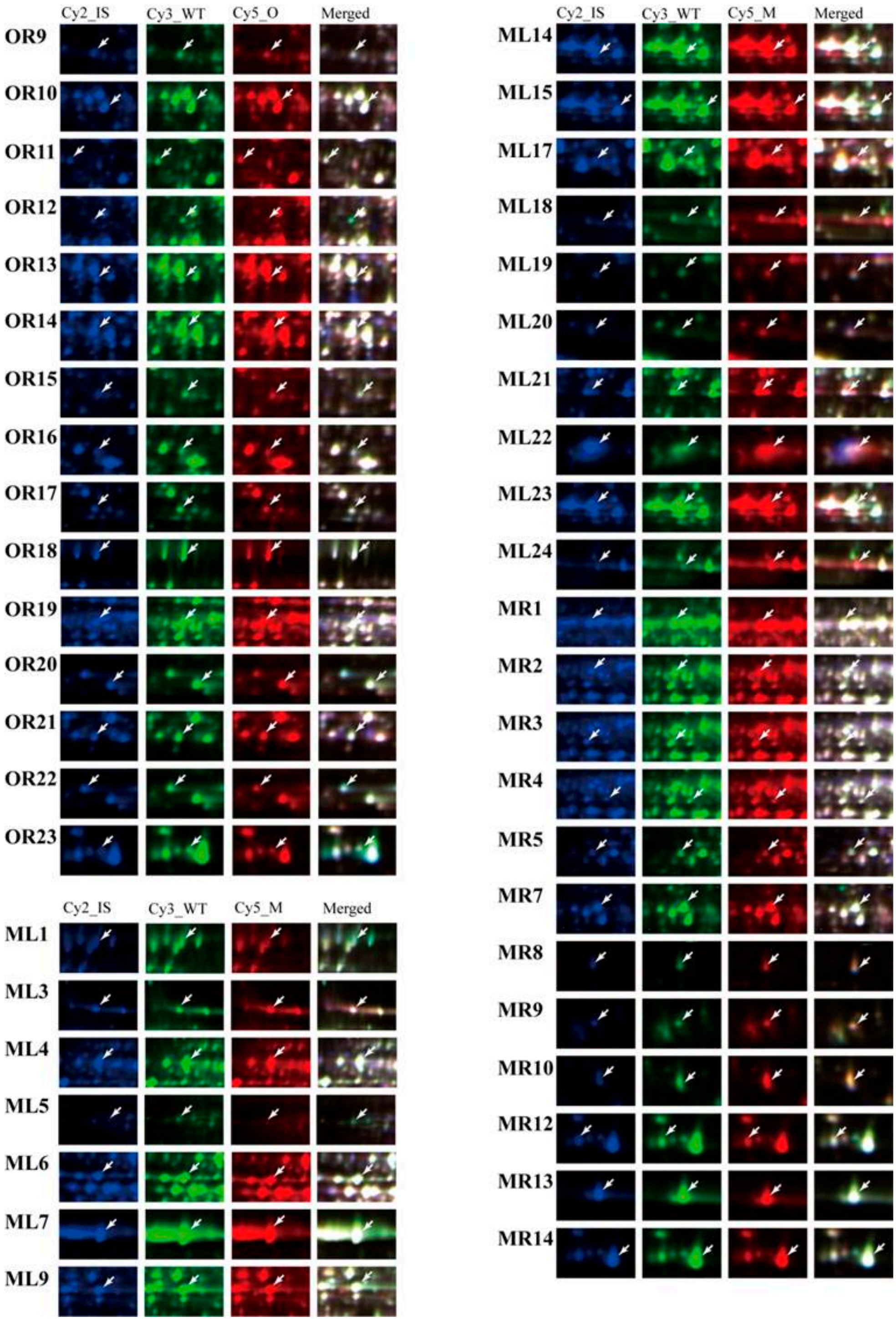


**Figure S2.** Some protein spots from leaves and roots of 35S:WRKY6-9 and *wrky6-1*were listed.
