# Supplementary material for: Identifying the Genes Regulated by AtWRKY6 Using Comparative Transcript and Proteomic Analysis under Phosphorus Deficiency
Source: Int J Mol Sci. 2017 May 12;18(5):1046. doi: 10.3390/ijms18051046 (PMC5454958; doi:10.3390/ijms18051046)
Supplement: Supplementary file 1 [file ijms-18-01046-s001.zip › Supplementary File S1 Mass spectrometry data.pdf]

Mass spectrometry data of 88 differentially abundant proteins

Spot No.: **OL1**

NCBI accession No.: **AAL09820.1**

Plant species: ***Arabidopsis thaliana***

Protein name: **Putative 2,3-bisphosphoglycerate-independent phosphoglycerate mutase**

Peptide sequences: UK.ALEYEDFDKFDR.V ; UR.YLVSPPEIDR.T

PFF Mascot score: **[105]** Sequence coverage %: **[3]**

Matched peptides No.: **[2]**

Calculated Mr: **60671** Calculated *p*I: **5.27**

Annotated PFF **spectra:**

Ions score is -10*Log(P), where P is the probability that the observed match is a random event.
Individual ions scores > 35 indicate identity or extensive homology (p<0.05).
Protein scores are derived from ions scores as a non-probabilistic basis for ranking protein hits.

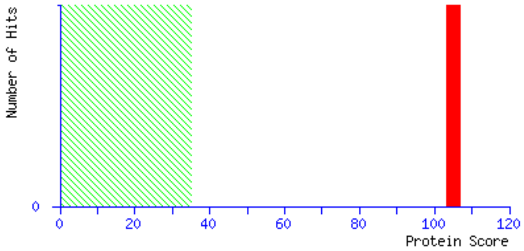

Matched peptide sequences: shown in Bold Red

```
  1 MATSSAWKLD DHPKLPKGKT IAVIVLDGWG ESAPDQYNCI HNAPTPAMDS
 51 LKHGAPDTWT LIKAHGTAVG LPSEDDMGNS EVGHNALGAG RIFAQGAKLC
101 DQALASGKIF EGEGFKYVSE SFETNTLHLV GLLSDGGVHS RLDQLQLLIK
151 GSAERGAKRI RVHILTDGRD VLDGSSVGFV ETLEADLVAL RENGVDAQIA
201 SGGGRMYVTL DRYENDWEVV KRGWDAQVLG EAPHKFKNAV EAVKTLRKEP
251 GANDQYLPPF VIVDESGKAV GPIVDGDAVV TFNFRADRMV MHAKALEYED
301 FDKFDRVRYP KIRYAGMLQY DGELKLPSRY LVSPPEIDRT SGEYLTHNGV
351 STFACSSSCL LIIGLYYALH FCQYETVKFG HVTFFWNGNR SGYFNEKLEE
401 YVEIPSDSGI SFNVQPKMKA LEIGEKARDA ILSGKFDQVR VNIPNGDMVG
451 HTGDIEATVV ACEAADLAVK MIFDAIEQVK GIYVVTADHG NAEDMVKRDK
501 SGKPALDKEG KLQILTSHTL KPVPIAIGGP GLAQGVRFRK DLETPGLANV
551 AATVMNLHGF VAPSDYEPTL IEVVE
```

Spot No.: **OL2**

NCBI accession No.: **AAL09820.1**

Plant species: ***Arabidopsis thaliana***

Protein name： **Putative 2,3-bisphosphoglycerate-independent phosphoglycerate mutase**

Peptide sequences:K.ALEYEDFDKFDR.V; R.YLVSPPEIDR.T

PFF Mascot score: **[150]**   Sequence coverage %: **[3]**

Matched peptides No.: **[2]**

Calculated Mr: **60671**   Calculated *p*I: **5.27**

Annotated PFF **spectra:**

Ions score is -10*Log(P), where P is the probability that the observed match is a random event.
Individual ions scores > 35 indicate identity or extensive homology (p<0.05).
Protein scores are derived from ions scores as a non-probabilistic basis for ranking protein hits.

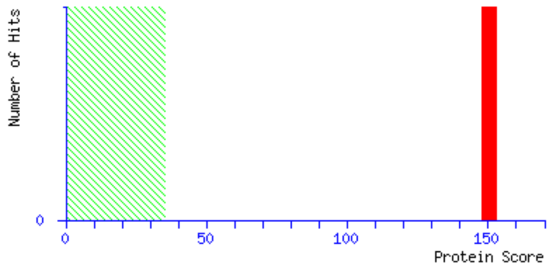

Matched peptide sequences: shown in Bold Red

```
  1 MATSSAWKLD DHPKLPKGKT IAVIVLDGWG ESAPDQYNCI HNAPTPAMDS
 51 LKHGAPDTWT LIKAHGTAVG LPSEDDMGNS EVGHNALGAG RIFAQGAKLC
101 DQALASGKIF EGEGFKYVSE SFETNTLHLV GLLSDGGVHS RLDQLQLLIK
151 GSAERGAKRI RVHILTDGRD VLDGSSVGFV ETLEADLVAL RENGVDAQIA
201 SGGGRMYVTL DRYENDWEVV KRGWDAQVLG EAPHKFKNAV EAVKTLRKEP
251 GANDQYLPPF VIVDESGKAV GPIVDGDAVV TFNFRADRMV MHAKALEYED
301 FDKFDRVRYP KIRYAGMLQY DGELKLPSRY LVSPPEIDRT SGEYLTHNGV
351 STFACSSSCL LIIGLYYALH FCQYETVKFG HVTFFWNGNR SGYFNEKLEE
401 YVEIPSDSGI SFNVQPKMKA LEIGEKARDA ILSGKFDQVR VNIPNGDMVG
451 HTGDIEATVV ACEAADLAVK MIFDAIEQVK GIYVVTADHG NAEDMVKRDK
501 SGKPALDKEG KLQILTSHTL KPVPIAIGGP GLAQGVRFRK DLETPGLANV
551 AATVMNLHGF VAPSDYEPTL IEVVE
```

Spot No.: **OL4**

NCBI accession No.: **NP_051044.1**

Plant species: ***Arabidopsis thaliana***

Protein name： **ATP synthase CF1 alpha subunit**

Peptide sequences:K.IAQIPVSEAYLGR.V; R.VINALANPIDGR.G; R.EAYPGDVFYLHSR.L

PFF Mascot score: **[200]**   Sequence coverage %: **[7]**

Matched peptides No.: **[3]**

Calculated Mr: **55351**   Calculated *p*I: **5.19**

Annotated PFF **spectra:**

Ions score is -10*Log(P), where P is the probability that the observed match is a random event.
Individual ions scores > 35 indicate identity or extensive homology (p<0.05).
Protein scores are derived from ions scores as a non-probabilistic basis for ranking protein hits.

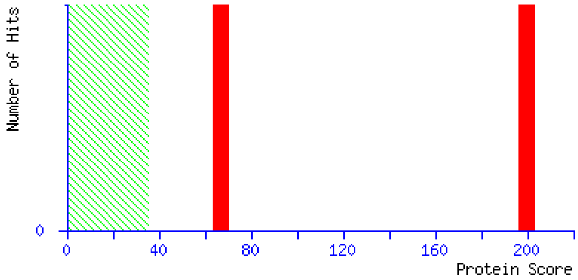

Matched peptide sequences: shown in Bold Red

```
  1 MVTIRADEIS NIIRERIEQY NREVTIVNTG TVLQVGDGIA RIYGLDEVMA
 51 GELVEFEEGT IGIALNLESN NVGVVLMGDG LMIQEGSSVK ATGKIAQIPV
101 SEAYLGRVIN ALANPIDGRG KISASESRLI ESPAPGIISR RSVYEPLQTG
151 LIAIDSMIPI GRGQRELIIG DRQTGKTAVA TDTILNQQGQ NVICVYVAIG
201 QKASSVAQVV TSLQERGAME YTIVVAETAD SPATLQYLAP YTGAALAEYF
251 MYREQHTLII YDDLSKQAQA YRQMSLLLRR PPGREAYPGD VFYLHSRLLE
301 RAAKLSSQLG EGSMTALPIV ETQSGDVSAY IPTNVISITD GQIFLSADLF
351 NAGIRPAINV GISVSRVGSA AQIKAMKQVA GKLKLELAQF AELEAFSQFS
401 SDLDKATQNQ LARGQRLREL LKQSQSAPLT VEEQIMTIYT GTNGYLDGLE
451 IGQVRKFLVQ LRTYLKTNKP QFQEIIASTK TLTAEAESFL KEGIQEQLER
501 FLLQEKV
```

Spot No.: **OL5**

NCBI accession No.: **CAC81058.1**

Plant species: ***Arabidopsis thaliana***

Protein name: **mitochondrial F1 ATP synthase beta subunit**

Peptide sequences: R.TIAMDGTEGLVR.G;

R.VCQVIGAIVDVR.F; K.VVDLLAPYQR.G;

R.VGLTGLTVAEYFR.D; R.FTQANSEVSALLGR.I

PFF Mascot score: **[339]** Sequence coverage %: **[10]**

Matched peptides No.: **[5]**

Calculated Mr: **63560** Calculated *p*I: **6.52**

Annotated PFF **spectra:**

Ions score is -10*Log(P), where P is the probability that the observed match is a random event.
Individual ions scores > 35 indicate identity or extensive homology (p<0.05).
Protein scores are derived from ions scores as a non-probabilistic basis for ranking protein hits.

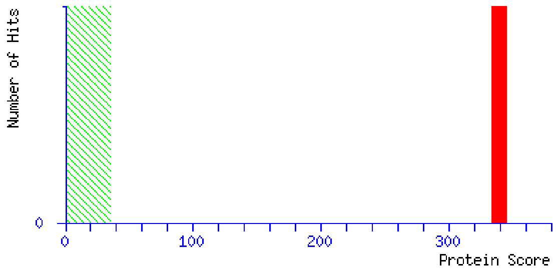

Matched peptide sequences: shown in Bold Red

```
  1 MTMITPSSNT THYRESWYAC RYRSGIPGST HASVASRRVL SSLLRSSSGR
 51 SAAKLGNRNP RLPSPSPARH AAPCSYLLGR VAEYATSSPA SSAAPSSAPA
101 KDEGKKTYDY GGKGAIGRVC QVIGAIVDVR FEDQEGLPPI MTSLEVQDHP
151 TRLVLEVSHH LGQNVVRTIA MDGTEGLVRG RKVLNTGAPI TVPVGRATLG
201 RIMNVLGEPI DERGEIKTEH YLPIHRDAPA LVDLATGQEI LATGIKVVDL
251 LAPYQRGGKI GLFGGAGVGK TVLIMELINN VAKAHGGFSV FAGVGERTRE
301 GNDLYREMIE SGVIKLGEKQ SESKCALVYG QMNEPPGARA RVGLTGLTVA
351 EYFRDAEGQD VLLFIDNIFR FTQANSEVSA LLGRIPSAVG YQPTLASDLG
401 ALQERITTTK KGSITSVQAI YVPADDLTDP APATTFAHLD ATTVLSRQIS
451 ELGIYPAVDP LDSTSRMLSP HILGEEHYNT ARGVQKVLQN YKNLQDIIAI
501 LGMDELSEDD KLTVARARKI QRFLSQPFHV AEIFTGAPGK YVDLKENINS
551 FQGLLDGKYD DLSEQSFYMV GGIDEVVAKA EKIAKESAA
```

Spot No.: **OL7**

NCBI accession No.: **AAL08228.1**

Plant species: ***Arabidopsis thaliana***

Protein name： **AT4g23100/F7H19_290**

Peptide sequences:R.SHIWTDTDKDR.T; K.YIDCTGMTFR.Q; K.EAGFLNAVDEVVR.T

PFF Mascot score: **[228]** Sequence coverage %: **[6]**

Matched peptides No.: **[3]**

Calculated Mr: **58938** Calculated *p*I: **6.16**

Annotated PFF **spectra:**

Ions score is -10*Log(P), where P is the probability that the observed match is a random event.
Individual ions scores > 35 indicate identity or extensive homology (p<0.05).
Protein scores are derived from ions scores as a non-probabilistic basis for ranking protein hits.

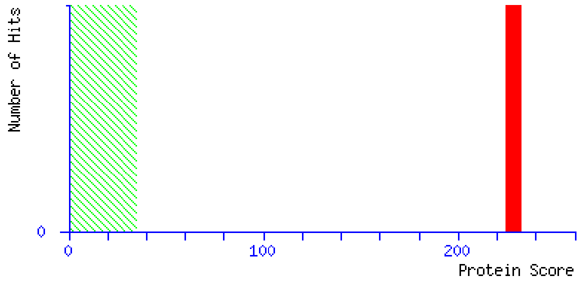

Matched peptide sequences: shown in Bold Red

```
  1 MALLSQAGGS YTVVPSGVCS KTGTKAVVSG GVRNLDVLRM KEAFGSSNSR
 51 SLSTKSMLLH SVKRSKRGHQ LIVAASPPTE EAVVATEPLT REDLIAYLAS
101 GCKTKDKYRI GTEHEKFGFE VNTLRPMKYD QIAELLNGIA ERFEWEKVME
151 GDKIIGLKQG KQSISLEPGG QFELSGAPLE TLHQTCAEVN SHLYQVKAVA
201 EEMGIGFLGI GFQPKWRRED IPIMPKGRYD IMRNYMPKVG TLGLDMMLRT
251 CTVQVNLDFS SEADMIRKFR AGLALQPIAT ALFANSPFTE GKPNGFLSMR
301 SHIWTDTDKD RTGMLPFVFD DSFGFEQYVD YALDVPMYFA YRKNKYIDCT
351 GMTFRQFLAG KLPCLPGELP SYNDWENHLT TIFPEVRLKR YLEMRGADGG
401 PWRRLCALPA FWVGLLYDDD SLQAILDLTA DWTPAEREML RNKVPVTGLK
451 TPFRDGLLKH VAEDVLKLAK DGLERRGYKE AGFLNAVDEV VRTGVTPAEK
501 LLEMYNGEWG QSVDPVFEEL LY
```

Spot No.: **OL8**

NCBI accession No.: **BAF01944.1**

Plant species: ***Arabidopsis thaliana***

Protein name： **S-adenosylmethionine synthase like protein**

Peptide sequences: K.ANVDYEQIVR.K;

R.EIGFVSADVGLDADNCK.V; R.FVIGGPHGDAGLTGR.K

PFF Mascot score: **[291]** Sequence coverage %: **[10]**

Matched peptides No.: **[3]**

Calculated Mr: **43110** Calculated *p*I: **5.60**

Annotated PFF **spectra:**

Ions score is -10*Log(P), where P is the probability that the observed match is a random event.
Individual ions scores > 35 indicate identity or extensive homology (p<0.05).
Protein scores are derived from ions scores as a non-probabilistic basis for ranking protein hits.

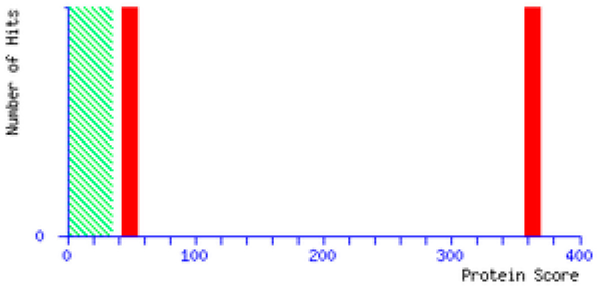

Matched peptide sequences: shown in Bold Red

```
  1 MESFLFTSES VNEGHPDKLC DQISDAILDA CLEQDPESKV ACETCTKTNM
 51 VMVFGEITTK ANVDYEQIVR KTCREIGFVS ADVGLDADNC KVLVNIEQQS
101 PDIAQGVHGH LTKKPEEVGA GDQGHMFGYA TDETPELMPL THVLATKLGA
151 KLTEVRKNGT CPWLRPDGKT QVTIEYINES GAMVPVRVHT VLISTQHDET
201 VTNDEIAADL KEHVIKPVIP EKYLDEKTIF HLNPSGRFVI GGPHGDAGLT
251 GRKIIIDTYG GWGAHGGGAF SGKDPTKVDR SGAYIVRQAA KSIVASGLAR
301 RVIVQVSYAI GVPEPLSVFV DSYGTGKIPD KEILEIVKES FDFRPGMISI
351 NLDLKRGGNG RFLKTAAYGH FGRDDADFTW EVVKPLKSNK VQA
```

Spot No.: **OL10**

NCBI accession No.:**AAM61185.1**

Plant species: ***Arabidopsis thaliana***

Protein name：**phosphoglycerate kinase**

Peptide sequences: R.VTLFLNLANDPTIER.I; R.VDLNVPLDDNSNITDDTR.I;K.LVAGLPEGGVLLLENVR.F;R.FYAEEEKNDPEFAK.K;K.LAALADVYVNDAFGTAHR.A;K.ELDYLVGAVANPK.K;K.AQGLSVGSSLVEEDKLDLAK.S

PFF Mascot score: **[519]**

Sequence coverage %: **[24]**

Matched peptides No.: **[6]**

Calculated Mr: **42178**

Calculated *p*I: **5.49**

Annotated PFF **spectra:**

Ions score is -10*Log(P), where P is the probability that the observed match is a random event.
Individual ions scores > 35 indicate identity or extensive homology (p<0.05).
Protein scores are derived from ions scores as a non-probabilistic basis for ranking protein hits.

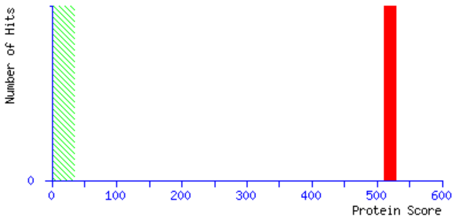

Matched peptide sequences: shown in Bold Red

```
  1 MATKRSVGTL KEADLKGKSV FVRVDLNVPL DDNSNITDDT RIRAAVPTIK
 51 YLMGNGSRVV LCSHLGRPKG VTPKYSLKPL VPRLSELLGV EVVMANDSIG
101 EEVQKLVAGL PEGGVLLLEN VRFYAEEEKN DPEFAKKLAA LADVYVNDAF
151 GTAHRAHAST EGVAKFLKPS VAGFLMQKEL DYLVGAVANP KKPFAAIVGG
201 SKVSTKIGVI ESLLNTVDIL LLGGGMIFTF YKAQGLSVGS SLVEEDKLDL
251 AKSLMEKAKA KGVSLLLPTD VVIADKFAPD ANSKIVPATA IPDGWMGLDI
301 GPDSIKTFSE ALDTTKTIIW NGPMGVFEFD KFAAGTEAVA KQLAELSGKG
351 VTTIIGGGDS VAAVEKVGLA DKMSHISTGG GASLELLEGK PLPGVLALDE
401 A
```

Spot No.: **OL11**

NCBI accession No.: **BAE98486.1**

Plant species: ***Arabidopsis thaliana***

Protein name: **glutamine synthetase like protein**

Peptide sequences: R.HKEHISAYGEGNER.R; K.EHISAYGEGNER.R

PFF Mascot score: **[180]** Sequence coverage %: **[3]**

Matched peptides No.: **[2]**

Calculated Mr: **39323** Calculated *p*I: **5.14**

Annotated PFF **spectra:**

Ions score is -10*Log(P), where P is the probability that the observed match is a random event.
Individual ions scores > 35 indicate identity or extensive homology (p<0.05).
Protein scores are derived from ions scores as a non-probabilistic basis for ranking protein hits.

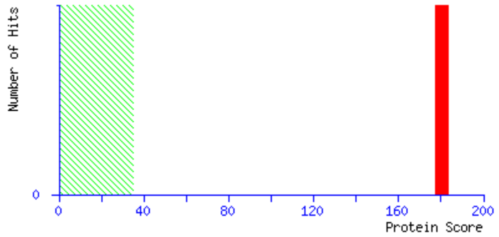

Matched peptide sequences: shown in **Bold Red**

```
  1 MSLLADLVNL DISDNSEKII AEYIWVGGSG MDMRSKARTL PGPVTDPSKL
 51 PKWNYDGSST GQAPGQDSEV ILYPQAIFKD PFRRGNNILV MCDAYTPAGE
101 PIPTNKRHAA AEIFANPDVI AEVPWYGIEQ EYTLLQKDVN WPLGWPIGGF
151 PGPQGPYYCS IGADKSFGRD IVDAHYKASL YAGINISGIN GEVMPGQWEF
201 QVGPSVGISA ADEIWIARYI LERITEIAGV VVSFDPKPIP GDWNGAGAHT
251 NYSTKSMREE GGYEIIKKAI EKLGLRHKEH ISAYGEGNER RLTGHHETAD
301 INTFLWGVAN RGASIRVGRD TEKEGKGYFE DRRPASNMDP YVVTSMIAET
351 TLLWNP
```

Spot No.: **OL13**

NCBI accession No.: **BAH20341.1**

Plant species: ***Arabidopsis thaliana***

Protein name: **AT1G04410, partial**

Peptide sequences: R.ELVKDDAWLDGEFISTVQQR.G

PFF Mascot score: **[50]** Sequence coverage %: **[8]**

Matched peptides No.: **[1]**

Calculated Mr: **26347** Calculated *p*I: **6.59**

Annotated PFF **spectra:**

Ions score is -10*Log(P), where P is the probability that the observed match is a random event.
Individual ions scores > 35 indicate identity or extensive homology (p<0.05).
Protein scores are derived from ions scores as a non-probabilistic basis for ranking protein hits.

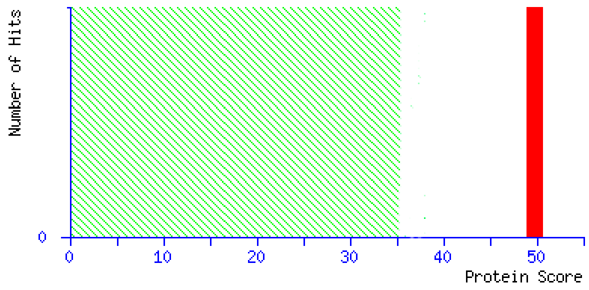

Matched peptide sequences: shown in Bold Red

```
  1 MAKEPVRVLV TGAAGQIGYA LVPMIARGIM LGADQPVILH MLDIPPAAEA
 51 LNGVKMELID AAFPLLKGVV ATTDAVEGCT GVNVAVMVGG FPRKEGMERK
101 DVMSKNVSIY KSQAAALEKH AAPNCKVLVV ANPANTNALI LKEFAPSIPE
151 KNISCLTRLD HNRALGQISE RLSVPVSDVK NVIIWGNHSS SQYPDVNHAK
201 VQTSSGEKPV RELVKDDAWL DGEFISTVQQ RGAAIIKARK LSSALSAASS
251 ACDHIRDWVL GTPEGTFVSM GVYSDGSYSV PSGLIYSFPV TCRNGDWSIV
301 QGLPIDEVSR KKMDLTAEEL KEEKDLAYSC LS
```

Spot No.: **OL14**

NCBI accession No.: **AAC49472.1**

Plant species: ***Arabidopsis thaliana***

Protein name： **Annexin-like protein**

Peptide sequences:K.TLDKELSNDFER.A; R.AILLWTLEPGER.D;K.SLEEDVAHHTTGDFR.K;K.HYNDEDVIR.I;K.AQINATFNR.Y

PFF Mascot score: **[270]** Sequence coverage %: **[17]**

Matched peptides No.: **[5]**

Calculated Mr: **36322** Calculated *p*I: **5.34**

Annotated PFF **spectra:**

Ions score is -10*Log(P), where P is the probability that the observed match is a random event.
Individual ions scores > 35 indicate identity or extensive homology (p<0.05).
Protein scores are derived from ions scores as a non-probabilistic basis for ranking protein hits.

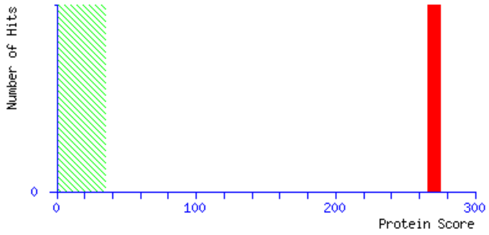

Matched peptide sequences: shown in **Bold Red**

1 MATLKVSDSV PAPSDDAEQL RTAFEGWGTN EDLIISILAH RSAEQRKVIR
51 QAYHETYGKD LLK**TLDKELS** **NDFERAILLW** **TLEPGER**DAL LANEATKRWT
101 SSNQVLMEVA CTRTSTQLLH ARQAYHARYK K**SLEEDVAHH** **TTGDFR**KLLV
151 SLVTSYRYEG DEVNMTLAKQ EAKLVHEKIK DK**HYNDEDVI** **R**ILSTRSK**AQ**
201 **INATFNR**YQD DHGEEILKSL EEGDDDDKLP CTLRSTIQCL TRPELYFVDV
251 LRSAINKTGT DEGALTRIVT TRAEIDLKVI GEEYQRRNSI PLEKAITKDT
301 RGDYEKMLVA LLGEDDA

Spot No.: **OL15**

NCBI accession No.: **CAA67608.1**

Plant species: ***Arabidopsis thaliana***

Protein name： **Annexin, partial**

Peptide sequences: K.VSDSVPAPSDDAEQLR.T;

R.AILLWTLEPGER.D; K.SLEEDVAHHTTGDFR.K;

K.SLEEGDDDDKFLALLR.S

PFF Mascot score: **[350]**   Sequence coverage %: **[18]**

Matched peptides No.: **[4]**

Calculated Mr: **35928**   Calculated *p*I: **5.19**

Annotated PFF **spectra:**

Ions score is -10*Log(P), where P is the probability that the observed match is a random event.
Individual ions scores > 35 indicate identity or extensive homology (p<0.05).
Protein scores are derived from ions scores as a non-probabilistic basis for ranking protein hits.

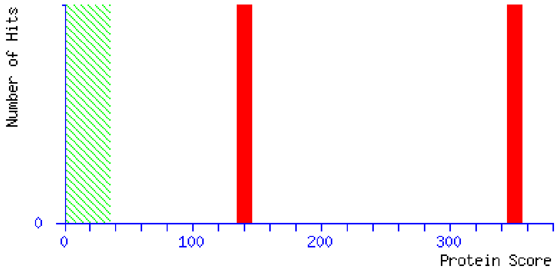

Matched peptide sequences: shown in Bold Red

```
  1 TLKVSDSVPA PSDDAEQLRT AFEGWGTNED LIISILAHRS AEQRKVIRQA
 51 YHETYGEDLL KTLDKELSND FERAILLWTL EPGERDALLA NEATKRWTSS
101 NQVLMEVACT RTSTQLLHAR QAYHARYKKS LEEDVAHHTT GDFRKLLVSL
151 VTSYRYEGDE VNMTLAKQEA KLVHEKIKDK HYNDEDVIRI LSTISKAQIN
201 ATFNRYQDDH GEEILKSLEE GDDDDKFLAL LRSTIQCLTR PELYFVDVLR
251 SAINKTGTDE GALTRIVTTR AEIDLKVIGE EYQRRNSIPL RAITKDNCGD
301 YGKMLVAIFG EDDA
```

Spot No.: **OL16**

NCBI accession No.: **BAH20433.1**

Plant species: ***Arabidopsis thaliana***

Protein name： **AT4G04640, partial**

Peptide sequences: K.SEPVIHTLLPLSPK.G; K.GEICDINGTCVDAAEDEFFR.L; R.ALQESLASELAAR.M

PFF Mascot score: **[299]** Sequence coverage %: **[31]**

Matched peptides No.: **[3]**

Calculated Mr: **16738** Calculated *p*I: **5.06**

Annotated PFF spectra:

Ions score is -10*Log(P), where P is the probability that the observed match is a random event.
Individual ions scores > 35 indicate identity or extensive homology (p<0.05).
Protein scores are derived from ions scores as a non-probabilistic basis for ranking protein hits.

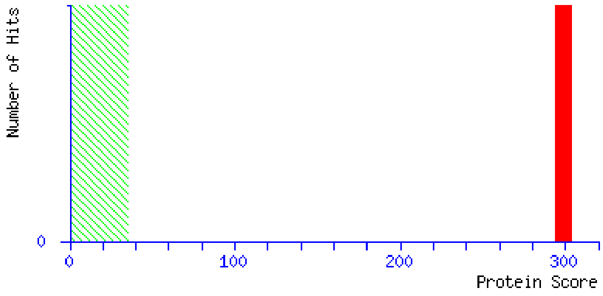

Matched peptide sequences: shown in **Bold Red**

```
  1 YTKFVSLVKS EPVIHTLLPL SPKGEICDIN GTCVDAAEDE FFRLTTKEGK
 51 LTVERETFRT PTADFSPILQ FEQDPVQILD ALLPLYLNSQ ILRALQESLA
101 SELAARMSAM SSASDNASDL KKSLSMVYNR KRQAKITGEI LEIVAGANAQ
151 V
```

Spot No.: **OL18**

NCBI accession No.: **AAL16248.1**

Plant species: ***Arabidopsis thaliana***

Protein name: **AT3g44310/T10D17_100**

Peptide sequences: R.KYHASAIHVPGPEVAR.L;

K.YHASAIHVPGPEVAR.L; K.LGAAICWENR.M

PFF Mascot score: **[149]** Sequence coverage %: **[7]**

Matched peptides No.: **[3]**

Calculated Mr: **38553** Calculated *p*I: **5.78**

Annotated PFF **spectra:**

Ions score is -10*Log(P), where P is the probability that the observed match is a random event.
Individual ions scores > 35 indicate identity or extensive homology (p<0.05).
Protein scores are derived from ions scores as a non-probabilistic basis for ranking protein hits.

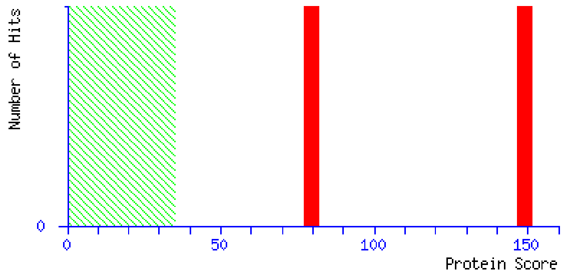

Matched peptide sequences: shown in **Bold Red**

```
  1 MSSTKDMSTV QNATPFNGVA PSTTVRVTIV QSSTVYNDTP ATIDKAEKYI
 51 VEAASKGAEL VLFPEGFIGG YPRGFRFGLA VGVHNEEGRD EFRKYHASAI
101 HVPGPEVARL ADVARKNHVY LVMGAIEKEG YTLYCTVLFF SPQGQFLGKH
151 RKLMPTSLER CIWGQGDGST IPVYDTPIGK LGAAICWENR MPLYRTALYA
201 KGIELYCAPT ADGSKEWQSS MLHIAIEGGC FVLSACQFCQ RKHFPDHPDY
251 LFTDWYDDKE HDSIVSQGGS VIISPLGQVL AGPNFESEGL VTADIDLGDI
301 ARAKLYFDSV GHYSRPDVLH LTVNEYPRKS VTFVTKVEKA EDDSNK
```

Spot No.: **OL19**

NCBI accession No.: **AAL16224.1**

Plant species: ***Arabidopsis thaliana***

Protein name: **AT4g38970/F19H22_70**

Peptide sequences: R.TAAYYQQGAR.F; K.TWGGRPENVNAAQTTLLAR.A

PFF Mascot score: **[133]** Sequence coverage %: **[7]**

Matched peptides No.: **[2]**

Calculated Mr: **43029** Calculated *p*I: **6.79**

Annotated PFF **spectra:**

Ions score is -10*Log(P), where P is the probability that the observed match is a random event.
Individual ions scores > 35 indicate identity or extensive homology (p<0.05).
Protein scores are derived from ions scores as a non-probabilistic basis for ranking protein hits.

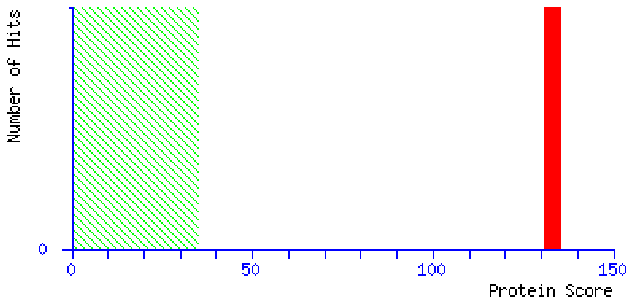

Matched peptide sequences: shown in Bold Red

```
  1 MASTSLLKAS PVLDKSEWVK GQSVLFRQPS SASVVLRNRA TSLTVRAASS
 51 YADELVKTAK TIASPGRGIL AMDESNATCG KRLDSIGLEN TEANRQAFRT
101 LLVSAPGLGQ YVSGAILFEE TLYQSTTEGK KMVDVLVEQN IVPGIKVDKG
151 LVPLVGSNNE SWGQGLDGLS SRTAAYYQQG ARFAKWRTVV SIPNGPSALA
201 VKEAAWGLAR YAAISQDSGL VPIVEPEILL DGEHDIDRTY DVAEKVWAEV
251 FFYLAQNNVM FEGILLKPSM VTPGAESKDR ATPEQVAAYT LKLLRNRVPP
301 AVPGIMFLSG GQSEVEATLN LNAMNQAPNP WHVSFSYARA LQNTCLKTWG
351 GRPENVNAAQ TTLLARAKAN SLAQLGKYTG EGESEEAKEG MFVKGYTY
```

Spot No.: **OL20**

NCBI accession No.: **BAH56881.1**

Plant species: ***Arabidopsis thaliana***

Protein name： **AT2G21330**

Peptide sequences: R.LASIGLENTEANR.Q; R.TAAYYQQGAR.F

PFF Mascot score: **[111]** Sequence coverage %: **[6]**

Matched peptides No.: **[2]**

Calculated Mr: **40612** Calculated *p*I: **6.34**

Annotated PFF **spectra:**

Ions score is -10*Log(P), where P is the probability that the observed match is a random event. Individual ions scores > 35 indicate identity or extensive homology (p<0.05).
Protein scores are derived from ions scores as a non-probabilistic basis for ranking protein hits.

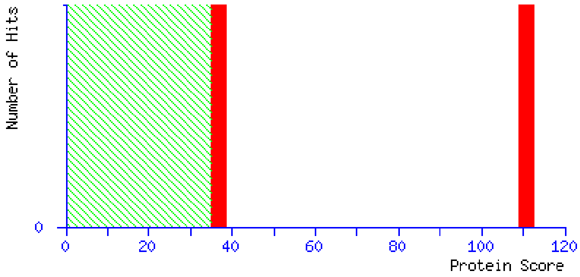

Matched peptide sequences: shown in **Bold Red**

```
  1 MASSTATMLK ASPVKSDWVK GQSLLLRQPS SVSAIRSHVA PSALTVRAAS
 51 AYADELVKTA KTIASPGHGI MAMDESNATC GKRLASIGLE NTEANRQAYR
101 TLLVSAPGLG QYISGAILFE ETLYQSTTDG KKMVDVLVEQ NIVPGIKVDK
151 GLVPLVGSYD ESWCQGLDGL ASRTAAYYQQ GARFAKWRTV VSIPNGPSAL
201 AVKEAAWGLA RYAAISQDSG LVPIVEPEIM LDGEHGIDRT YDVAEKVWAE
251 VFFYLAQNNV MFEGILLKPS MVTPGAEATD RATPEQVASY TLKLLRNRIP
301 PAVPGIMFLS GGQSELEATL NLNAMNQAPN TCLAEHLLED MGRQGREREG
351 GSGHSLGQSQ SQFAGSAREI HWRRRV
```

Spot No.: **OL22**

NCBI accession No.: **NP_565203.1**

Plant species: ***Arabidopsis thaliana***

Protein name：**mercaptopyruvate sulfurtransferase 1**

Peptide sequences: R.NPIQEYQVAHIPR.A; R.ALFFDLDGISDR.K; K.VWVLDGGLPR.W

PFF Mascot score: **[197]** Sequence coverage %: **[9]**

Matched peptides No.: **[3]**

Calculated Mr: **42152** Calculated *p*I: **5.95**

Annotated PFF spectra:

Ions score is -10*Log(P), where P is the probability that the observed match is a random event. Individual ions scores > 35 indicate identity or extensive homology (p<0.05). Protein scores are derived from ions scores as a non-probabilistic basis for ranking protein hits.

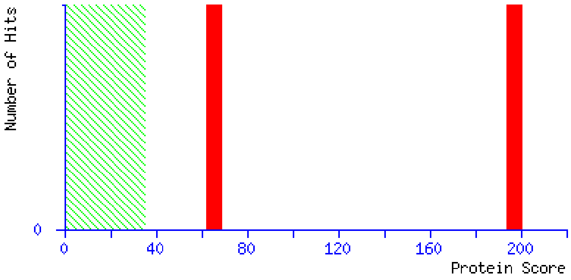

Matched peptide sequences: shown in Bold Red

```
  1 MASTLFSRTF LAASHRLITP SLPQKIFNPA TFLSRSLHSQ LGSASTAYKS
 51 TTWARRAMAS TGVETKAGYS TSSVSTSEPV VSVDWLHANL REPDLKILDA
101 SWYMPDEQRN PIQEYQVAHI PRALFFDLDG ISDRKTSLPH MLPTEEAFAA
151 GCSALGIDNK DEVVVYDGKG IFSAARVWWM FRVFGHEKVW VLDGGLPRWR
201 ASGYDVESSA SGDAILKASA ASEAIEKIYQ GQTVSPITFQ TKFQPHLVWT
251 LDQVKNNMED PTYQHIDARS KARFDGTAPE PRKGIRSGHI PGSKCIPFPQ
301 MFDSCNTLLP AEELKKRFDQ EDISLDKPIM ASCGTGVTAC ILAMGLHRLG
351 KTDVPIYDGS WTEWATQPDL PIESVESSS
```

Spot No.: **OL23**

NCBI accession No.: **BAH57171.1**

Plant species: ***Arabidopsis thaliana***

Protein name: **AT3G16420**

Peptide sequences: K.GANLWDDGSTHDAVTK.I;

K.QLTAFGSDDGTVWDDGAYVGVK.K;

K.KVYVGQAQDGISAVK.F; K.VYVGQAQDGISAVK.F;

K.FVYDKSPEEVTGEEHGK.S; K.IGVHVRPLSN.-

PFF Mascot score: **[576]** Sequence coverage %: **[28]**

Matched peptides No.: **[6]**

Calculated Mr: **29808** Calculated *p*I: **5.44**

Annotated PFF **spectra:**

Ions score is -10*Log(P), where P is the probability that the observed match is a random event.
Individual ions scores > 35 indicate identity or extensive homology (p<0.05).
Protein scores are derived from ions scores as a non-probabilistic basis for ranking protein hits.

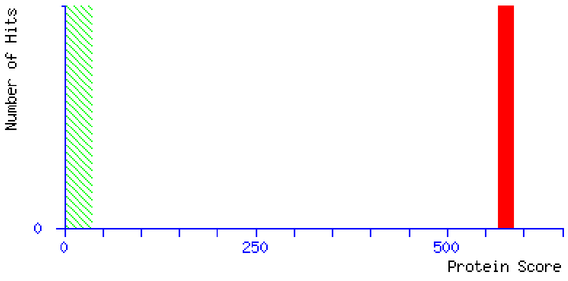

Matched peptide sequences: shown in Bold Red

```
  1 MAQKVEAQGG KGANLWDDGS THDAVTKIQL AAGIDGIQYV QFDYVKNGQP
 51 EQAPLRGTKG RVLPADPFVI NHPDEHLVSV EGWYSPEGTH FTLQVKDKKI
101 IGFHGSAGGN LNSLGAYFAP LTTTTPLTPA KQLTAFGSDD GTVWDDGAYV
151 GVKKVYVGQA QDGISAVKFV YDKSPEEVTG EEHGKSTLLG FEEFVLDYPS
201 EYITAVDGTY DKIFGSDGSV ITMLRFKTNK QTSPPFGLEA GTVFELKEEG
251 HKIVGFHGRA DVLLHKIGVH VRPLSN
```

Spot No.: **OL24**

NCBI accession No.: **AAA96254.1**

Plant species: ***Arabidopsis thaliana***

Protein name: **GF14chi isoform**

Peptide sequences: K.AVDKDELTVEER.N; R.GNDDHVSLIR.D; R.KDAAEHTLTAYK.A; K.DAAEHTLTAYK.A; K.AAQDIANSELAPTHPIR.L; K.DSTLIMQLLR.D

PFF Mascot score: **[354]** Sequence coverage %: **[22]**

Matched peptides No.: **[6]**

Calculated Mr: **29997** Calculated *p*I: **4.68**

Annotated PFF **spectra:**

Ions score is -10*Log(P), where P is the probability that the observed match is a random event.
Individual ions scores > 35 indicate identity or extensive homology (p<0.05).
Protein scores are derived from ions scores as a non-probabilistic basis for ranking protein hits.

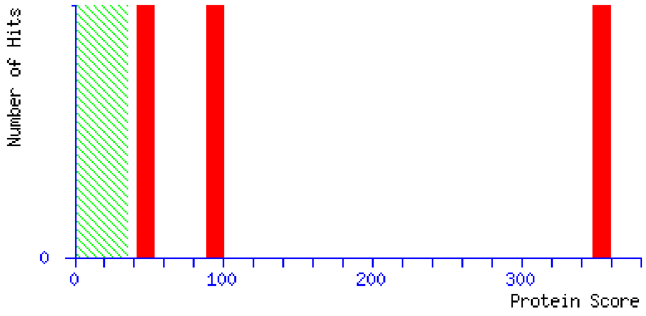

Matched peptide sequences: shown in Bold Red

```
  1 MATPGASSAR DEFVYMAKLA EQAERYEEMV EFMEKVAKAV DKDELTVEER
 51 NLLSVAYKNV IGARRASWRI ISSIEQKEES RGNDDHVSLI RDYRSKIETE
101 LSDICDGILK LLDTILVPAA ASGDSKVFYL KMKGDYHRYL AEFKSGQERK
151 DAAEHTLTAY KAAQDIANSE LAPTHPIRLG LALNFSVFYY EILNSPDRAC
201 NLAKQAFDEA IAELDTLGEE SYKDSTLIMQ LLRDNLTLWA SDMQDDVADD
251 IKEAAPAAAK PADEQQS
```

Spot No.: OL26

NCBI accession No.: **BAH20249.1**

Plant species: ***Arabidopsis thaliana***

Protein name： **AT5G14740, partial**

Peptide sequences: K.YMVFACSDSR.V; R.VCPSHVLDFHPGDAFVVR.N; K.VLAESESSAFEDQCGR.C; R.EAVNVSLANLLTYPFVR.E

PFF Mascot score: **[367]** Sequence coverage %: **[22]**

Matched peptides No.: **[4]**

Calculated Mr: **30867** Calculated *p*I: **5.94**

Annotated PFF **spectra:**

Ions score is -10*Log(P), where P is the probability that the observed match is a random event.
Individual ions scores > 35 indicate identity or extensive homology (p<0.05).
Protein scores are derived from ions scores as a non-probabilistic basis for ranking protein hits.

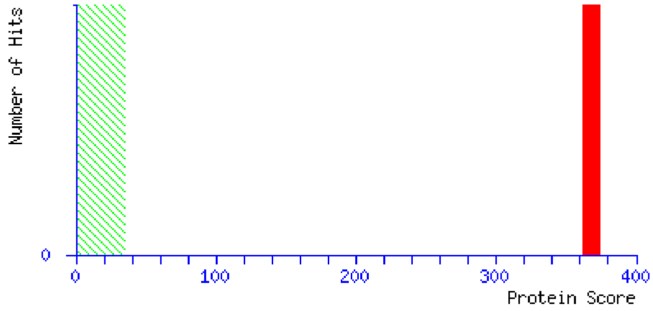

Matched peptide sequences: shown in Bold Red

```
  1 CFRCTCFSHF KLELRRMGNE SYEDAIEALK KLLIEKDDLK DVAAAKVKKI
 51 TAELQAASSS DSKSFDPVER IKEGFVTFKK EKYETNPALY GELAKGQSPK
101 YMVFACSDSR VCPSHVLDFH PGDAFVVRNI ANMVPPFDKV KYAGVGAAIE
151 YAVLHLKVEN IVVIGHSACG GIKGLMSFPL DGNNSTDFIE DWVKICLPAK
201 SKVLAESESS AFEDQCGRCE REAVNVSLAN LLTYPFVREG VVKGTLALKG
251 GYYDFVNGSF ELWELQFGIS PVHSI
```

Spot No.: **OL27**

NCBI accession No.: **AAA03449.1**

Plant species: ***Arabidopsis thaliana***

Protein name： **Cytosolic triose phosphate isomerase**

Peptide sequences: R.AILNESSEFVGDK.V; K.VIACVGETLEER.E; K.VASPAQAQEVHDELR.K; K.VASPAQAQEVHDELRK.W; K.NVSADVAATTR.I

PFF Mascot score: **[363]** Sequence coverage %: **[20]**

Matched peptides No.: **[5]**

Calculated Mr: **27366** Calculated *p*I: **5.24**

Annotated PFF **spectra:**

Ions score is -10*Log(P), where P is the probability that the observed match is a random event.
Individual ions scores > 35 indicate identity or extensive homology ($p<0.05$).
Protein scores are derived from ions scores as a non-probabilistic basis for ranking protein hits.

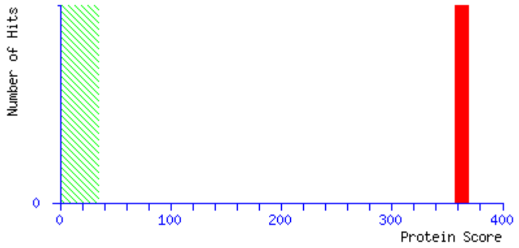

Matched peptide sequences: shown in Bold Red

```
  1 MARKFFVGGN WKCNGTAEEV KKIVNTLNEA QVPSQDVVEV VVSPPYVFLP
 51 LVKSTLRSDF FVAAQNCWVK KGGAFTGEVS AEMLVNLDIP WVILGHSERR
101 AILNESSEFV GDKVAYALAQ GLKVIACVGE TLEEREAGST MDVVAAQTKA
151 IADRVTNWSN VVIAYEPVWA IGTGKVASPA QAQEVHDELR KWLAKNVSAD
201 VAATTRIIYG GSVNGGNCKE LGGQADVDGF LVGGASLKPE FIDIIKAAEV
251 NKSA
```

Spot No.: **OL28**

NCBI accession No.: **AAA03449.1**

Plant species: ***Arabidopsis thaliana***

Protein name：**Cytosolic triose phosphate isomerase**

Peptide sequences: R.SDFFVAAQNCWVK.K; K.VIACVGETLEER.E; K.VASPAQAQEVHDELR.K; K.VASPAQAQEVHDELRK.W; K.NVSADVAATTR.I

PFF Mascot score: **[513]** Sequence coverage %: **[20]**

Matched peptides No.: **[5]**

Calculated Mr: **27366** Calculated *p*I: **5.24**

Annotated PFF **spectra:**

Ions score is -10*Log(P), where P is the probability that the observed match is a random event. Individual ions scores > 35 indicate identity or extensive homology (p<0.05). Protein scores are derived from ions scores as a non-probabilistic basis for ranking protein hits.

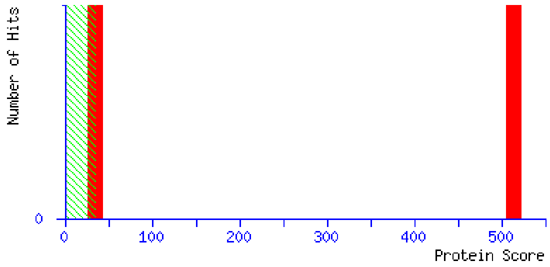

Matched peptide sequences: shown in **Bold Red**

```
  1 MARKFFVGGN WKCNGTAEEV KKIVNTLNEA QVPSQDVVEV VVSPPYVFLP
 51 LVKSTLRSDF FVAAQNCWVK KGGAFTGEVS AEMLVNLDIP WVILGHSERR
101 AILNESSEFV GDKVAYALAQ GLKVIACVGE TLEEREAGST MDVVAAQTKA
151 IADRVTNWSN VVIAYEPVWA IGTGKVASPA QAQEVHDELR KWLAKNVSAD
201 VAATTRIIYG GSVNGGNCKE LGGQADVDGF LVGGASLKPE FIDIIKAAEV
251 NKSA
```

Spot No.: **OL29**

NCBI accession No.: **BAH56801.1**

Plant species: ***Arabidopsis thaliana***

Protein name: **AT3G01500**

Peptide sequences: K.AFDPVETIK.Q; K.YETNPALYGELAK.G; K.YMVFACSDSR.V; R.VCPSHVLDFQPGDAFVVR.N; K.VISELGDSAFEDQCGR.C

PFF Mascot score: **[388]**

Sequence coverage %: **[22]**

Matched peptides No.: **[5]**

Calculated Mr: **31727**

Calculated *p*I: **6.14**

Annotated PFF **spectra:**

Ions score is -10*Log(P), where P is the probability that the observed match is a random event.
Individual ions scores > 35 indicate identity or extensive homology (p<0.05).
Protein scores are derived from ions scores as a non-probabilistic basis for ranking protein hits.

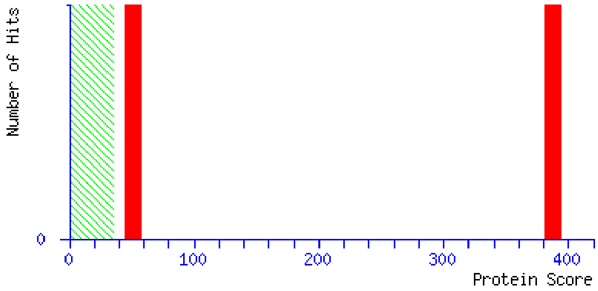

Matched peptide sequences: shown in Bold Red

```
  1 MSTAPLSGFF LTSLSPSQSS LQKLSLRTSS TVACLPPASS SSSSSSSSSS
 51 RSVPTLIRNE PVFAAPAPII APYWSEEMGT EAYDEAIEAL KKLLIEKEEL
101 KTVAAAKVEQ ITAALQTGTS SDKKAFDPVE TIKQGFIKFK KEKYETNPAL
151 YGELAKGQSP KYMVFACSDS RVCPSHVLDF QPGDAFVVRN IANMVPPFDK
201 VKYGGVGAAI EYAVLHLKVE NIVVIGHSAC GGIKGLMSFP LDGNNSTDFI
251 EDWVKICLPA KSKVISELGD SAFEDQCGRC ERVYKHMLDR
```

Spot No.: OL30

NCBI accession No.: **BAH56801.1**

Plant species: ***Arabidopsis thaliana***

Protein name: **AT3G01500**

Peptide sequences: K.AFDPVETIK.Q; K.YETNPALYGELAK.G; K.YMVFACSDSR.V; R.VCPSHVLDFQPGDAFVVR.N; K.VISELGDSAFEDQCGR.C

PFF Mascot score: **[344]** Sequence coverage %: **[22]**

Matched peptides No.: **[5]**

Calculated Mr: **31727** Calculated *p*I: **6.14**

Annotated PFF spectra:

Ions score is -10*Log(P), where P is the probability that the observed match is a random event. Individual ions scores > 35 indicate identity or extensive homology (p<0.05).
Protein scores are derived from ions scores as a non-probabilistic basis for ranking protein hits.

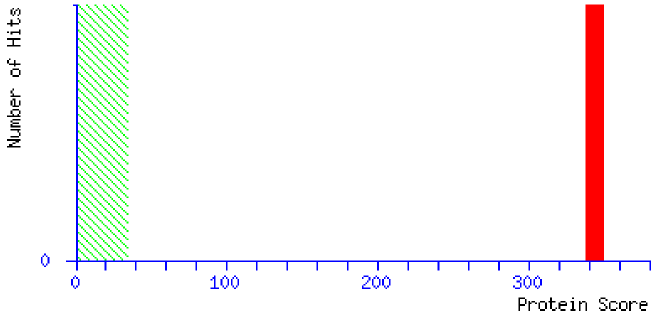

Matched peptide sequences: shown in Bold Red

```
  1 MSTAPLSGFF LTSLSPSQSS LQKLSLRTSS TVACLPPASS SSSSSSSSSS
 51 RSVPTLIRNE PVFAAPAPII APYWSEEMGT EAYDEAIEAL KKLLIEKEEL
101 KTVAAAKVEQ ITAALQTGTS SDKKAFDPVE TIKQGFIKFK KEKYETNPAL
151 YGELAKGQSP KYMVFACSDS RVCPSHVLDF QPGDAFVVRN IANMVPPFDK
201 VKYGGVGAAI EYAVLHLKVE NIVVIGHSAC GGIKGLMSFP LDGNNSTDFI
251 EDWVKICLPA KSKVISELGD SAFEDQCGRC ERVYKHMLDR
```

Spot No.: **OL31**

NCBI accession No.: **BAH56768.1**

Plant species: ***Arabidopsis thaliana***

Protein name： **AT5G54270**

Peptide sequences: R.VDFKEPVWFK.A; R.VDFKEPVWFK.A

PFF Mascot score: **[63]** Sequence coverage %: **[6]**

Matched peptides No.: **[2]**

Calculated Mr: **17583** Calculated *p*I: **4.76**

Annotated PFF spectra:

Ions score is -10*Log(P), where P is the probability that the observed match is a random event.
Individual ions scores > 35 indicate identity or extensive homology (p<0.05).
Protein scores are derived from ions scores as a non-probabilistic basis for ranking protein hits.

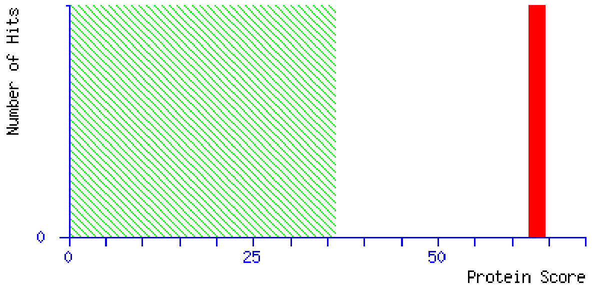

Matched peptide sequences: shown in Bold Red

```
  1 MLGAFGCITP EVLQKWVRVD FKEPVWFKAG SQIFSEGGLD YLGNPNLVHA
 51 QSILAVLGFQ VILMGLVEGF RINGLDGVGE GNDLYPGGQY FDPLGLADDP
101 VTFAELKVKE IKNGRLAMFS MFGFFVQAIV TGKGPLENLL DHLDNPVANN
151 AWAFATKFAP GA
```

Spot No.: **OL32**

NCBI accession No.: **CAA72973.1**

Plant species: ***Arabidopsis thaliana***

Protein name: **Glutathione transferase**

Peptide sequences: K.HVSAWWDDISSR.P

PFF Mascot score: **[68]** Sequence coverage %: **[5]**

Matched peptides No.: **[1]**

Calculated Mr: **24148** Calculated *p*I: **7.03**

Annotated PFF spectra:

Ions score is -10*Log(P), where P is the probability that the observed match is a random event.
Individual ions scores > 35 indicate identity or extensive homology (p<0.05).
Protein scores are derived from ions scores as a non-probabilistic basis for ranking protein hits.

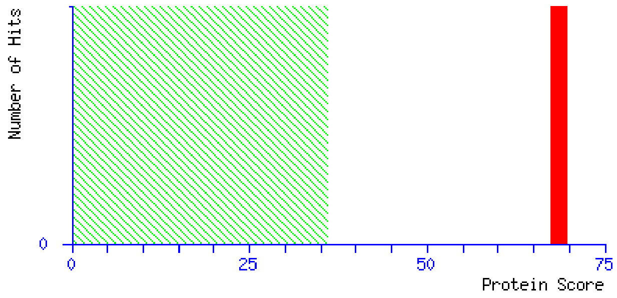

Matched peptide sequences: shown in Bold Red

```
  1 MVLKVYGPHF ASPKRALVTL IEKGVAFETI PVDLMKGEHK QPAYLALQPF
 51 GTVPAVVDGD YKIFESRAVM RYVAEKYRSQ GPDLLGKTVE DRGQVEQWLD
101 VEATTYHPPL LNLTLHIMFA SVMGFPSDEK LIKESEEKLA GVLDVYKAQR
151 AKSKYLAGDF VSLADLAHLP FTDYLVGPIG KAYMIKDRKH VSAWWDDISS
201 RPAWKETVAK YSFPA
```

Spot No.: **OL33**

NCBI accession No.: **BAD94306.1**

Plant species: ***Arabidopsis thaliana***

Protein name：**ferretin 1 precursor, partial**

Peptide sequences: K.ISDYITQLR.M

PFF Mascot score: **[78]** Sequence coverage %: **[18]**

Matched peptides No.: **[1]**

Calculated Mr: **5787** Calculated *p*I: **4.74**

Annotated PFF **spectra:**

Ions score is -10*Log(P), where P is the probability that the observed match is a random event.
Individual ions scores > 35 indicate identity or extensive homology (p<0.05).
Protein scores are derived from ions scores as a non-probabilistic basis for ranking protein hits.

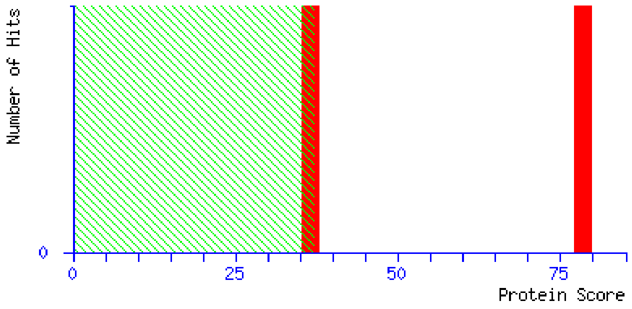

Matched peptide sequences: shown in Bold Red

1 NNDPQLADFV ESEFLGEQIE AIKK**ISDYIT QLR**MIGKGHG VWHFDQMLLN

Spot No.: **OL34**

NCBI accession No.: **AAG30126.1**

Plant species: ***Arabidopsis thaliana***

Protein name：**Glutathione S-transferase**

Peptide sequences: K.VFGHPASTATR.R; R.RVLIALHEK.N; K.VPAFEDGDFK.L; K.VLDVYEHR.L

PFF Mascot score: **[297]** Sequence coverage %: **[18]**

Matched peptides No.: **[4]**

Calculated Mr: **23539** Calculated *p*I: **6.31**

Annotated PFF **spectra:**

Ions score is -10*Log(P), where P is the probability that the observed match is a random event.
Individual ions scores > 35 indicate identity or extensive homology (p<0.05).
Protein scores are derived from ions scores as a non-probabilistic basis for ranking protein hits.

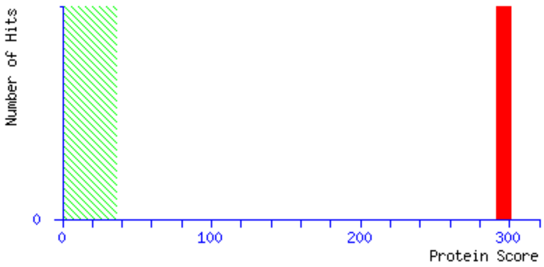

Matched peptide sequences: shown in Bold Red

```
  1 MAGIKVFGHP ASTATRRVLI ALHEKNLDFE FVHIELKDGE HKKEPFIFRN
 51 PFGKVPAFED GDFKLFESRA ITQYIAHFYS DKGNQLVSLG SKDIAGIAMG
101 IEIESHEFDP VGSKLVWEQV LKPLYGMTTD KTVVEEEEAK LAKVLDVYEH
151 RLGESKYLAS DKFTLVDLHT IPVIQYLLGT PTKKLFAERP HVSAWVADIT
201 SRPSAKKVL
```

Spot No.: **OL35**

NCBI accession No.: **AAM61077.1**

Plant species: ***Arabidopsis thaliana***

Protein name: **Ferretin 1 precursor**

Peptide sequences: K.FFKESSEEER.G; K.ISDYITQLR.M

PFF Mascot score: **[107]** Sequence coverage %: **[7]**

Matched peptides No.: **[2]**

Calculated Mr: **28156** Calculated *p*I: **5.73**

Annotated PFF spectra:

Ions score is -10*Log(P), where P is the probability that the observed match is a random event.
Individual ions scores > 35 indicate identity or extensive homology (p<0.05).
Protein scores are derived from ions scores as a non-probabilistic basis for ranking protein hits.

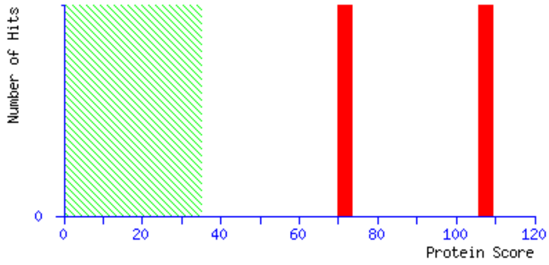

Matched peptide sequences: shown in Bold Red

```
  1 MASNALSSFT AANPALSPKP LLPHGSASPS VSLGFSRKVG GSRAVVVAAA
 51 TVDTNNMPMT GVVFQPLEEV KKADLAIPIT SHASLARQRF ADASEAVINE
101 QINVEYNVSY VYHSMYAYFD RDNVAMKGLA KFFKESSEEE RGHAEKFMEY
151 QNQRGGRVKL HPIVSPISEF EHAEKGDALY AMELALSLEK LTNEKLLNVH
201 KVASENNDPQ LADFVESEFL GEQIEAIKKI SDYITQLRMI GKGHGVWHFD
251 QMLLN
```

Spot No.: **OL36**

NCBI accession No.: **AAA32791.1**

Plant species: ***Arabidopsis thaliana***

Protein name： **Fe-superoxide dismutase, partial**

Peptide sequences: K.QTLEFHWGK.H; K.PSGELLALLER.D; K.TFMTNLVSWEAVSAR.L

PFF Mascot score: **[159]** Sequence coverage %: **[15]**

Matched peptides No.: **[3]**

Calculated Mr: **25409** Calculated *p*I: **6.30**

Annotated PFF spectra:

Ions score is -10*Log(P), where P is the probability that the observed match is a random event.
Individual ions scores > 35 indicate identity or extensive homology (p<0.05).
Protein scores are derived from ions scores as a non-probabilistic basis for ranking protein hits.

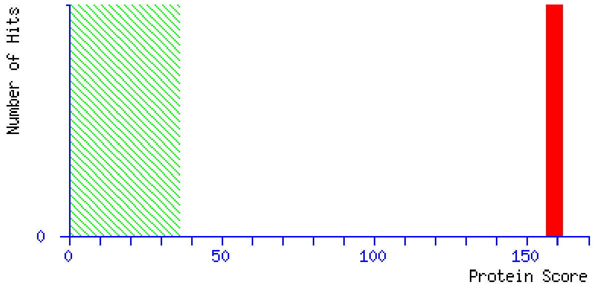

Matched peptide sequences: shown in Bold Red

```
  1 SLHSSLKLEL QLQRMAASSA VTANYVLKPP PFALDALEPH MSKQTLEFHW
 51 GKHHRAYVDN LKKQVLGTEL EGKPLEHIIH STYNNGDLLP AFNNAAQAWN
101 HEFFWESMKP GGGGKPSGEL LALLERDFTS YEKFYEEFNA AAATQFGAGW
151 AWLAYSNEKL KVVKTPNAVN PLVLGSFPLL TIDVWEHAYY LDFQNRRPDY
201 IKTFMTNLVS WEAVSARLEA AKAASA
```

Spot No.: **OL37**

NCBI accession No.: **AAM66134.1**

Plant species: ***Arabidopsis thaliana***

Protein name：**Translationally controlled tumor protein**

Peptide sequences: K.VVDIVDTFR.L; R.LQEQPTYDKK.G

PFF Mascot score: **[115]** Sequence coverage %: **[11]**

Matched peptides No.: **[2]**

Calculated Mr: **18926** Calculated *p*I: **4.52**

Annotated PFF **spectra:**

Ions score is -10*Log(P), where P is the probability that the observed match is a random event.
Individual ions scores > 35 indicate identity or extensive homology (p<0.05).
Protein scores are derived from ions scores as a non-probabilistic basis for ranking protein hits.

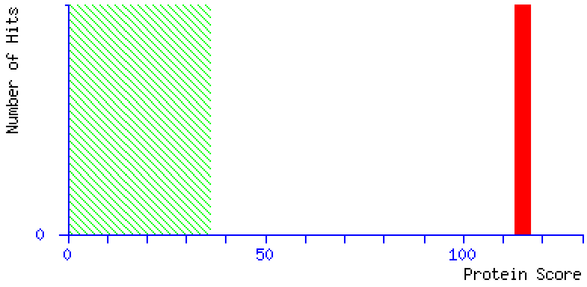

Matched peptide sequences: shown in Bold Red

```
  1 MLVYQDLLTG DELLSDSFPY KEIENGILWE VEGKWVTVGA VDVNIGANPS
 51 AEEGGEDEGV DDSAQKVVDI VDTFRLQEQP TYDKKGFIAY IKKYIKLLTP
101 KLSEEDQAVF KKGIEGATKF LLPRLSDFQF FVGEGMHDDS TLVFAYYKEG
151 STNPTFLYFA HGLKEVKC
```

Spot No.: **OL39**

NCBI accession No.: NP_173813.1

Plant species: ***Arabidopsis thaliana***

Protein name：**MLP-like protein 423**

Peptide sequences: K.TIQVLAGDGNAPGSIR.L; R.LITYGEGSPLVK.I; K.TAHEIDDPHVIKDFAVK.N

PFF Mascot score: **[281]** Sequence coverage %: **[29]**

Matched peptides No.: **[3]**

Calculated Mr: **17044** Calculated *p*I: **5.1**

Annotated PFF **spectra:**

Ions score is -10*Log(P), where P is the probability that the observed match is a random event. Individual ions scores > 35 indicate identity or extensive homology (p<0.05). Protein scores are derived from ions scores as a non-probabilistic basis for ranking protein hits.

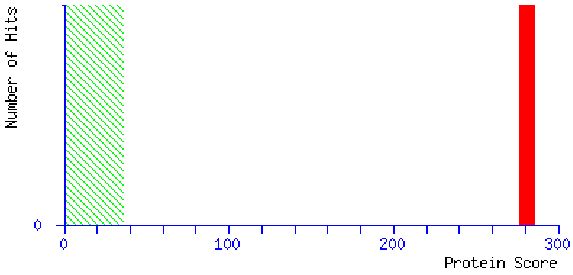

Matched peptide sequences: shown in Bold Red

```
  1 MGLSGVLHVE VEVKSPAEKF WVALGDGINL FPKAFPNDYK TIQVLAGDGN
 51 APGSIRLITY GEGSPLVKIS AERIEAVDLE NKSMSYSIIG GEMLEYYKTF
101 KGTITVIPKN GGSLLKWSGE FEKTAHEIDD PHVIKDFAVK NFKEIDEYLL
151 KQTSA
```

Spot No.: **OL40**

NCBI accession No.: **CAA32701.1**

Plant species: ***Arabidopsis thaliana***

Protein name: **Ribulose bisphosphate carboxylase**

Peptide sequences: R.EHGNTPGYYDGR.Y;

K.LPLFGCTDSAQVLK.E; K.KEYPGAFIR.I

PFF Mascot score: **[261]** Sequence coverage %: **[19]**

Matched peptides No.: **[3]**

Calculated Mr: **20588** Calculated *p*I: **7.59**

Annotated PFF spectra:

Ions score is -10*Log(P), where P is the probability that the observed match is a random event.
Individual ions scores > 35 indicate identity or extensive homology (p<0.05).
Protein scores are derived from ions scores as a non-probabilistic basis for ranking protein hits.

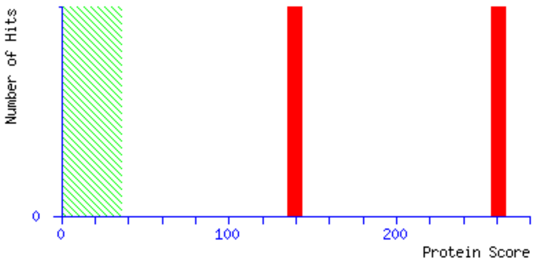

Matched peptide sequences: shown in **Bold Red**

```
  1 MASSMLSSTA VVTSPAQATM VAPFTGLKSS ASFPVTRKAN NDITSITSNG
 51 GRVSCMKVWP PIGKKKFETL SYLPDLSDVE LAKEVDYLLR NKWIPCVEFE
101 LEHGFVYREH GNTPGYYDGR YWTMWKLPLF GCTDSAQVLK EVEECKKEYP
151 GAFIRIIGFD NTRQVQCISF IAYKPPSFTE A
```

Spot No.: **OL41**

NCBI accession No.: **CAA32701.1**

Plant species: ***Arabidopsis thaliana***

Protein name： **Ribulose bisphosphate carboxylase**

Peptide sequences: R.EHGNTPGYYDGR.Y;

K.LPLFGCTDSAQVLK.E

PFF Mascot score: **[133]**   Sequence coverage %: **[14]**

Matched peptides No.: **[2]**

Calculated Mr: **20588**   Calculated *p*I: **7.59**

Annotated PFF **spectra:**

Ions score is -10*Log(P), where P is the probability that the observed match is a random event.
Individual ions scores > 35 indicate identity or extensive homology (p<0.05).
Protein scores are derived from ions scores as a non-probabilistic basis for ranking protein hits.

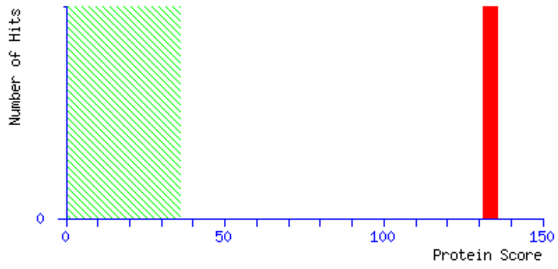

Matched peptide sequences: shown in Bold Red

```
  1 MASSMLSSTA VVTSPAQATM VAPFTGLKSS ASFPVTRKAN NDITSITSNG
 51 GRVSCMKVWP PIGKKKFETL SYLPDLSDVE LAKEVDYLLR NKWIPCVEFE
101 LEHGFVYREH GNTPGYYDGR YWTMWKLPLF GCTDSAQVLK EVEECKKEYP
151 GAFIRIIGFD NTRQVQCISF IAYKPPSFTE A
```

Spot No.: **OL42**

NCBI accession No.: **CAA32701.1**

Plant species: ***Arabidopsis thaliana***

Protein name: **Ribulose bisphosphate carboxylase**

Peptide sequences: R.EHGNTPGYYDGR.Y;

K.LPLFGCTDSAQVLK.E

PFF Mascot score: **[197]** Sequence coverage %: **[14]**

Matched peptides No.: **[2]**

Calculated Mr: **20588** Calculated *p*I: **7.59**

Annotated PFF **spectra:**

Ions score is -10*Log(P), where P is the probability that the observed match is a random event. Individual ions scores > 35 indicate identity or extensive homology (p<0.05).
Protein scores are derived from ions scores as a non-probabilistic basis for ranking protein hits.

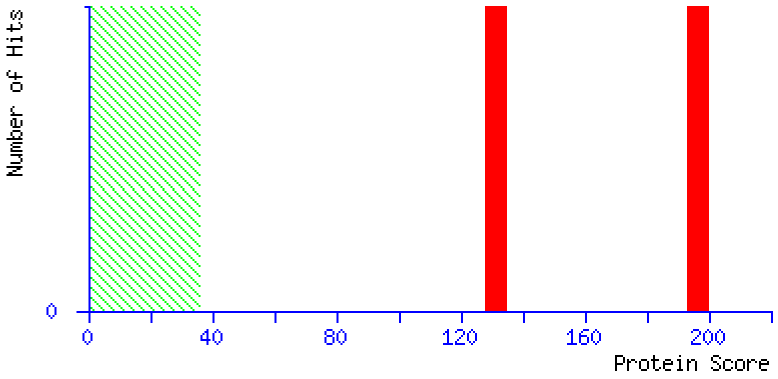

Matched peptide sequences: shown in **Bold Red**

```
  1 MASSMLSSTA VVTSPAQATM VAPFTGLKSS ASFPVTRKAN NDITSITSNG
 51 GRVSCMKVWP PIGKKKFETL SYLPDLSDVE LAKEVDYLLR NKWIPCVEFE
101 LEHGFVYREH GNTPGYYDGR YWTMWKLPLF GCTDSAQVLK EVEECKKEYP
151 GAFIRIIGFD NTRQVQCISF IAYKPPSFTE A
```

Spot No.: **OL43**

NCBI accession No.: **CAA32701.1**

Plant species: ***Arabidopsis thaliana***

Protein name： **Ribulose bisphosphate carboxylase**

Peptide sequences: **R.EHGNTPGYYDGR.Y**

PFF Mascot score: **[68]**

Sequence coverage %: **[6]**

Matched peptides No.: **[1]**

Calculated Mr: **20588**

Calculated *p*I: **7.59**

Annotated PFF **spectra:**

Ions score is -10*Log(P), where P is the probability that the observed match is a random event.
Individual ions scores > 35 indicate identity or extensive homology (p<0.05).
Protein scores are derived from ions scores as a non-probabilistic basis for ranking protein hits.

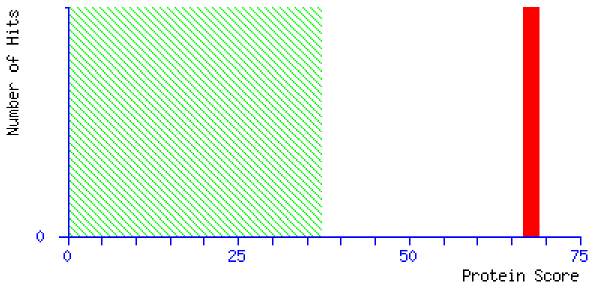

Matched peptide sequences: shown in Bold Red

1 MASSMLSSTA VVTSPAQATM VAPFTGLKSS ASFPVTRKAN NDITSITSNG
51 GRVSCMKVWP PIGKKKFETL SYLPDLSDVE LAKEVDYLLR NKWIPCVEFE
101 LEHGFVYR**EH GNTPGYYDGR** YWTMWKLPLF GCTDSAQVLK EVEECKKEYP
151 GAFIRIIGFD NTRQVQCISF IAYKPPSFTE A

Spot No.: **OL44**

NCBI accession No.: **CAA31948.1**

Plant species: ***Arabidopsis thaliana***

Protein name: **Ribulose bisphosphate carboxylase**

Peptide sequences: R.EHGNSPGYYDGR.Y;

K.LPLFGCTDSAQVLK.E; K.KEYPNAFIR.I; K.EYPNAFIR.I

PFF Mascot score: **[303]** Sequence coverage %: **[19]**

Matched peptides No.: **[4]**

Calculated Mr: **20733** Calculated *p*I: **7.59**

Annotated PFF **spectra:**

Ions score is -10*Log(P), where P is the probability that the observed match is a random event. Individual ions scores > 35 indicate identity or extensive homology (p<0.05).
Protein scores are derived from ions scores as a non-probabilistic basis for ranking protein hits.

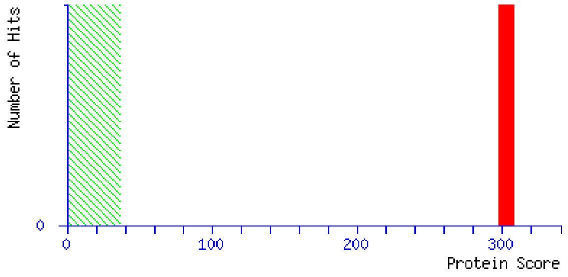

Matched peptide sequences: shown in Bold Red

```
  1 MASSMLSSAT MVASPAQATM VAPFNGLKSS AAFPATRKAN NDITSITSNG
 51 GRVNCMQMQV WPPIGKKKFE TLSYLPDLTD SELAKEVDYL IRNKWIPCVE
101 FELEHGFVYR EHGNSPGYYD GRYWTMWKLP LFGCTDSAQV LKEVEECKKE
151 YPNAFIRIIG FDNTRQVQCI SFVAYKPPSF TG
```

Spot No.: **OL45**

NCBI accession No.: **CAA31948.1**

Plant species: ***Arabidopsis thaliana***

Protein name：**ribulose bisphosphate carboxylase**

Peptide sequences: R.EHGNSPGYYDGR.Y;

K.LPLFGCTDSAQVLK.E; K.KEYPNAFIR.I; K.EYPNAFIR.I

PFF Mascot score: **[324]** Sequence coverage %: **[19]**

Matched peptides No.: **[4]**

Calculated Mr: **20733** Calculated *p*I: **7.59**

Annotated PFF **spectra:**

Ions score is -10*Log(P), where P is the probability that the observed match is a random event.
Individual ions scores > 35 indicate identity or extensive homology (p<0.05).
Protein scores are derived from ions scores as a non-probabilistic basis for ranking protein hits.

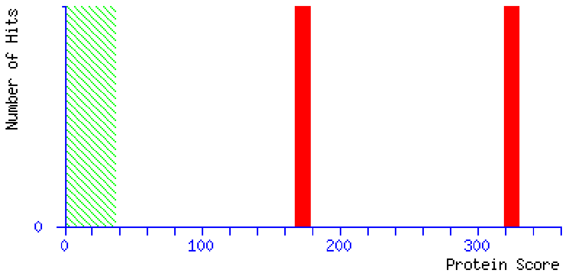

Matched peptide sequences: shown in Bold Red

```
  1 MASSMLSSAT MVASPAQATM VAPFNGLKSS AAFPATRKAN NDITSITSNG
 51 GRVNCMQMQV WPPIGKKKFE TLSYLPDLTD SELAKEVDYL IRNKWIPCVE
101 FELEHGFVYR EHGNSPGYYD GRYWTMWKLP LFGCTDSAQV LKEVEECKKE
151 YPNAFIRIIG FDNTRQVQCI SFVAYKPPSF TG
```

Spot No.:OR1

NCBI accession No.: **NP_199996.1**

Plant species: ***Arabidopsis thaliana***

Protein name: ***PfkB-like carbohydrate kinase family protein***

Peptide sequences: K.KAPGGAPANVAVGVSR.L; K.IFHYGSISLIEEPCR.S; K.AAGSLLSYDPNLR.L; R.LPLWPSEEAAR.K

PFF Mascot score: **[343]** Sequence coverage %: **[16]**

Matched peptides No.: **[4]**

Calculated Mr: **37233** Calculated *p*I: **4.99**

Annotated PFF **spectra:**

Ions score is -10*Log(P), where P is the probability that the observed match is a random event.
Individual ions scores > 35 indicate identity or extensive homology (p<0.05).
Protein scores are derived from ions scores as a non-probabilistic basis for ranking protein hits.

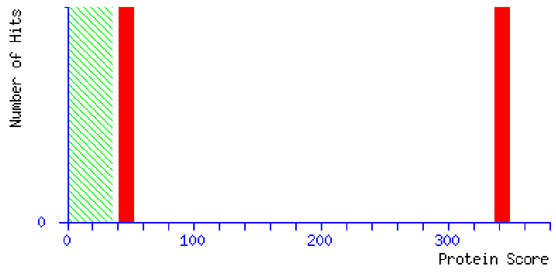

Matched peptides shown in bold red**.**

```
  1 MGEDAISGNL KNLTIDTRDS ETLVVCFGEM LIDFVPTVGG VSLAEAPAFK
 51 KAPGGAPANV AVGVSRLGGS SAFIGKVGDD EFGRMLADIL RLNNVDNSGM
101 RFDHNARTAL AFVTLRGDGE REFLFFRHPS ADMLLLESEL DKNLIQKAKI
151 FHYGSISLIE EPCRSTQLVA MKIAKAAGSL LSYDPNLRLP LWPSEEAARK
201 EIMSIWNLAD VIKISEDEIT FLTGGDDPYD DDVVLQKLFH PNLKLLVVSE
251 GPNGCRYYTQ EFKGRVGGVK VKPVDTTGAG DAFVSGLLNS LASDLTLLKD
301 EKKLREALLF ANACGAITVT ERGAIPAMPS MDAVQDLLSS TRS
```

Spot No.:OR2

NCBI accession No.: **AAC27484.1**

Plant species: ***Arabidopsis thaliana***

Protein name: **ACC oxidase**

Peptide sequences: K.GLDNLETEVEDVDWESTFYVR.H; R.HLPQSNLNDISDVSDEYR.T; R.VVTQQEGNR.M

PFF Mascot score: **[254]** Sequence coverage %: **[15]**

Matched peptides No.: **[3]**

Calculated Mr: **36374** Calculated *p*I: **4.97**

Annotated PFF spectra:

Ions score is -10*Log(P), where P is the probability that the observed match is a random event.
Individual ions scores > 35 indicate identity or extensive homology (p<0.05).
Protein scores are derived from ions scores as a non-probabilistic basis for ranking protein hits.

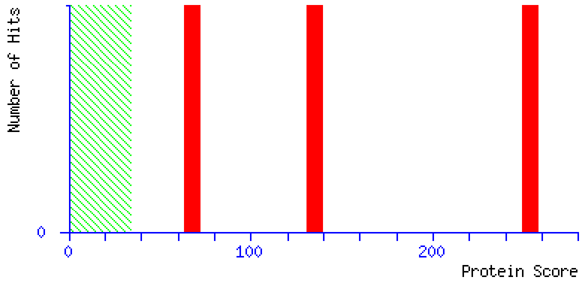

Matched peptides shown in bold red.

```
  1 MEKNMKFPVV DLSKLNGEER DQTMALINEA CENWGFFEIV NHGLPHDLMD
 51 KIEKMTKDHY KTCQEQKFND MLKSKGLDNL ETEVEDVDWE STFYVRHLPQ
101 SNLNDISDVS DEYRTAMKDF GKRLENLAED LLDLLCENLG LEKGYLKKVF
151 HGTKGPTFGT KVSNYPPCPK PEMIKGLRAH TDAGGIILLF QDDKVSGLQL
201 LKDGDWIDVP PLNHSIVINL GDQLEVITNG KYKSVLHRVV TQQEGNRMSV
251 ASFYNPGSDA DISPATSLVE KDSEYPSFVF DDYMKLYAGV KFQPKEPRFA
301 AMKNASAVTE LNPTAAVETF
```

Spot No.:OR4

NCBI accession No.: **AAC14026.1**

Plant species: ***Arabidopsis thaliana***

Protein name: **Chaperonin 10**

Peptide sequences: K.YTSIKPLGDR.V; K.DGSNYIALR.A

PFF Mascot score: **[111]** Sequence coverage %: **[7]**

Matched peptides No.: **[2]**

Calculated Mr: **26912** Calculated *p*I: **8.86**

Annotated PFF spectra:

Ions score is -10*Log(P), where P is the probability that the observed match is a random event.
Individual ions scores > 35 indicate identity or extensive homology (p<0.05).
Protein scores are derived from ions scores as a non-probabilistic basis for ranking protein hits.

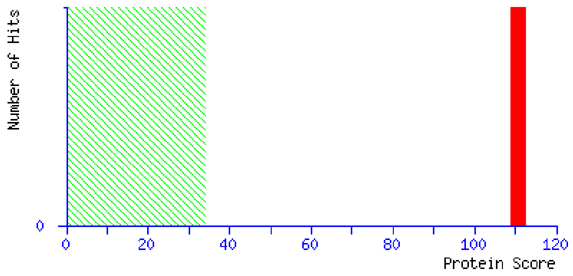

Matched peptides shown in bold red.

```
  1 MAATQLTASP VTMSARSLAS LDGLRASSVK VSSLKPGTLR QSQFRRLVVK
 51 AASVVAPKYT SIKPLGDRVL VKIKEAEEKT LGGILTFHST AQSKPQGGEV
101 VAVGEGRTIG KNKIDITVPT GAQIIYSKYA GTEVEFNDVK HLILKEDDIV
151 GILETEDIKD LKPLNDRVFI KVAEAEEKTA GGLLLTETTK EKPSIGTVIA
201 VGPGSLDEEG KITPLPVSTG STVLYSKYAG NDFKGKDGSN YIALRASDVM
251 AILS
```

Spot No.:OR5

NCBI accession No.: **NP_173387.1**

Plant species: ***Arabidopsis thaliana***

Protein name: **Dehydroascorbate reductase**

Peptide sequences: K.AAVGAPDHLGDCPFSQR.A; K.SKDSNDGSEHALLVELEALENHLK.S; K.SHDGPFIAGER.V; K.SWSVPESFPHVHNYMK.T + Oxidation (M); K.YVISGWAPK.V

PFF Mascot score: **[356]** Sequence coverage %: **[36]**

Matched peptides No.: **[5]**

Calculated Mr: **23740** Calculated *p*I: **5.56**

Annotated PFF **spectra:**

Ions score is -10*Log(P), where P is the probability that the observed match is a random event.
Individual ions scores > 35 indicate identity or extensive homology (p<0.05).
Protein scores are derived from ions scores as a non-probabilistic basis for ranking protein hits.

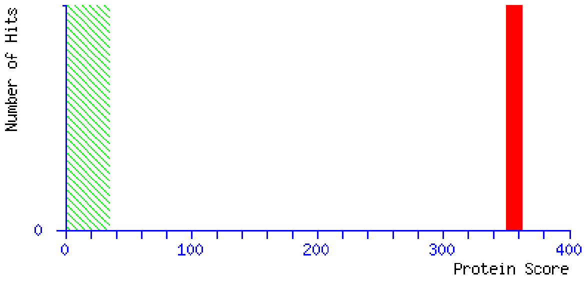

Matched peptides shown in bold red**.**

1 MALEICVKAA VGAPDHLGDC PFSQRALLTL EEKSLTYKIH LINLSDKPQW
51 FLDISPQGKV PVLKIDDKWV TDSDVIVGIL EEKYPDPPLK TPAEFASVGS
101 NIFGTFGTFL KSKDSNDGSE HALLVELEAL ENHLKSHDGP FIAGERVSAV
151 DLSLAPKLYH LQVALGHFKS WSVPESFPHV HNYMKTLFSL DSFEKTKTEE
201 KYVISGWAPK VNP

Spot No.:OR6

NCBI accession No.: **NP_180643.1**

Plant species: ***Arabidopsis thaliana***

Protein name:**Glutathione S-transferase PHI 9**

Peptide sequences: K.VYGPHFASPK.R;

K.GVAFETIPVDLMK.G; K.GVAFETIPVDLMK.G + Oxidation (M);

K.LAGVLDVYEAHLSK.S; R.KHVSAWWDDISSR.P;

K.HVSAWWDDISSR.P

PFF Mascot score: **[432]** Sequence coverage %: **[23]**

Matched peptides No.: **[6]**

Calculated Mr: **24131** Calculated *p*I: **6.17**

Annotated PFF spectra:

Ions score is -10*Log(P), where P is the probability that the observed match is a random event.
Individual ions scores > 35 indicate identity or extensive homology (p<0.05).
Protein scores are derived from ions scores as a non-probabilistic basis for ranking protein hits.

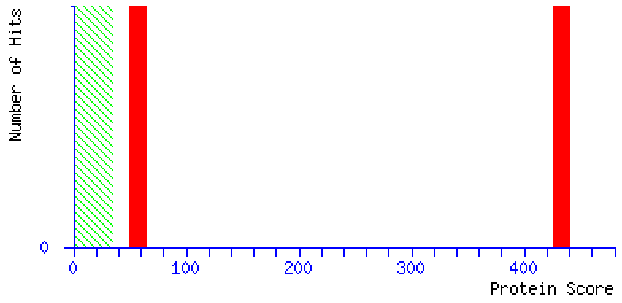

Matched peptides shown in bold red.

```
  1 MVLKVYGPHF ASPKRALVTL IEKGVAFETI PVDLMKGEHK QPAYLALQPF
 51 GTVPAVVDGD YKIFESRAVM RYVAEKYRSQ GPDLLGKTVE DRGQVEQWLD
101 VEATTYHPPL LNLTLHIMFA SVMGFPSDEK LIKESEEKLA GVLDVYEAHL
151 SKSKYLAGDF VSLADLAHLP FTDYLVGPIG KAYMIKDRKH VSAWWDDISS
201 RPAWKETVAK YSFPA
```

Spot No.:OR7

NCBI accession No.: **AAC04902.1**

Plant species: ***Arabidopsis thaliana***

Protein name: **Mitochondrial chaperonin (HSP60)**

Peptide sequences: R.NVIIEQSWGAPK.V; K.QVANATNDVAGDGTTCATVLTR.A; K.LAVDTVVTNLQSR.A; K.LLEQDNPDLGYDAAK.G

PFF Mascot score: **[260]** Sequence coverage %: **[11]**

Matched peptides No.: **[4]**

Calculated Mr: **55561** Calculated *p*I: **5.30**

Annotated PFF spectra:

Ions score is -10*Log(P), where P is the probability that the observed match is a random event.
Individual ions scores > 35 indicate identity or extensive homology (p<0.05).
Protein scores are derived from ions scores as a non-probabilistic basis for ranking protein hits.

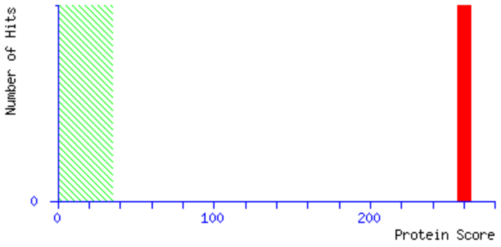

Matched peptides shown in bold red.

1 MGPKGR**NVII** **EQSWGAPK**VT KDGVTVAKSI EFKDRIKNVG ASLVK**QVANA**
51 **TNDVAGDGTT** **CATVLTR**AIF TEGCKSVAAG MNAMDLRRGI K**LAVDTVVTN**
101 **LQSR**ARMIST SEEIAQVGTI SANGDREIGE LIAKAMETVG KEGVITIQDG
151 KTLFNELEVV EGMKIDRGYI SPYFITNPKT QKCELEDPLI LIHEKKISNI
201 NAMVKVLELA LKKQRPLLIV AEDVESDALA TLILNKLRAN IKVCAVKAPG
251 FGENRKANLH DLAALTGAQV ITEELGMNLD NIDLSMFGNC KKVTVSKDDT
301 VVLDGAGDKQ AIGERCEQIR SMVEASTSDY DKEKLQERLA KLSGGVAVLK
351 IGGASETEVS EKKDRVTDAL NATKAAVEEG IVPGGGVALL YASKELEKLS
401 TANFDQKIGV QIIQNALKTP VYTIASNAGV EGAVVVGK**LL** **EQDNPDLGYD**
451 **AAK**GEYVDMI KAGIIDPLKV IRTALVDAAS VSSLLTTTEA VVTEIPTKEV
501 ASPGMGGGGM GGMGGMGGMG GMGF

Spot No.:OR8

NCBI accession No.: **OAP06349.1**

Plant species: ***Arabidopsis thaliana***

Protein name: **TSA1（tryptophan synthase beta subunit）**

Peptide sequences: R.AVGVQGLVVPDVPLEETEMLR.K; K.EALNNDIELVLLTTPTTPTER.M

PFF Mascot score: **[219]** Sequence coverage %: **[13]**

Matched peptides No.: **[2]**

Calculated Mr: **33362** Calculated *p*I: **7.68**

Annotated PFF **spectra:**

Ions score is -10*Log(P), where P is the probability that the observed match is a random event.
Individual ions scores > 35 indicate identity or extensive homology ($p<0.05$).
Protein scores are derived from ions scores as a non-probabilistic basis for ranking protein hits.

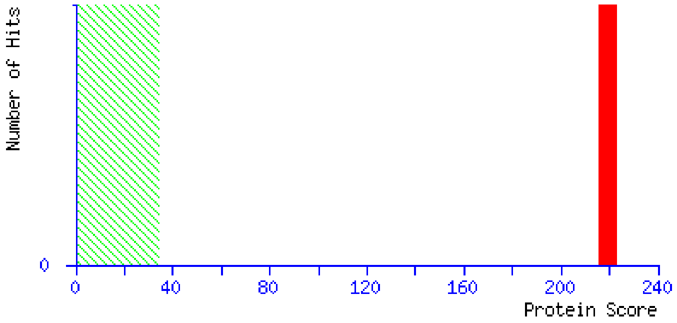

Matched peptides shown in bold red.

```
  1 MAIAFKSGVF FLQSPKSQIG FRHSSPPDSS LSFKRFTPMA SLSTSSPTLG
 51 LADTFTQLKK QGKVPFIPYI TAGDPDLSTT AEALKVLDAC GSDIIELGVP
101 YSDPLADGPV IQAAATRSLE RGTNLDSILE MLDKVVPQIS CPISLFTYYN
151 PILKRGLGKF MSSIRAVGVQ GLVVPDVPLE ETEMLRKEAL NNDIELVLLT
201 TPTTPTERMK RIVDASEGFI YLVSSIGVTG ARSSVSGKVQ SLLKDIKEAT
251 DKPVAVGFGI SKPEHVKQIA GWGADGVIVG SAMVKLLGDA KSPTEGLKEL
301 EKLTKSLKSA LL
```

Spot No.:OR9

NCBI accession No.: **AAN31820.1**

Plant species: ***Arabidopsis thaliana***

Protein name: **Putative cysteine proteinase AALP**

Peptide sequences: K.DWREDGIVSPVK.D; K.AYPYTGKDETCK.F; K.NSWGADWGDK.G

PFF Mascot score: **[161]** Sequence coverage %: **[9]**

Matched peptides No.: **[3]**

Calculated Mr: **39495** Calculated *p*I: **6.26**

Annotated PFF **spectra:** Ions score is -10*Log(P), where P is the probability that the observed match is a random event.
Individual ions scores > 35 indicate identity or extensive homology (p<0.05).
Protein scores are derived from ions scores as a non-probabilistic basis for ranking protein hits.

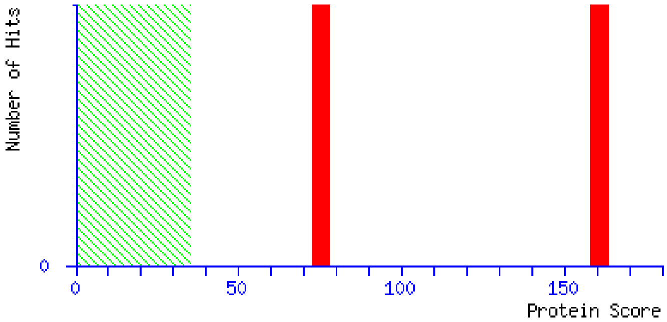

Matched peptides shown in bold red.

```
  1 MSAKTILSSV VLVVLFAASA AANIGFDESN PIRMVSDGLR EVEESVSQIL
 51 GQSRHVLSFA RFTHRYGKKY QNVEEMKLRF SIFKENLDLI RSTNKKGLSY
101 KLGVNQFADL TWQEFQRTKL GAAQNCSATL KGSHKVTEAA LPETKDWRED
151 GIVSPVKDQG GCGSCWTFST TGALEAAYHQ AFGKGISLSE QQLVDCAGAF
201 NNYGCNGGLP SQAFEYIKSN GGLDTEKAYP YTGKDETCKF SAENVGVQVL
251 NSVNITLGAE DELKHAVGLV RPVSIAFEVI HSFRLYKSGV YTDSHCGSTP
301 MDVNHAVLAV GYGVEDGVPY WLIKNSWGAD WGDKGYFKME MGKNMCGIAT
351 CASYPVVA
```

Spot No.:OR10

NCBI accession No.: **AAL07227.1**

Plant species: ***Arabidopsis thaliana***

Protein name: **Putative lactoylglutathione lyase**

Peptide sequences: K.DPDGYTFELIQR.G; R.GPTPEPFCQVMLR.V; R.GPTPEPFCQVMLR.V + Oxidation (M); R.EAGPLPGLGTK.I; K.IVSFLDPDGWK.T

PFF Mascot score: **[292]** Sequence coverage %: **[16]**

Matched peptides No.: **[5]**

Calculated Mr: **31995** Calculated *p*I: **5.11**

Annotated PFF **spectra:**

Ions score is -10*Log(P), where P is the probability that the observed match is a random event.
Individual ions scores > 35 indicate identity or extensive homology (p<0.05).
Protein scores are derived from ions scores as a non-probabilistic basis for ranking protein hits.

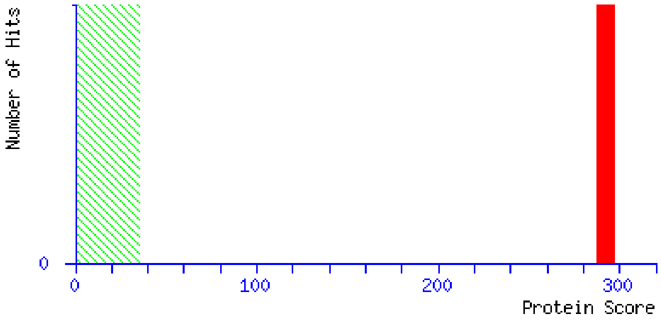

Matched peptides shown in bold red.

```
  1 MAEASDLLEW PKKDNRRFLH VVYRVGDLDR TIEFYTEVFG MKLLWKRDIP
 51 EEKYSNAFLG FGPETSNFVV ELTYNYGVSS YDIGTGFGHF AISTQDVSKL
101 VENVRAKGGN VTREPGPVKG GGSVIAFVKD PDGYTFELIQ RGPTPEPFCQ
151 VMLRVGDLDR AIKFYEKALG MRLLRKIERP EYKYTIGMMG YAEEYESIVL
201 ELTYNYDVTE YTKGNAYAQI AIGTDDVYKS GEVIKIVNQE LGGKITREAG
251 PLPGLGTKIV SFLDPDGWKT VLVDNKDFLK ELE
```

Spot No.:OR11

NCBI accession No.: **AAB17995.1**

Plant species: ***Arabidopsis thaliana***

Protein name: **Glyoxalase II**

Peptide sequences: K.VSIGDIYLEVR.A; R.ATPGHTAGCVTYVTGEGADQPQPR.M; R.MAFTGDAVLIR.G; R.MAFTGDAVLIR.G + Oxidation (M); K.GFEVSTVGEEMQHNPR.L

PFF Mascot score: **[455]** Sequence coverage %: **[24]**

Matched peptides No.: **[5]**

Calculated Mr: **27941** Calculated *p*I: **5.58**

Annotated PFF **spectra:**

Ions score is -10*Log(P), where P is the probability that the observed match is a random event.
Individual ions scores > 35 indicate identity or extensive homology (p<0.05).
Protein scores are derived from ions scores as a non-probabilistic basis for ranking protein hits.

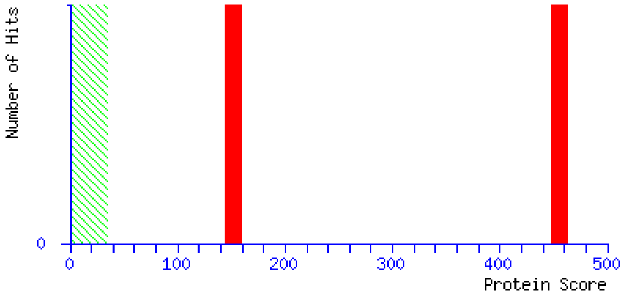

Matched peptides shown in bold red.

```
  1 MGSSSSFSSS SSKLLFRQLF ENESSTFTYL LADVSHPDKP ALLIDPVDKT
 51 VDRDLKLIDE LGLKLIYAMN THVHADHVTG TGLLKTKLPG VKSVISKASG
101 SKADLFLEPG DKVSIGDIYL EVRATPGHTA GCVTYVTGEG ADQPQPRMAF
151 TGDAVLIRGC GRTDFQGGSS DQLYESVHSQ IFSLPKDTLI YPAHDYKGFE
201 VSTVGEEMQH NPRLTKDKET FKTIMSNLNL SYPKMIDVAV PANMVCGLQD
251 VPSQAN
```

Spot No.:OR12

NCBI accession No.: **NP_565203.1**

Plant species: ***Arabidopsis thaliana***

Protein name: **Mercaptopyruvate sulfurtransferase 1**

Peptide sequences: K.ILDASWYMPDEQR.N; R.NPIQEYQVAHIPR.A; R.ALFFDLDGISDR.K; K.VWVLDGGLPR.W

PFF Mascot score: **[343]** Sequence coverage %: **[12]**

Matched peptides No.: **[4]**

Calculated Mr: **42152** Calculated *p*I: **5.95**

Annotated PFF spectra:

Ions score is -10*Log(P), where P is the probability that the observed match is a random event.
Individual ions scores > 35 indicate identity or extensive homology (p<0.05).
Protein scores are derived from ions scores as a non-probabilistic basis for ranking protein hits.

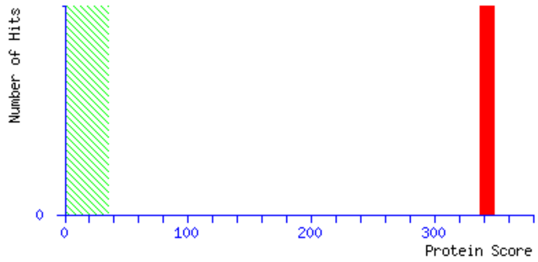

Matched peptides shown in bold red.

```
  1 MASTLFSRTF LAASHRLITP SLPQKIFNPA TFLSRSLHSQ LGSASTAYKS
 51 TTWARRAMAS TGVETKAGYS TSSVSTSEPV VSVDWLHANL REPDLKILDA
101 SWYMPDEQRN PIQEYQVAHI PRALFFDLDG ISDRKTSLPH MLPTEEAFAA
151 GCSALGIDNK DEVVVYDGKG IFSAARVWWM FRVFGHEKVW VLDGGLPRWR
201 ASGYDVESSA SGDAILKASA ASEAIEKIYQ GQTVSPITFQ TKFQPHLVWT
251 LDQVKNNMED PTYQHIDARS KARFDGTAPE PRKGIRSGHI PGSKCIPFPQ
301 MFDSCNTLLP AEELKKRFDQ EDISLDKPIM ASCGTGVTAC ILAMGLHRLG
351 KTDVPIYDGS WTEWATQPDL PIESVESSS
```

Spot No.:OR13

NCBI accession No.: **NP_199898.1**

Plant species: ***Arabidopsis thaliana***

Protein name: **Transketolase family protein**

Peptide sequences: K.VFVMGEEVGQYQGAYK.I; K.VLAPYSAEDAR.G; K.LAEEGISAEVINLR.S; R.IAGADVPMPYAANLER.L; R.LALPQIEDIVR.A

PFF Mascot score: **[409]** Sequence coverage %: **[18]**

Matched peptides No.: **[5]**

Calculated Mr: **39436** Calculated *p*I: **5.67**

Annotated PFF **spectra:**

Ions score is -10*Log(P), where P is the probability that the observed match is a random event.
Individual ions scores > 35 indicate identity or extensive homology (p<0.05).
Protein scores are derived from ions scores as a non-probabilistic basis for ranking protein hits.

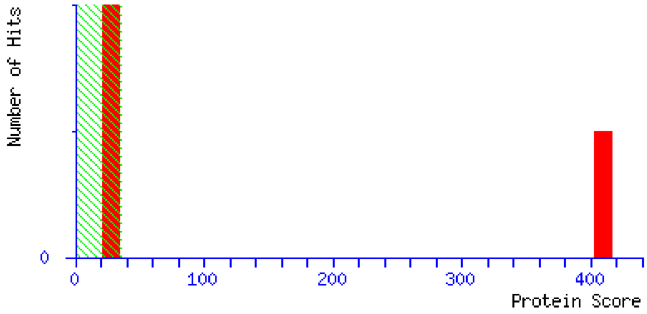

Matched peptides shown in bold red.

```
  1 MLGILRQRAI DGASTLRRTR FALVSARSYA AGAKEMTVRD ALNSAIDEEM
 51 SADPKVFVMG EEVGQYQGAY KITKGLLEKY GPERVYDTPI TEAGFTGIGV
101 GAAYAGLKPV VEFMTFNFSM QAIDHIINSA AKSNYMSAGQ INVPIVFRGP
151 NGAAAGVGAQ HSQCYAAWYA SVPGLKVLAP YSAEDARGLL KAAIRDPDPV
201 VFLENELLYG ESFPISEEAL DSSFCLPIGK AKIEREGKDV TIVTFSKMVG
251 FALKAAEKLA EEGISAEVIN LRSIRPLDRA TINASVRKTS RLVTVEEGFP
301 QHGVCAEICA SVVEESFSYL DAPVERIAGA DVPMPYAANL ERLALPQIED
351 IVRASKRACY RSK
```

Spot No.:OR14

NCBI accession No.: **NP_850976.1**

Plant species: ***Arabidopsis thaliana***

Protein name: **MLP-like protein 34**

Peptide sequences: K.TEAPSLVGKLETEVEIK.A;

R.IEAVEPEKNLITFR.V; R.VIEGDLMKEYK.S;

R.VIEGDLMKEYK.S + Oxidation (M); K.TTETLETEVEIK.A;

K.EIDEHLLAEE.-

PFF Mascot score: **[329]** Sequence coverage %: **[20]**

Matched peptides No.: **[6]**

Calculated Mr: **35719** Calculated *p*I: **5.14**

Annotated PFF **spectra:**

Ions score is -10*Log(P), where P is the probability that the observed match is a random event.
Individual ions scores > 35 indicate identity or extensive homology (p<0.05).
Protein scores are derived from ions scores as a non-probabilistic basis for ranking protein hits.

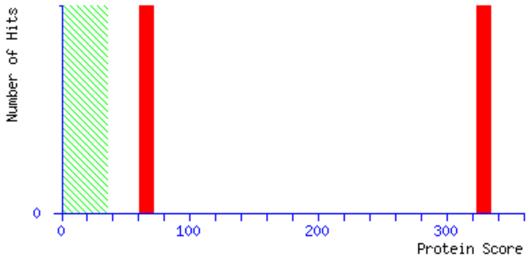

Matched peptides shown in bold red**.**

```
  1 MAKTEAPSLV GKLETEVEIK ASAGQFHHMF AGKPHHVSKA SPGNIQSCDL
 51 HEGDWGTVGS IVFWNYVHDG EAKVAKERIE AVEPEKNLIT FRVIEGDLMK
101 EYKSFLITIQ VTPKHGGPGS IVHWHLEYEK ISDEVAHPET LLQFCVEVSQ
151 EIDEHLLSEE EEVKTTETLE TEVEIKASAE KFHHMFAGKP HHVSKATPGN
201 IQSCDLHEGD WGTVGSIVFW NYVHDGEAKV AKERIEAVDP EKNLITFRVI
251 EGDLMKEYKS FVITIQVTPK HGGSGSVVHW HFEYEKINEE VAHPETLLQF
301 AVEVSKEIDE HLLAEE
```

Spot No.:OR15

NCBI accession No.: **NP_565107.1**

Plant species: ***Arabidopsis thaliana***

Protein name: **NmrA-like negative transcriptional regulator**

Peptide sequences: K.AGHSTFALVR.E;

K.DLGVTILHGDLNDHESLVK.A; R.FLPSEFGVDVDR.T;

K.AVINKEEDIAAYTIK.A

PFF Mascot score: **[336]** Sequence coverage %: **[18]**

Matched peptides No.: **[4]**

Calculated Mr: **33733** Calculated *p*I: **5.66**

Annotated PFF spectra:

Ions score is -10*Log(P), where P is the probability that the observed match is a random event.
Individual ions scores > 35 indicate identity or extensive homology (p<0.05).
Protein scores are derived from ions scores as a non-probabilistic basis for ranking protein hits.

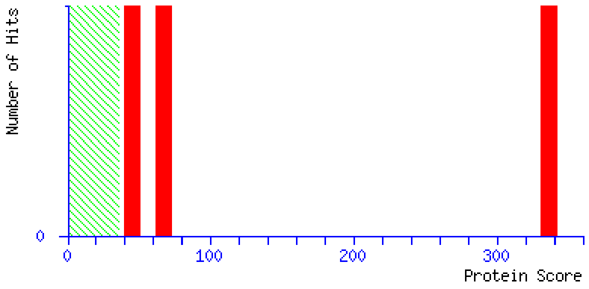

Matched peptides shown in bold red.

```
  1 MATEKSKILV IGGTGYIGKF LVEASAKAGH STFALVREAT LSDPVKGKTV
 51 QSFKDLGVTI LHGDLNDHES LVKAIKQVDV VISTVGSMQI LDQTKIISAI
101 KEAGNVKRFL PSEFGVDVDR TSAVEPAKSA FAGKIQIRRT IEAEGIPYTY
151 AVTGCFGGYY LPTLVQFEPG LTSPPRDKVT ILGDGNAKAV INKEEDIAAY
201 TIKAVDDPRT LNKILYIKPS NNTLSMNEIV TLWEKKIGKS LEKTHLPEEQ
251 LLKSIQESPI PINVVLSINH AVFVNGDTNI SIEPSFGVEA SELYPDVKYT
301 SVDEYLSYFA
```

Spot No.:OR16

NCBI accession No.: **OAP16650.1**

Plant species: ***Arabidopsis thaliana***

Protein name: ***GSTU26***

Peptide sequences: R.FWAEFIDKK.F; R.IIEYVYVLR.K

PFF Mascot score: **[106]** Sequence coverage %: **[8]**

Matched peptides No.: **[2]**

Calculated Mr: **25891** Calculated *p*I: **5.53**

Annotated PFF spectra:

Ions score is -10*Log(P), where P is the probability that the observed match is a random event.
Individual ions scores > 35 indicate identity or extensive homology (p<0.05).
Protein scores are derived from ions scores as a non-probabilistic basis for ranking protein hits.

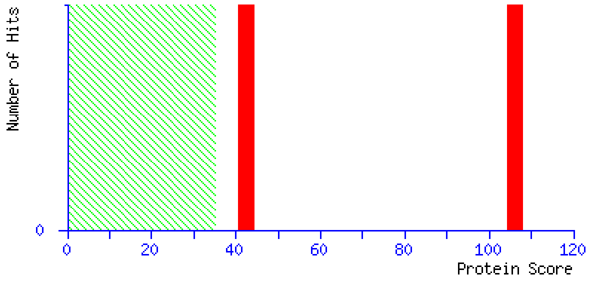

Matched peptides shown in bold red.

```
  1 MANDQVILLD YWPSMFGMRT KMALAEKGVK YEYKETDPWV KTPLLIEMNP
 51 IHKKIPVLIH NGKPICESLI QLEYIDEVWS DASPILPSDP YQKSRARFWA
101 EFIDKKFYDP SWKVWATMGE EHAAVKKELL EHFKTLETEL GDKPYYGGEV
151 FGYLDIALMG YYSWFKAMEK FGEFSIETEF PKLTTWTKRC LERESVVKAL
201 ADSDRIIEYV YVLRKQFGAA
```

Spot No.:OR17

NCBI accession No.: **CAA52238.1**

Plant species: ***Arabidopsis thaliana***

Protein name: **RCI1B**

Peptide sequences: K.AAQDIAAADMAPTHPIR.L

PFF Mascot score: **[58]** Sequence coverage %: **[6]**

Matched peptides No.: **[1]**

Calculated Mr: **28239** Calculated *p*I: **4.97**

Annotated PFF spectra:

Ions score is -10*Log(P), where P is the probability that the observed match is a random event.
Individual ions scores > 35 indicate identity or extensive homology (p<0.05).
Protein scores are derived from ions scores as a non-probabilistic basis for ranking protein hits.

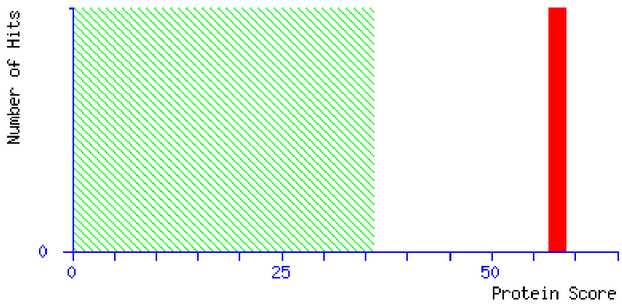

Matched peptides shown in bold red.

```
  1 MAATLGRDQY VYMAKLAEQA ERYEEMVQFM EQLVTGATPA EELTVEERNL
 51 LSVAYKNVIG SLRAAWRIVS SIEQKEESRK NDEHVSLVKD YRSKVESELS
101 SVCSGILKLL DSHLIPSAGA SESKVFYLKM KGDYHRYMAE FKSGDERKTA
151 AEDTMLAYKA AQDIAAADMA PTHPIRLGLA LNFSVFYYEI LNSSDKACNM
201 AKQAFEEAIA ELDTLGEESY KDSTLIMQLL RDNLTLWTSD YAGADGRGLR
251 I
```

Spot No.:OR18

NCBI accession No.: **NP_188267.1**

Plant species: ***Arabidopsis thaliana***

Protein name: **Mannose-binding lectin superfamily protein**

Peptide sequences: K.SGFQISAPEATGK.Q; K.IYASYGGEGIQYVK.F; K.VYVGQGQDGVAAVK.F; K.TAGPFGIVSGTK.F; K.LEGAGSEAGTLWDDGAFDGVR.K; K.FGVHVAPITK.-

PFF Mascot score: **[560]** Sequence coverage %: **[11]**

Matched peptides No.: **[6]**

Calculated Mr: **72430** Calculated *p*I: **5.31**

Annotated PFF spectra:

Ions score is -10*Log(P), where P is the probability that the observed match is a random event.
Individual ions scores > 35 indicate identity or extensive homology (p<0.05).
Protein scores are derived from ions scores as a non-probabilistic basis for ranking protein hits.

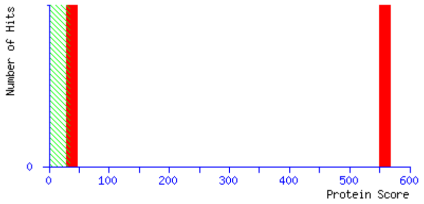

Matched peptides shown in bold red.

```
  1 MSWDDGSHAK VKKVQLTFDE IIYSIQVTYD GATALQSQLR GSVGPKSAEF
 51 TLAPDEYITA LSAYGKSLST QEVITALTFT TNKTSYGPYG TKSGFQISAP
101 EATGKQISGF LGTSGNVLNT IDVHYSPIPT GGTGTGGTGT GTGTGTGTGT
151 GTGTGTGTGT GTGTGTGGTG TGTGTGTGGT GTGTGTGGTG TGTGTGTGTG
201 TGTGTGTGTG TGGTGTGTGT GGTGTGTGTG TGTGTGTGGT GTGTGTGTGS
251 GAQKLEAQGN STGGTSWDDG SDYDGVTKIY ASYGGEGIQY VKFDYVKGGV
301 TKQGVLHGKQ QSRQNPREFV INHPDEYLVS VEGWYETVML GIQFKTNLNT
351 YEVSIYPFEP STDTKFTLQV QDKKIIGFHG FAGNHVNSIG AYFVPKSSTT
401 PVPSTPLKLT AEGGETGAVW DDGSHDDVKK VYVGQGQDGV AAVKFEYKNG
451 SQVVFGDERG TRTLLGFEEF ELESDEYITS VEGYYEKNFG VDTVVTTLIF
501 KTSKNKTAGP FGIVSGTKFE FKKEGYKITG FHGRAGEYVN AIGAYLAPSG
551 TTPLTPATQS QKLEGAGSEA GTLWDDGAFD GVRKVSVGQA QDGIGAVSFV
601 YDKAGQVVEG KEHGKPTLLG FEEFELDYPS EYITAVDGTY DAIFGNEPIV
651 NMLRFTTNKR VSIPFGIGAG TAFEFKKDGQ KIVGFHGRAG DLLHKFGVHV
701 APITK
```

Spot No.:OR19

NCBI accession No.: **AAF19535.1**

Plant species: ***Arabidopsis thaliana***

Protein name: **F23N19.3**

Peptide sequences: R.TAFHFQPEK.N; K.DFRDPTTAWK.T; R.TGISLIYDTTDFK.T; K.GWSSVQGIPR.T; K.NLVQWPVEEIK.S

PFF Mascot score: **[334]** Sequence coverage %: **[7]**

Matched peptides No.: **[5]**

Calculated Mr: **81758** Calculated *p*I: **7.26**

Annotated PFF spectra:

Ions score is -10*Log(P), where P is the probability that the observed match is a random event.
Individual ions scores > 35 indicate identity or extensive homology (p<0.05).
Protein scores are derived from ions scores as a non-probabilistic basis for ranking protein hits.

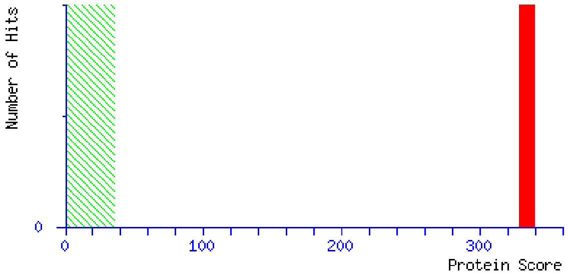

Matched peptides shown in bold red.

```
  1 MASTEALLPV TSLQDPLSES RSDQIPETRR RRPIKVHLAV YSGLLLIALY
 51 VTLIVTHDGS KAEIATESRP RMAGVSEKSN DGVWISSDDG KVEAFPWNNT
101 ILSWQRTAFH FQPEKNWMNG TNIKCPLFYK GWYHFFYQYN PNAAVWGDIV
151 WGHAVSKDLI HWLYLPIAMV PDQWYDANGV WTGSATFLDD GSIVMLYTGS
201 TDEFVQVQNL AYPEDPSDPL LLKWVKFSGN PVLVPPPGIG AKDFRDPTTA
251 WKTSSGKWRI TIGSKINRTG ISLIYDTTDF KTYEKHETLL HQVPNTGMWE
301 CVDFYPVSKT QLNGLDTSVN GPDVKHVIKA SMDDTRIDHY AIGTYDDSNA
351 TWVPDNPSID VGISTGLRYD YGKYYASKTF YDQNKGRRIL WGWIGESDSE
401 AADVQKGWSS VQGIPRTVVL DTRTHKNLVQ WPVEEIKSLR LSSKKFDMTI
451 GPGTVVPVDV GSATQLDIEA EFEIKTDDLK LFFDDDSVEA DNKFSCETNG
501 GSTARGALGP FGFSVLADEG LSEQTPVYFY VTKGKHSKLN TVFCTDTSRS
551 TLANDVVKPI YGSFVPVLKG EKLTMRILVD HSIVEGFAQG GRSCITSRVY
601 PTKAIYGATK LFLFNNAIDA TVTASFTVWQ MNNAFIHPYS SDDLGQILSL
651 TYLRTQSRAS SNAAFYFISY KTTKTVVTMT QYELKSLAVH KTITKQKSLS
701 FVKKKEGEKQ KQSHPRARKI SIRNLKSA
```

Spot No.:OR20

NCBI accession No.: **OAP11759.1**

Plant species: ***Arabidopsis thaliana***

Protein name: **GSTF8**

Peptide sequences: K.VHGVPMSTATMR.V; K.VHGVPMSTATMR.V + Oxidation (M); K.DLQFELIPVDMR.A; K.DLQFELIPVDMR.A + Oxidation (M); R.AITQYLAEEYSEKGEK.L; K.ATTNVWLQVEGQQFDPNASK.L

PFF Mascot score: **[341]** Sequence coverage %: **[22]**

Matched peptides No.: **[6]**

Calculated Mr: **29357** Calculated *p*I: **8.50**

Annotated PFF spectra:

Ions score is -10*Log(P), where P is the probability that the observed match is a random event. Individual ions scores > 35 indicate identity or extensive homology (p<0.05). Protein scores are derived from ions scores as a non-probabilistic basis for ranking protein hits.

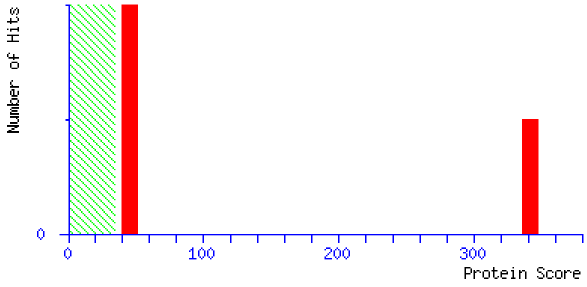

Matched peptides shown in bold red.

```
  1 MGAIQARLPL FLSPPSIKHH TFLHSSSSNS NFKIRSNKSS SSSSSSSIIM
 51 ASIKVHGVPM STATMRVLAT LYEKDLQFEL IPVDMRAGAH KQEAHLALNP
101 FGQIPALEDG DLTLFESRAI TQYLAEEYSE KGEKLISQDC KKVKATTNVW
151 LQVEGQQFDP NASKLAFERV FKGMFGMTTD PAAVQELEGK LQKVLDVYEA
201 RLAKSEFLAG DSFTLADLHH LPAIHYLLGT DSKVLFDSRP KVSEWIKKIS
251 ARPAWAKVID LQKQ
```

Spot No.:OR21

NCBI accession No.: **AAM61077.1**

Plant species: ***Arabidopsis thaliana***

Protein name: **Ferretin 1 precursor**

Peptide sequences: K.KADLAIPITSHASLAR.Q; K.ADLAIPITSHASLAR.Q; K.FFKESSEEER.G; K.KISDYITQLR.M; K.ISDYITQLR.M

PFF Mascot score: **[384]** Sequence coverage %: **[14]**

Matched peptides No.: **[5]**

Calculated Mr: **28156** Calculated *p*I: **5.73**

Annotated PFF **spectra:**

Ions score is -10*Log(P), where P is the probability that the observed match is a random event.
Individual ions scores > 35 indicate identity or extensive homology (p<0.05).
Protein scores are derived from ions scores as a non-probabilistic basis for ranking protein hits.

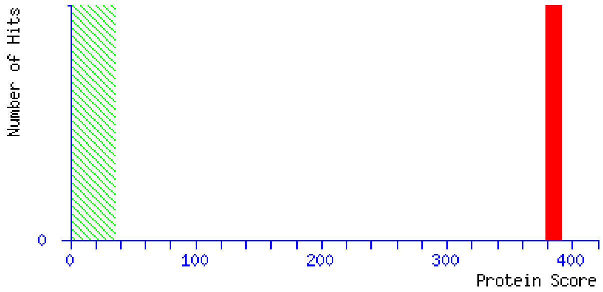

Matched peptides shown in bold red.

```
  1 MASNALSSFT AANPALSPKP LLPHGSASPS VSLGFSRKVG GSRAVVVAAA
 51 TVDTNNMPMT GVVFQPLEEV KKADLAIPIT SHASLARQRF ADASEAVINE
101 QINVEYNVSY VYHSMYAYFD RDNVAMKGLA KFFKESSEEE RGHAEKFMEY
151 QNQRGGRVKL HPIVSPISEF EHAEKGDALY AMELALSLEK LTNEKLLNVH
201 KVASENNDPQ LADFVESEFL GEQIEAIKKI SDYITQLRMI GKGHGVWHFD
251 QMLLN
```

Spot No.:OR22

NCBI accession No.: **AAM63854.1**

Plant species: ***Arabidopsis thaliana***

Protein name: **Atpm24.1 *glutathione S-transferase***

Peptide sequences:K.VFGHPASIATR.R;

R.NPFGQVPAFEDGDLK.L; R.AITQYIAHR.Y;

R.YENQGTNLLQTDSK.N; K.SIYGLTTDEAVVAEEEAK.L;

R.VNEWVAEITK.R

PFF Mascot score: **[526]** Sequence coverage %: **[36]**

Matched peptides No.: **[6]**

Calculated Mr: **24036** Calculated *p*I: **6.08**

Annotated PFF spectra:

Ions score is -10*Log(P), where P is the probability that the observed match is a random event.
Individual ions scores > 35 indicate identity or extensive homology (p<0.05).
Protein scores are derived from ions scores as a non-probabilistic basis for ranking protein hits.

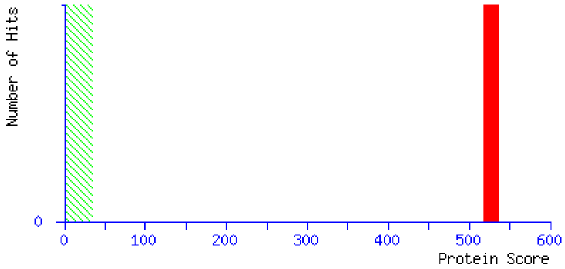

Matched peptides shown in bold red.

```
  1 MAGIKVFGHP ASIATRRVLI ALHEKNLDFE LVHVELKDGE HKKEPFLSRN
 51 PFGQVPAFED GDLKLFESRA ITQYIAHRYE NQGTNLLQTD SKNISQYAIM
101 AIGMQVEDHQ FDPVASKLAF EQISKSIYGL TTDEAVVAXE EAKLAKVLDV
151 YEARLKEFKY LAGETFTLTD LHHIPAIQYL LGTPTKKLFT ERPRVNEWVA
201 EITKRPASEK VQ
```

Spot No.:OR23

NCBI accession No.: **NP_195637.1**

Plant species: ***Arabidopsis thaliana***

Protein name: **Cold, circadian rhythm, and RNA binding 1 （Glycine-rich RNA-binding protein 8）**

Peptide sequences: R.CFVGGLAWATNDEDLQR.T; R.GFGFVTFKDEK.A; R.VITVNEAQSR.G; R.SGGGGGYSGGGGGGYSGGGGGGYER.R

PFF Mascot score: **[528]** Sequence coverage %: **[37]**

Matched peptides No.: **[4]**

Calculated Mr: **16626** Calculated *p*I: **5.58**

Annotated PFF **spectra:**

Ions score is -10*Log(P), where P is the probability that the observed match is a random event.
Individual ions scores > 35 indicate identity or extensive homology (p<0.05).
Protein scores are derived from ions scores as a non-probabilistic basis for ranking protein hits.

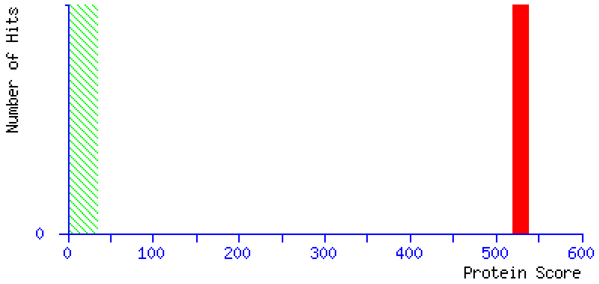

Matched peptides shown in bold red**.**

```
  1 MSEVEYRCFV GGLAWATNDE DLQRTFSQFG DVIDSKIIND RESGRSRGFG
 51 FVTFKDEKAM RDAIEEMNGK ELDGRVITVN EAQSRGSGGG GGGRGGSGGG
101 YRSGGGGGYS GGGGGGYSGG GGGGYERRSG GYGSGGGGGG RGYGGGGRRE
151 GGGYGGGDGG SYGGGGGGW
```

Spot No.:ML1

NCBI accession No.: **AAL06896.1**

Plant species: ***Arabidopsis thaliana***

Protein name:**AT5g26000/T1N24_7**

Peptide sequences: K.LFNSGNFEK.G; R.GYALGTDAPGR.C; R.EYVGDRLPEFSETEAALVK.G

PFF Mascot score: **[238]** Sequence coverage %: **[7]**

Matched peptides No.: **[3]**

Calculated Mr: **61704** Calculated *p*I: **5.67**

Annotated PFF spectra:

Ions score is -10*Log(P), where P is the probability that the observed match is a random event.
Individual ions scores > 35 indicate identity or extensive homology (p<0.05).
Protein scores are derived from ions scores as a non-probabilistic basis for ranking protein hits.

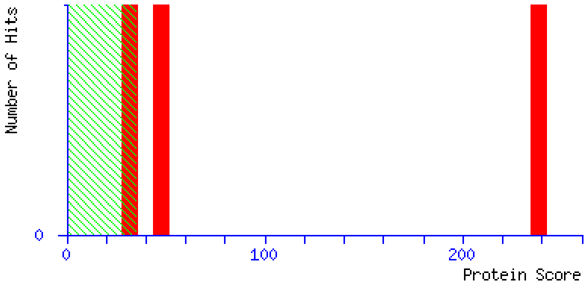

Matched peptides shown in bold red.

```
  1 MKLLMLAFVF LLALATCKGD EFVCEENEPF TCNQTKLFNS GNFEKGFIFG
 51 VASSAYQVEG GRGRGLNVWD SFTHRFPEKG GADLGNGDTT CDSYTLWQKD
101 IDVMDELNST GYRFSIAWSR LLPKGKRSRG VNPGAIKYYN GLIDGLVAKN
151 MTPFVTLFHW DLPQTLQDEY NGFLNKTIVD DFKDYADLCF ELFGDRVKNW
201 ITINQLYTVP TRGYALGTDA PGRCSPKIDV RCPGGNSSTE PYIVAHNQLL
251 AHAAAVDVYR TKYKDDQKGM IGPVMITRWF LPFDHSQESK DATERAKIFF
301 HGWFMGPLTE GKYPDIMREY VGDRLPEFSE TEAALVKGSY DFLGLNYYVT
351 QYAQNNQTIV PSDVHTALMD SRTTLTSKNA TGHAHGPPFN AASYYYPKGI
401 YYVMDYFKTT YGDPLIYVTE NGFSTPGDED FEKATADYKR IDYLCSHLCF
451 LSKVIKEKNV NVKGYFAWSL GDNYEFCNGF TVRFGLSYVD FANITGDRDL
501 KASGKWFQKF INVTDEDSTN QDLLRSSVSS KNRDRKSLAD A
```

Spot No.: **ML3**

NCBI accession No.: **CAA61592.1**

Plant species: ***Arabidopsis thaliana***

Protein name： **Thioglucoside glucohydrolase**

Peptide sequences: R.GPALWDIYCR.R; K.DFLSQGVRPSALK.K; R.CNNDNGDVAVDFFHR.Y

PFF Mascot score: **[183]** Sequence coverage %: **[7]**

Matched peptides No.: **[3]**

Calculated Mr: **60222** Calculated *p*I: **6.45**

Annotated PFF spectra:

Ions score is -10*Log(P), where P is the probability that the observed match is a random event.
Individual ions scores > 35 indicate identity or extensive homology (p<0.05).
Protein scores are derived from ions scores as a non-probabilistic basis for ranking protein hits.

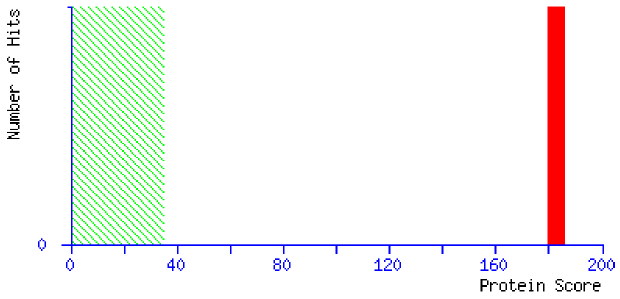

Matched peptide sequences: shown in Bold Red

```
  1 MVLQKLPLIG LLLLLTIVAS PANADGPVCP PSNKLSRASF PEGFLFGTAT
 51 AAYQVEGAIN ETCRGPALWD IYCRRYPERC NNDNGDVAVD FFHRYKEDIQ
101 LMKNLNTDAF RMSIAWPRIF PHGRKEKGVS QAGVQFYHDL IDELIKNGIT
151 PFVTVFHWDT PQDLEDEYGG FLSERIVKDF REYADFVFQE YGGKVKHWIT
201 FNEPWVFSHA GYDVGKKAPG RCSSYVNAKC QDGRSGYEAY LVTHNLLISH
251 AEAVEAYRKC EKCKGGKIGI AHSPAWFEAH DLADSQDGAS IDRALDFILG
301 WHLDTTTFGD YPQIMKDIVG HRLPKFTTEQ KAKLKASTDF VGLNYYTSVF
351 SNHLEKPDPS KPRWMQDSLI TWESKNAQNY AIGSKPLTAA LNVYSRGFRS
401 LLKYIKDKYA NPEIMIMENG YGEELGASDS VAVGTADHNR KYYLQRHLLS
451 MQEAVCIDEV NVTGYFVWSL LDNFEWQDGY KNRFGLYYVD FKNNLTRYEK
501 ESGKYYKDFL SQGVRPSALK KDEL
```

Spot No.: **ML4**

NCBI accession No.: **NP_051044.1**

Plant species: ***Arabidopsis thaliana***

Protein name： **ATP synthase CF1 alpha subunit**

Peptide sequences: R.ADEISNIIR.E; K.IAQIPVSEAYLGR.V; R.VINALANPIDGR.G; R.EQHTLIIYDDLSK.Q; R.EAYPGDVFYLHSR.L; K.EGIQEQLER.F

PFF Mascot score: **[506]** Sequence coverage %: **[13]**

Matched peptides No.: **[6]**

Calculated Mr: **55351** Calculated *p*I: **5.19**

Annotated PFF spectra:

Ions score is -10*Log(P), where P is the probability that the observed match is a random event. Individual ions scores > 35 indicate identity or extensive homology (p<0.05).
Protein scores are derived from ions scores as a non-probabilistic basis for ranking protein hits.

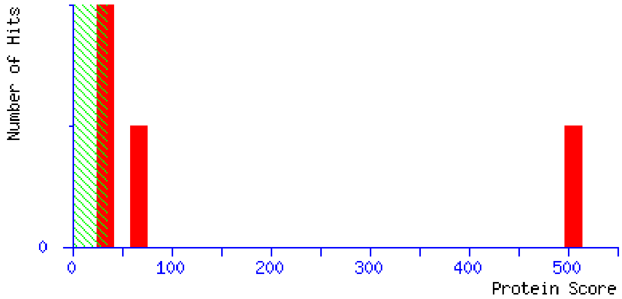

Matched peptide sequences: shown in Bold Red

```
  1 MVTIRADEIS NIIRERIEQY NREVTIVNTG TVLQVGDGIA RIYGLDEVMA
 51 GELVEFEEGT IGIALNLESN NVGVVLMGDG LMIQEGSSVK ATGKIAQIPV
101 SEAYLGRVIN ALANPIDGRG KISASESRLI ESPAPGIISR RSVYEPLQTG
151 LIAIDSMIPI GRGQRELIIG DRQTGKTAVA TDTILNQQGQ NVICVYVAIG
201 QKASSVAQVV TSLQERGAME YTIVVAETAD SPATLQYLAP YTGAALAEYF
251 MYREQHTLII YDDLSKQAQA YRQMSLLLRR PPGREAYPGD VFYLHSRLLE
301 RAAKLSSQLG EGSMTALPIV ETQSGDVSAY IPTNVISITD GQIFLSADLF
351 NAGIRPAINV GISVSRVGSA AQIKAMKQVA GKLKLELAQF AELEAFSQFS
401 SDLDKATQNQ LARGQRLREL LKQSQSAPLT VEEQIMTIYT GTNGYLDGLE
451 IGQVRKFLVQ LRTYLKTNKP QFQEIIASTK TLTAEAESFL KEGIQEQLER
501 FLLQEKV
```

Spot No.: **ML5**

NCBI accession No.: **OAP04789.1**

Plant species: ***Arabidopsis thaliana***

Protein name: **HCEF1（fructose 1,6-bisphosphatase）**

Peptide sequences: U R.YIGSLVGDFHR.T; R.TLLYGGIYGYPR.D; R.VLDIQPTEIHQR.V

PFF Mascot score: **[214]** Sequence coverage %: **[8]**

Matched peptides No.: **[3]**

Calculated Mr: **45592** Calculated *p*I: **5.25**

Annotated PFF **spectra:**

Ions score is -10*Log(P), where P is the probability that the observed match is a random event. Individual ions scores > 35 indicate identity or extensive homology (p<0.05). Protein scores are derived from ions scores as a non-probabilistic basis for ranking protein hits.

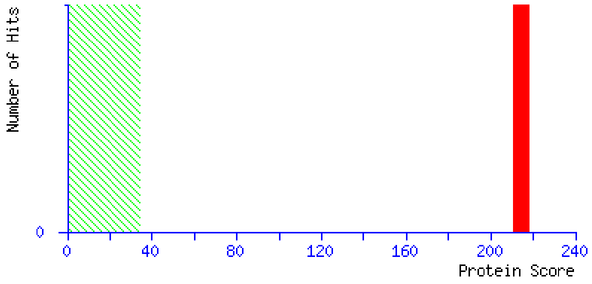

Matched peptide sequences: shown in **Bold Red**

```
  1 MAASAATTTS SHLLLSSSRH VASSSQPSIL SPRSLFSNNG KRAPTGVRNH
 51 QYASGVRCMA VAADASETKT AARKKSGYEL QTLTGWLLRQ EMKGEIDAEL
101 TIVMSSISLA CKQIASLVQR AGISNLTGVQ GAVNIQGEDQ KKLDVISNEV
151 FSNCLRSSGR TGIIASEEED VPVAVEESYS GNYVVVFDPL DGSSNIDAAV
201 STGSIFGIYS PNDECIVDDS DDISALGSEE QRCIVNVCQP GNNLLAAGYC
251 MYSSSVIFVL TLGKGVFSFT LDPMYGEFVL TQENIEIPKA GRIYSFNEGN
301 YQMWDDKLKK YIDDLKDPGP TGKPYSARYI GSLVGDFHRT LLYGGIYGYP
351 RDAKSKNGKL RLLYECAPMS FIVEQAGGKG SDGHSRVLDI QPTEIHQRVP
401 LYIGSTEEVE KLEKYLA
```

Spot No.: **ML6**

NCBI accession No.: **AAN18180.1**

Plant species: ***Arabidopsis thaliana***

Protein name： **At2g39730/T5I7.3**

Peptide sequences: R.GLAYDTSDDQQDITR.G; U.R.YREAADLIK.K; R.VPIICTGNDFSTLYAPLIR.D; R.EGPPVFEQPEMTYEK.L;R.VQLAETYLSQAALGDANADAIGR.G; K.GAQQVNLPVPEGCTDPVAENFDPTAR

PFF Mascot score: **[517]** Sequence coverage %: **[22]**

Matched peptides No.: **[6]**

Calculated Mr: **52371** Calculated *p*I: **5.69**

Annotated PFF **spectra:**

Ions score is -10*Log(P), where P is the probability that the observed match is a random event.
Individual ions scores > 35 indicate identity or extensive homology (p<0.05).
Protein scores are derived from ions scores as a non-probabilistic basis for ranking protein hits.

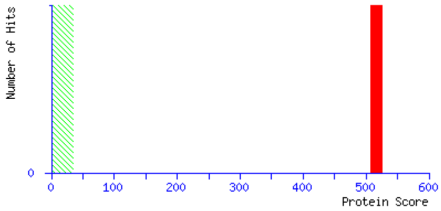

Matched peptide sequences: shown in **Bold Red**

```
  1 MAAAVSTVGA INRAPLSLNG SGSGAVSAPA STFLGKKVVT VSRFAQSNKK
 51 SNGSFKVLAV KEDKQTDGDR WRGLAYDTSD DQQDITRGKG MVDSVFQAPM
101 GTGTHHAVLS SYEYVSQGLR QYNLDNMMDG FYIAPAFMDK LVVHITKNFL
151 TLPNIKVPLI LGIWGGKGQG KSFQCELVMA KMGINPIMMS AGELESGNAG
201 EPAKLIRQRY REAADLIKKG KMCCLLINDL DAGAGRMGGT TQYTVNNQMV
251 NATLMNIADN PTNVQLPGMY NKEENARVPI ICTGNDFSTL YAPLIRDGRM
301 EKFYWAPTRE DRIGVCKGIF RTDKIKDEDI VTLVDQFPDQ SIDFFGALRA
351 RVYDDEVRKF VESLGVEKIG KRLVNSREGP PVFEQPEMTY EKLMEYGNML
401 VMEQENVKRV QLAETYLSQA ALGDANADAI GRGTFYGKGA QQVNLPVPEG
451 CTDPVAENFD PTARSDDGTC VYNF
```

Spot No.: **ML7**

NCBI accession No.: **AAO19414.1**

Plant species: ***Arabidopsis thaliana***

Protein name： **Ribulose-1,5-bisphosphate carboxylase**

Peptide sequences: K.DTDILAAFR.V; K.TFQGPPHGIQVER.D; R.DNGLLLHIHR.A; R.ESTLGFVDLLR.D; R.VALEACVQAR.N; R.DLAVEGNEIIR.-

PFF Mascot score: **[488]** Sequence coverage %: **[14]**

Matched peptides No.: **[6]**

Calculated Mr: **47920** Calculated *p*I: **6.12**

Annotated PFF spectra:

Ions score is -10*Log(P), where P is the probability that the observed match is a random event.
Individual ions scores > 35 indicate identity or extensive homology (p<0.05).
Protein scores are derived from ions scores as a non-probabilistic basis for ranking protein hits.

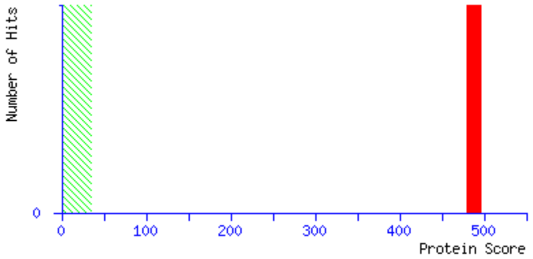

Matched peptide sequences: shown in Bold Red

```
  1 KLTYYTPEYE TKDTDILAAF RVTPQPGVPP EEAGAAVAAE SSTGTWTTVW
 51 TDGLTSLDRY KGRCYHIEPV PGEETQFIAY VAYPLDLFEE GSVTNMFTSI
101 VGNVFGFKAL AALRLEDLRI PPAYTKTFQG PPHGIQVERD KLNKYGRPLL
151 GCTIKPKLGL SAKNYGRAVY ECLRGGLDFT KDDENVNSQP FMRWRDRFLF
201 CAEAIYKSQA ETGEIKGHYL NATAGTCEEM IKRAVFAREL GVPIVMHDYL
251 TGGFTANTSL SHYCRDNGLL LHIHRAMHAV IDRQKNHGMH FRVLAKALRL
301 SGGDHIHAGT VVGKLEGDRE STLGFVDLLR DDYVEKDRSR GIFFTQDWVS
351 LPGVLPVASG GIHVWHMPAL TEIFGDDSVL QFGGGTLGHP WGNAPGAVAN
401 RVALEACVQA RNEGRDLAVE GNEIIREACK
```

Spot No.: **ML9**

NCBI accession No.: **AAN18180.1**

Plant species: ***Arabidopsis thaliana***

Protein name: **At2g39730/T5I7.3**

Peptide sequences: R.GLAYDTSDDQQDITR.G; K.SFQCELVMAK.M; R.VQLAETYLSQAALGDANADAIGR.G

PFF Mascot score: **[214]** Sequence coverage %: **[10]**

Matched peptides No.: **[3]**

Calculated Mr: **52371** Calculated *p*I: **5.69**

Annotated PFF **spectra:**

Ions score is -10*Log(P), where P is the probability that the observed match is a random event.
Individual ions scores > 35 indicate identity or extensive homology (p<0.05).
Protein scores are derived from ions scores as a non-probabilistic basis for ranking protein hits.

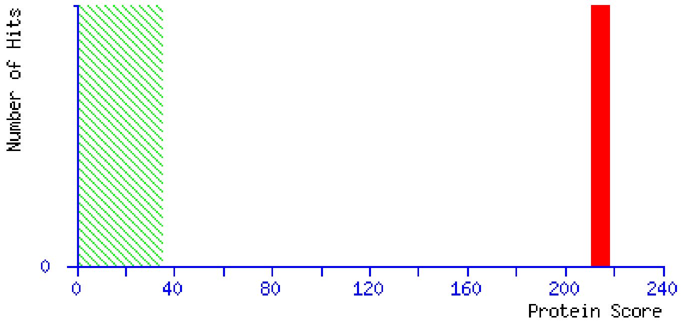

Matched peptide sequences: shown in Bold Red

```
  1 MAAAVSTVGA INRAPLSLNG SGSGAVSAPA STFLGKKVVT VSRFAQSNKK
 51 SNGSFKVLAV KEDKQTDGDR WRGLAYDTSD DQQDITRGKG MVDSVFQAPM
101 GTGTHHAVLS SYEYVSQGLR QYNLDNMMDG FYIAPAFMDK LVVHITKNFL
151 TLPNIKVPLI LGIWGGKGQG KSFQCELVMA KMGINPIMMS AGELESGNAG
201 EPAKLIRQRY REAADLIKKG KMCCLLINDL DAGAGRMGGT TQYTVNNQMV
251 NATLMNIADN PTNVQLPGMY NKEENARVPI ICTGNDFSTL YAPLIRDGRM
301 EKFYWAPTRE DRIGVCKGIF RTDKIKDEDI VTLVDQFPDQ SIDFFGALRA
351 RVYDDEVRKF VESLGVEKIG KRLVNSREGP PVFEQPEMTY EKLMEYGNML
401 VMEQENVKRV QLAETYLSQA ALGDANADAI GRGTFYGKGA QQVNLPVPEG
451 CTDPVAENFD PTARSDDGTC VYNF
```

Spot No.: **ML14**

NCBI accession No.: **NP_566728.1**

Plant species: ***Arabidopsis thaliana***

Protein name: **Plastid-lipid associated protein PAP / fibrillin family protein**

Peptide sequences: R.GLVASVDDLER.A; R.LLYSSAFSSR.S; K.TSGNLSQIPPFDIPR.L

PFF Mascot score: **[216]** Sequence coverage %: **[12]**

Matched peptides No.: **[3]**

Calculated Mr: **30493** Calculated *p*I: **5.82**

Annotated PFF **spectra:**

Ions score is -10*Log(P), where P is the probability that the observed match is a random event.
Individual ions scores > 35 indicate identity or extensive homology (p<0.05).
Protein scores are derived from ions scores as a non-probabilistic basis for ranking protein hits.

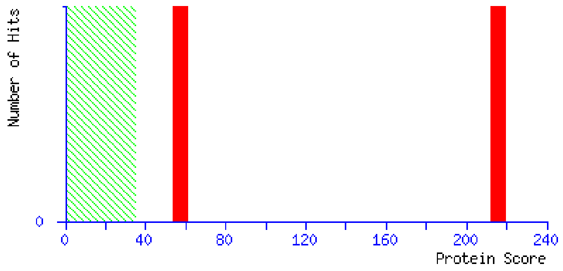

Matched peptide sequences: shown in Bold Red

```
  1 MATSSTFSSL LPSPPALLSD HRSPPPSIRY SFSPLTTPKS SRLGFTVPEK
 51 RNLAANSSLV EVSIGGESDP PPSSSGSGGD DKQIALLKLK LLSVVSGLNR
101 GLVASVDDLE RAEVAAKELE TAGGPVDLTD DLDKLQGKWR LLYSSAFSSR
151 SLGGSRPGLP TGRLIPVTLG QVFQRINVFS KDFDNIAEVE LGAPWPFPPL
201 EATATLAHKF ELLGTCKIKI TFEKTTVKTS GNLSQIPPFD IPRLPDSFRP
251 SSNPGTGDFE VTYVDDTMRI TRGDRGELRV FVIA
```

Spot No.: **ML15**

NCBI accession No.: **AAL07227.1**

Plant species: ***Arabidopsis thaliana***

Protein name: **Putative lactoylglutathione lyase**

Peptide sequences: K.DPDGYTFELIQR.G; R.GPTPEPFCQVMLR.V; R.EAGPLPGLGTK.I

PFF Mascot score: **[217]** Sequence coverage %: **[12]**

Matched peptides No.: **[3]**

Calculated Mr: **31995** Calculated *p*I: **5.11**

Annotated PFF **spectra:**

Ions score is -10*Log(P), where P is the probability that the observed match is a random event.
Individual ions scores > 35 indicate identity or extensive homology (p<0.05).
Protein scores are derived from ions scores as a non-probabilistic basis for ranking protein hits.

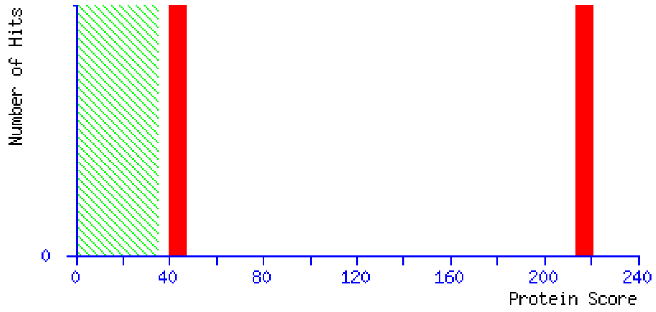

Matched peptide sequences: shown in **Bold Red**

```
  1 MAEASDLLEW PKKDNRRFLH VVYRVGDLDR TIEFYTEVFG MKLLWKRDIP
 51 EEKYSNAFLG FGPETSNFVV ELTYNYGVSS YDIGTGFGHF AISTQDVSKL
101 VENVRAKGGN VTREPGPVKG GGSVIAFVKD PDGYTFELIQ RGPTPEPFCQ
151 VMLRVGDLDR AIKFYEKALG MRLLRKIERP EYKYTIGMMG YAEEYESIVL
201 ELTYNYDVTE YTKGNAYAQI AIGTDDVYKS GEVIKIVNQE LGGKITREAG
251 PLPGLGTKIV SFLDPDGWKT VLVDNKDFLK ELE
```

Spot No.: **ML17**

NCBI accession No.: **CAA63909.1**

Plant species: ***Arabidopsis thaliana***

Protein name： **2-Cys peroxiredoxin bas1**

Peptide sequences: K.SFGVLIHDQGIALR.G

PFF Mascot score: **[97]** Sequence coverage %: **[5]**

Matched peptides No.: **[1]**

Calculated Mr: **28966** Calculated *p*I: **8.76**

Annotated PFF **spectra:**

Ions score is -10*Log(P), where P is the probability that the observed match is a random event.
Individual ions scores > 35 indicate identity or extensive homology (p<0.05).
Protein scores are derived from ions scores as a non-probabilistic basis for ranking protein hits.

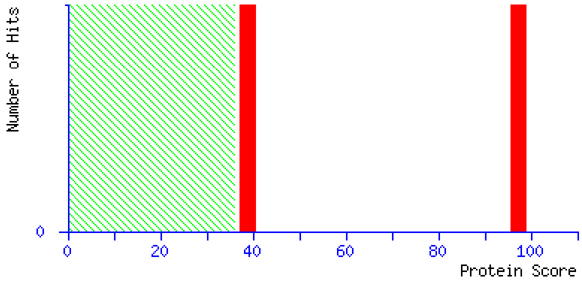

Matched peptide sequences: shown in Bold Red

```
  1 MASVASSTTL ISSPSSRVFP AKSSLSSPSV SFLRTLSSPS ASASLRSGFA
 51 RRSSLSSTSR RSFAVKAQAD DLPLVGNKAP DFKAEAVFDQ EFIKVKLSDY
101 NGKKYVILFF YPLDFTFVCP TEITAFSDRH SEFEKLNTEV LGVSVDSVFS
151 HLAWVQTDRK SGGLGDLNYP LISDVTKSIS KSFGVLIHDQ GIALRGLFII
201 DKEGVIQHST INNLGIGRSV DETMRTLQAL QYTGNPDEVC PAGWKSGEKS
251 MKPDPKLSKE YFSAI
```

Spot No.: **ML18**

NCBI accession No.: AAM63854.1

Plant species: ***Arabidopsis thaliana***

Protein name：**Atpm24.1 glutathione S transferase**

Peptide sequences: K.VFGHPASIATR.R; R.NPFGQVPAFEDGDLK.L; R.YENQGTNLLQTDSK.N

PFF Mascot score: **[218]** Sequence coverage %: **[18]**

Matched peptides No.: **[3]**

Calculated Mr: **24036** Calculated *p*I: **6.08**

Annotated PFF **spectra:**

Ions score is -10*Log(P), where P is the probability that the observed match is a random event.
Individual ions scores > 35 indicate identity or extensive homology (p<0.05).
Protein scores are derived from ions scores as a non-probabilistic basis for ranking protein hits.

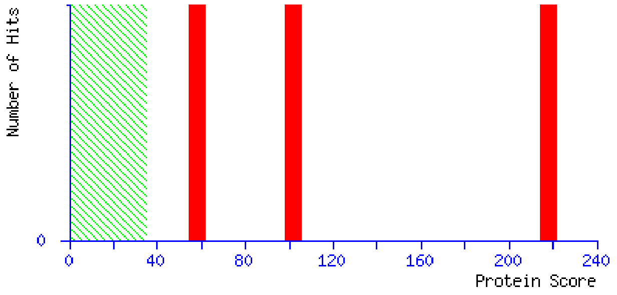

Matched peptide sequences: shown in Bold Red

```
  1 MAGIKVFGHP ASIATRRVLI ALHEKNLDFE LVHVELKDGE HKKEPFLSRN
 51 PFGQVPAFED GDLKLFESRA ITQYIAHRYE NQGTNLLQTD SKNISQYAIM
101 AIGMQVEDHQ FDPVASKLAF EQISKSIYGL TTDEAVVAXE EAKLAKVLDV
151 YEARLKEFKY LAGETFTLTD LHHIPAIQYL LGTPTKKLFT ERPRVNEWVA
201 EITKRPASEK VQ
```

Spot No.: **ML19**

NCBI accession No.: **AAM64537.1**

Plant species: ***Arabidopsis thaliana***

Protein name：**Putative 2-cys peroxiredoxin BAS1 precursor**

Peptide sequences: **K.APDFEAEAVFDQEFIK.V;**

K.SFGVLIHDQGIALR.G; K.SFGVLIHDQGIALR.G

PFF Mascot score: **[164]**　　Sequence coverage %: **[11]**

Matched peptides No.: **[3]**

Calculated Mr: **29256**　　Calculated *p*I: **6.91**

Annotated PFF spectra:

Ions score is -10*Log(P), where P is the probability that the observed match is a random event.
Individual ions scores > 35 indicate identity or extensive homology (p<0.05).
Protein scores are derived from ions scores as a non-probabilistic basis for ranking protein hits.

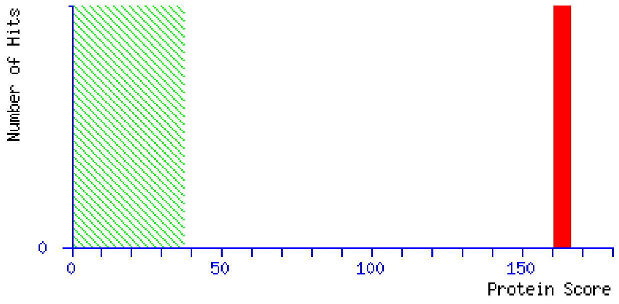

Matched peptide sequences: shown in Bold Red

```
  1 MASVASSTTL ISSPSSRVFP AKSSLSSPSV SFLRTLSSPS ASASLRSGFA
 51 RRSSLSSTSR RSFAVKAQAD DLPLVGNKAP DFEAEAVFDQ EFIKVKLSDY
101 IGKKYVILFF YPLDFTFVCP TEITAFSDRH SEFEKLNTEV LGVSVDSVFS
151 HLAWVQTDRK SGGLGDLNYP LISYFTKSIS KSFGVLIHDQ GIALRGLFII
201 DKEGVIQHST INNLGIGQSV DETMRTLQAL QYIQENPDEV CPAGWKPGEK
251 SMKPDPKLSK EYFSAI
```

Spot No.: **ML20**

NCBI accession No.: **AAM64537.1**

Plant species: ***Arabidopsis thaliana***

Protein name： **putative 2-cys peroxiredoxin BAS1 precursor**

Peptide sequences: K.APDFEAEAVFDQEFIK.V; K.SFGVLIHDQGIALR.G; K.SFGVLIHDQGIALR.G

PFF Mascot score: **[164]** Sequence coverage %: **[11]**

Matched peptides No.: **[3]**

Calculated Mr: **29256** Calculated *p*I: **6.91**

Annotated PFF **spectra:**

Ions score is -10*Log(P), where P is the probability that the observed match is a random event.
Individual ions scores > 35 indicate identity or extensive homology (p<0.05).
Protein scores are derived from ions scores as a non-probabilistic basis for ranking protein hits.

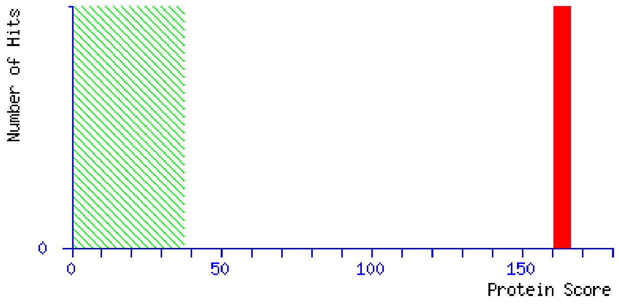

Matched peptide sequences: shown in **Bold Red**

```
  1 MASVASSTTL ISSPSSRVFP AKSSLSSPSV SFLRTLSSPS ASASLRSGFA
 51 RRSSLSSTSR RSFAVKAQAD DLPLVGNKAP DFEAEAVFDQ EFIKVKLSDY
101 IGKKYVILFF YPLDFTFVCP TEITAFSDRH SEFEKLNTEV LGVSVDSVFS
151 HLAWVQTDRK SGGLGDLNYP LISYFTKSIS KSFGVLIHDQ GIALRGLFII
201 DKEGVIQHST INNLGIGQSV DETMRTLQAL QYIQENPDEV CPAGWKPGEK
251 SMKPDPKLSK EYFSAI
```

Spot No.: **ML21**

NCBI accession No.: **OAP02726.1**

Plant species: ***Arabidopsis thaliana***

Protein name：**PBPI （PYK10-binding protein 1）**

Peptide sequences: K.GANLWDDGSTHDAVTK.I; K.VYVGQAQDGISAVK.F; K.FVYDKSPEEVTGEEHGK.S; K.IGVHVRPLSN.-

PFF Mascot score: **[208]** Sequence coverage %: **[19]**

Matched peptides No.: **[4]**

Calculated Mr: **32110** Calculated *p*I: **5.46**

Annotated PFF **spectra:**

Ions score is -10*Log(P), where P is the probability that the observed match is a random event.
Individual ions scores > 35 indicate identity or extensive homology (p<0.05).
Protein scores are derived from ions scores as a non-probabilistic basis for ranking protein hits.

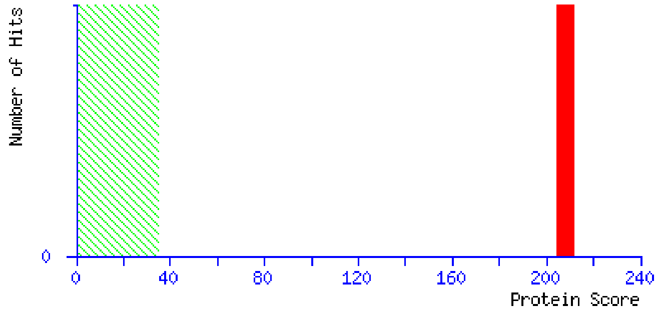

Matched peptide sequences: shown in Bold Red

```
  1 MAQKVEAQGG KGANLWDDGS THDAVTKIQL AAGIDGIQYV QFDYVKNGQP
 51 EQAPLRGTKG RVLPADPFVI NHPDEHLVSV EGWYSPEGII QGIKFISNKK
101 TSDVIGSDEG THFTLQVKDK KIIGFHGSAG GNLNSLGAYF APLTTTTPLT
151 PAKQLTAFGS DDGTAWDDGA YVGVKKVYVG QAQDGISAVK FVYDKSPEEV
201 TGEEHGKSTL LGFEEFVLDY PSEYITAVDG TYDKIFGSDG SVITMLRFKT
251 NKQTSPPFGL EAGTVFELKE EGHKIVGFHG RADVLLHKIG VHVRPLSN
```

Spot No.: ML22

NCBI accession No.: **CAA31948.1**

Plant species: ***Arabidopsis thaliana***

Protein name: **Ribulose bisphosphate carboxylase**

Peptide sequences: R.EHGNSPGYYDGR.Y; K.EYPNAFIR.I

PFF Mascot score: **[80]** Sequence coverage %: **[10]**

Matched peptides No.: **[2]**

Calculated Mr: **20733** Calculated *p*I: **7.59**

Annotated PFF **spectra:**

Ions score is -10*Log(P), where P is the probability that the observed match is a random event. Individual ions scores > 35 indicate identity or extensive homology (p<0.05).
Protein scores are derived from ions scores as a non-probabilistic basis for ranking protein hits.

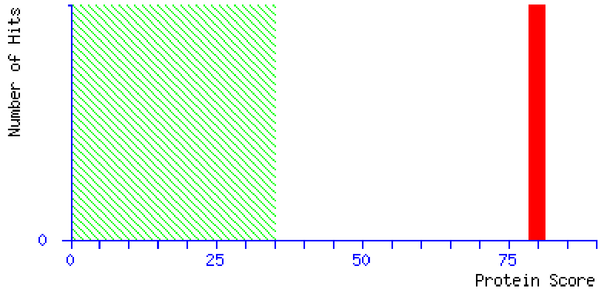

Matched peptide sequences: shown in Bold Red

```
  1 MASSMLSSAT MVASPAQATM VAPFNGLKSS AAFPATRKAN NDITSITSNG
 51 GRVNCMQMQV WPPIGKKKFE TLSYLPDLTD SELAKEVDYL IRNKWIPCVE
101 FELEHGFVYR EHGNSPGYYD GRYWTMWKLP LFGCTDSAQV LKEVEECKKE
151 YPNAFIRIIG FDNTRQVQCI SFVAYKPPSF TG
```

Spot No.: **ML23**

NCBI accession No.: **CAA36675.1**

Plant species: ***Arabidopsis thaliana***

Protein name: **33 kDa oxygen-evolving protein**

Peptide sequences: **K.RLTYDEIQSK.T; K.NAPPEFQNTK.L; K.QLDASGKPDSFTGK.F**

PFF Mascot score: **[140]** Sequence coverage %: **[10]**

Matched peptides No.: **[3]**

Calculated Mr: **35285** Calculated *p*I: **5.68**

Annotated PFF **spectra:**

Ions score is -10*Log(P), where P is the probability that the observed match is a random event.
Individual ions scores > 35 indicate identity or extensive homology (p<0.05).
Protein scores are derived from ions scores as a non-probabilistic basis for ranking protein hits.

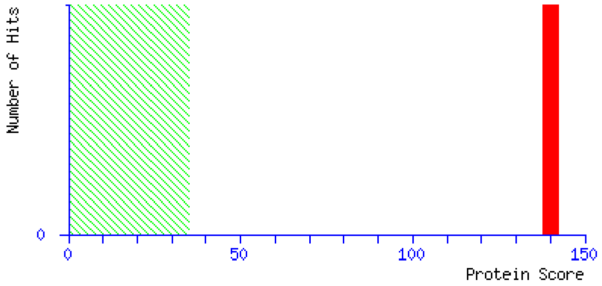

Matched peptide sequences: shown in Bold Red

```
  1 MAASLQSTAT FLQSAKIATA PSRGSSHLRS TQAVGKSFGL ETSSARLTCS
 51 FQSDFKDFTG KCSDAVKIAG FALASSALVV SGASAEGAPK RLTYDEIQSK
101 TYMEVKGTGT ANQCPTIDGG SETFSFKPGK YAGKKFCFEP TSFTVKADSV
151 SKNAPPEFQN TKLMTRLTYT LDEIEGPFEV ASDGSVNFKE EDGIDYAAVT
201 VQLPGGERVP FLFTVKQLDA SGKPDSFTGK FLVPSYRGSS FLDPKGRGGS
251 TGYDNAVALP AGGRGDEEEL VKENVKNTAA SVGEITLKVT KSKPETGEVI
301 GVFESLQPSD TDLGAKVPKD VKIQGVWYGQ LE
```

Spot No.: **ML24**

NCBI accession No.: **AAG30126.1**

Plant species: ***Arabidopsis thaliana***

Protein name: **Glutathione S-transferase**

Peptide sequences: K.VFGHPASTATR.R; K.VPAFEDGDFK.L; K.VLDVYEHR.L

PFF Mascot score: **[206]** Sequence coverage %: **[13]**

Matched peptides No.: **[3]**

Calculated Mr: **23539** Calculated *p*I: **6.31**

Annotated PFF **spectra:**

Ions score is -10*Log(P), where P is the probability that the observed match is a random event.
Individual ions scores > 35 indicate identity or extensive homology (p<0.05).
Protein scores are derived from ions scores as a non-probabilistic basis for ranking protein hits.

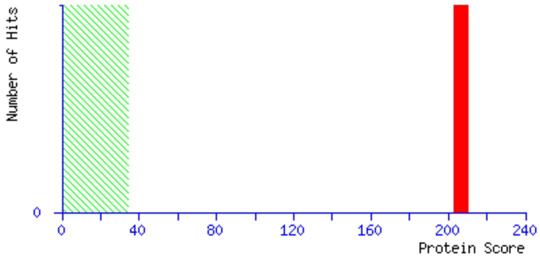

Matched peptide sequences: shown in Bold Red

```
  1 MAGIKVFGHP ASTATRRVLI ALHEKNLDFE FVHIELKDGE HKKEPFIFRN
 51 PFGKVPAFED GDFKLFESRA ITQYIAHFYS DKGNQLVSLG SKDIAGIAMG
101 IEIESHEFDP VGSKLVWEQV LKPLYGMTTD KTVVEEEEAK LAKVLDVYEH
151 RLGESKYLAS DKFTLVDLHT IPVIQYLLGT PTKKLFAERP HVSAWVADIT
201 SRPSAKKVL
```

Spot No.: **MR1**

NCBI accession No.: **OAP03586.1**

Plant species: ***Arabidopsis thaliana***

Protein name：**Hypothetical protein AXX17_AT3G17230（plant invertase/pectin methylesterase inhibitor）**

Peptide sequences: K.HLYVFDLETR.T; K.LLTPVEEGPTPR.S; R.LDKFGGEEETPSSR.G

PFF Mascot score: **[180]** Sequence coverage %: **[7]**

Matched peptides No.: **[3]**

Calculated Mr: **51810** Calculated *p*I: **5.37**

Annotated PFF **spectra:**

Ions score is -10*Log(P), where P is the probability that the observed match is a random event.
Individual ions scores > 35 indicate identity or extensive homology (p<0.05).
Protein scores are derived from ions scores as a non-probabilistic basis for ranking protein hits.

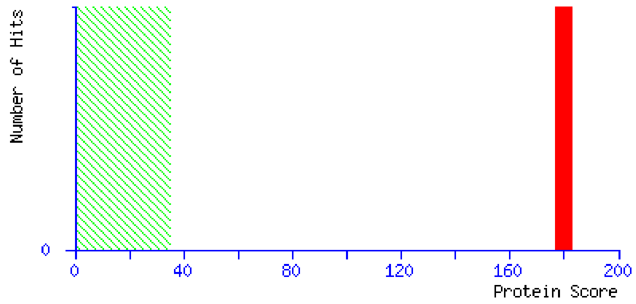

Matched peptide sequences: shown in Bold Red

```
  1 MAQKLEAKGG EMGDVWDDGV YENVRKVYVG QAQYGIAFVK FEYVNGSQVV
 51 VGDEHGKKTE LGVEEFEIDA DDYIVYVEGY REKVNDMTSE MITFLSIKTF
101 KGKTSHPIEK RPGVKFVLHG GKIIGFHGRS TDVLHSLGAY VSLSSTTKLL
151 GKWIKVEQKG EGPGLRCSHG IAQVGNKIYS FGGEFTPNQP IDKHLYVFDL
201 ETRTWSISPA TGDVPHLSCL GVRMVSVGST LYVFGGRDAS RQYNGFYSFD
251 TTTNEWKLLT PVEEGPTPRS FHSMAADEEN VYVFGGVSAT ARLNTLDSYN
301 IVDKKWFHCS TPGDSLTARG GAGLEVVQGK VWVVYGFNGC EVDDVHYYDP
351 VQDKWTQVET FGVRPSERSV FASAALGKHI VIFGGEIAMD PLAHVGPGQL
401 TDGTFALDTE TLQWERLDKF GGEEETPSSR GWTASTTATI GGKKGLVMHG
451 GKAPTNDRFD DLFFYGIDSA
```

Spot No.: **MR2**

NCBI accession No.: **OAP09497.1**

Plant species: ***Arabidopsis thaliana***

Protein name：**JAL22（Jacalin-related lectin 22）**

Peptide sequences: R.KLALCGGEGGQEWDDDVYEGVR.K; R.KVYVGQDLNR.I; K.VYVGQDLNR.I; R.VWDDGSYDGIK.T; R.ITYLEVEYEKDGEAK.T; K.GDTPSEFVLGYPDEYIK.S; R.TSFFGYNVGK.K; K.LPAIGGNEGVTWDDGVYDGVR.K; K.ILVGQGNDGVSFVK.F

PFF Mascot score: **[624]** Sequence coverage %: **[26]**

Matched peptides No.: **[9]**

Calculated Mr: **50604** Calculated *p*I: **5.15**

Annotated PFF **spectra:**

Ions score is -10*Log(P), where P is the probability that the observed match is a random event.
Individual ions scores > 35 indicate identity or extensive homology (p<0.05).
Protein scores are derived from ions scores as a non-probabilistic basis for ranking protein hits.

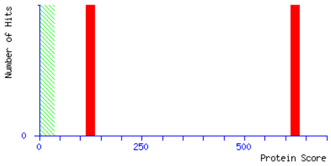

Matched peptide sequences: shown in Bold Red

```
  1 MAKMYRKLAL CGGEGGQEWD DDVYEGVRKV YVGQDLNRIT YIKFEYVQED
 51 GEVVTTEYGT TNQHPKEFVI QYPDEHIIAV EGSYHQVALI ATEVITSLIF
101 KTSKGRKSPL FGPNLLGITT GTKFVFEDEG KKIVGFHGRA GDAVDALGVY
151 FVLDTTPFPL YKLDAQGGTD GRVWDDGSYD GIKTLRIDQD NSRITYLEVE
201 YEKDGEAKTF NHGGKGDTPS EFVLGYPDEY IKSVEATYQK PNIFSNTAIT
251 SLKFLTSKGR TSFFGYNVGK KFVLEQKGHR LVGFHGKEDA AIDALGAYFG
301 PVPTPTPLIP SKKLPAIGGN EGVTWDDGVY DGVRKILVGQ GNDGVSFVKF
351 EYSKGKDLVP GDDHGKKTLL GAEEFVLEDG EYLMNIDGYY DKIFGVEEPI
401 IVCLQFKTNK RESMPFGMDS GKKFSLGEEG HKIVGFHGQA SDVVHSIGVT
451 IVPITTTE
```

Spot No.: **MR3**

NCBI accession No.: **OAP06341.1**

Plant species: ***Arabidopsis thaliana***

Protein name：**JR 1（JA-responsive protein 1）**

Peptide sequences: R.GGEEWDDGGAYENVK.K; K.VYVGQGDSGVVYVK.F; K.TSQPFGLTSGEEAELGGGK.I; R.GGDVWDDGGAYDNVK.K

PFF Mascot score: **[426]**   Sequence coverage %: **[13]**

Matched peptides No.: **[4]**

Calculated Mr: **48548**   Calculated *p*I: **5.17**

Annotated PFF spectra:

Ions score is -10*Log(P), where P is the probability that the observed match is a random event.
Individual ions scores > 35 indicate identity or extensive homology (p<0.05).
Protein scores are derived from ions scores as a non-probabilistic basis for ranking protein hits.

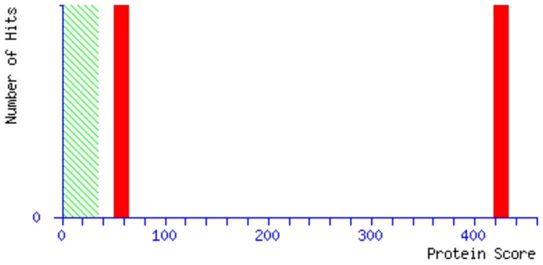

Matched peptide sequences: shown in Bold Red

```
  1 MAKKLEAQGG RGGEEWDDGG AYENVKKVYV GQGDSGVVYV KFDYEKDGKI
 51 VSHEHGKQTL LGTEEFVVDP EDYITSVKIY YEKLFGSPIE IVTALIFKTF
101 KGKTSQPFGL TSGEEAELGG GKIVGFHGSS SDLIHSVGVY IIPSTTPLTP
151 PVSGGLTKLE AQGGRGGDVW DDGGAYDNVK KVYVGQGDSG VVYVKFDYEK
201 DGKIVSHEHG KQTLLGTEEF EIDPEDYITY VKVYYEKLFG SPIEIVTALI
251 FKTFKGKTSQ PFGLTSGEEA ELGGGKIVGF HGTSSDLIHS LGAYIIPSST
301 PLTPSSNTIP AQGGDGGVAW DDGVHDSVKK IYVGQGDSCV TYFKADYEKA
351 SKPVLGSDHG KKTLLGAEEF VLGPDEYVTA VSGYYDKIFS VDAPAIVSLK
401 FKTNKRTSIP YGLEGGTEFV LEKKDHKIVG FYGQAGEYLY KLGVNVAPIA
451 K
```

Spot No.: **MR4**

NCBI accession No.: **OAP06341.1**

Plant species: ***Arabidopsis thaliana***

Protein name：**JR 1（JA-responsive protein 1）**

Peptide sequences: R.GGEEWDDGGAYENVK.K; K.VYVGQGDSGVVYVK.F; K.FDYEKDGK.I; K.TSQPFGLTSGEEAELGGGK.I; R.GGDVWDDGGAYDNVK.K; K.ASKPVLGSDHGK.K; R.TSIPYGLEGGTEFVLEK.K

PFF Mascot score: **[561]** Sequence coverage %: **[22]**

Matched peptides No.: **[7]**

Calculated Mr: **48548** Calculated *p*I: **5.17**

Annotated PFF **spectra:**

Ions score is -10*Log(P), where P is the probability that the observed match is a random event.
Individual ions scores > 35 indicate identity or extensive homology (p<0.05).
Protein scores are derived from ions scores as a non-probabilistic basis for ranking protein hits.

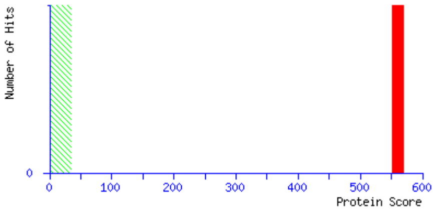

Matched peptide sequences: shown in Bold Red

```
  1 MAKKLEAQGG RGGEEWDDGG AYENVKKVYV GQGDSGVVYV KFDYEKDGKI
 51 VSHEHGKQTL LGTEEFVVDP EDYITSVKIY YEKLFGSPIE IVTALIFKTF
101 KGKTSQPFGL TSGEEAELGG GKIVGFHGSS SDLIHSVGVY IIPSTTPLTP
151 PVSGGLTKLE AQGGRGGDVW DDGGAYDNVK KVYVGQGDSG VVYVKFDYEK
201 DGKIVSHEHG KQTLLGTEEF EIDPEDYITY VKVYYEKLFG SPIEIVTALI
251 FKTFKGKTSQ PFGLTSGEEA ELGGGKIVGF HGTSSDLIHS LGAYIIPSST
301 PLTPSSNTIP AQGGDGGVAW DDGVHDSVKK IYVGQGDSCV TYFKADYEKA
351 SKPVLGSDHG KKTLLGAEEF VLGPDEYVTA VSGYYDKIFS VDAPAIVSLK
401 FKTNKRTSIP YGLEGGTEFV LEKKDHKIVG FYGQAGEYLY KLGVNVAPIA
451 K
```

Spot No.: **MR5**

NCBI accession No.: **BAE98486.1**

Plant species: ***Arabidopsis thaliana***

Protein name: **Glutamine synthetase like protein**

Peptide sequences: R.TLPGPVTDPSKLPK.W; R.HKEHISAYGEGNER.R; K.EHISAYGEGNER.R

PFF Mascot score: **[229]** Sequence coverage %: **[7]**

Matched peptides No.: **[3]**

Calculated Mr: **39323** Calculated *p*I: **5.14**

Annotated PFF **spectra:**

Ions score is -10*Log(P), where P is the probability that the observed match is a random event.
Individual ions scores > 35 indicate identity or extensive homology (p<0.05).
Protein scores are derived from ions scores as a non-probabilistic basis for ranking protein hits.

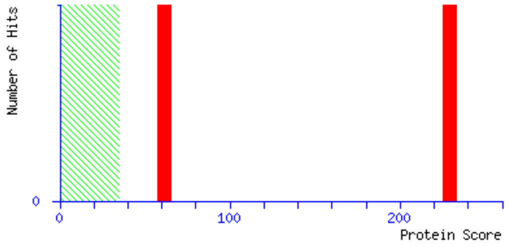

Matched peptide sequences: shown in Bold Red

```
  1 MSLLADLVNL DISDNSEKII AEYIWVGGSG MDMRSKARTL PGPVTDPSKL
 51 PKWNYDGSST GQAPGQDSEV ILYPQAIFKD PFRRGNNILV MCDAYTPAGE
101 PIPTNKRHAA AEIFANPDVI AEVPWYGIEQ EYTLLQKDVN WPLGWPIGGF
151 PGPQGPYYCS IGADKSFGRD IVDAHYKASL YAGINISGIN GEVMPGQWEF
201 QVGPSVGISA ADEIWIPRYI LERITEIAGV VVSFDPKPIP GDWNGAGAHT
251 NYSTKSMREE GGYEIIKKAI EKLGLRHKEH ISAYGEGNER RLTGHHETAD
301 INTFLWGVAN RGASIRVGRD TEKEGKGYFE DRRPASNMDP YVVTSMIAET
351 TLLWNP
```

Spot No.: **MR7**

NCBI accession No.: **NP_188266.1**

Plant species: ***Arabidopsis thaliana***

Protein name：**Mannose-binding lectin superfamily protein**

Peptide sequences: K.VEAGGGAGGASWDDGVHDGVR.K; K.TSPPYGLETQK.K; K.LSAIGGDEGTAWDDGAYDGVK.K; K.VYVGQGQDGISAVK.F; K.QTSAPFGLEAGTAFELK.E

PFF Mascot score: **[425]** Sequence coverage %: **[28]**

Matched peptides No.: **[5]**

Calculated Mr: **32003** Calculated *p*I: **5.06**

Annotated PFF **spectra:**

Ions score is -10*Log(P), where P is the probability that the observed match is a random event.
Individual ions scores > 35 indicate identity or extensive homology (p<0.05).
Protein scores are derived from ions scores as a non-probabilistic basis for ranking protein hits.

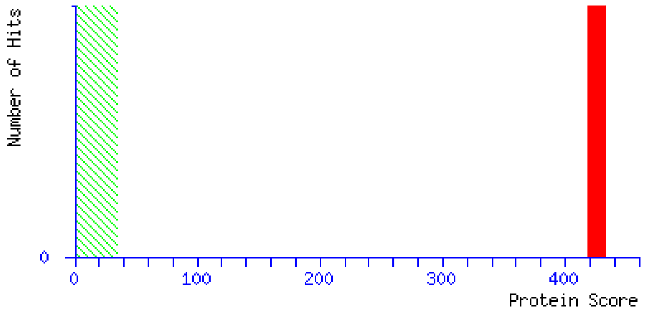

Matched peptide sequences: shown in Bold Red

```
  1 MAQKVEAGGG AGGASWDDGV HDGVRKVHVG QGQDGVSSIN VVYAKDSQDV
 51 EGGEHGKKTL LGFETFEVDA DDYIVAVQVT YDNVFGQDSD IITSITFNTF
101 KGKTSPPYGL ETQKKFVLKD KNGGKLVGFH GRAGEALYAL GAYFATTTTP
151 VTPAKKLSAI GGDEGTAWDD GAYDGVKKVY VGQGQDGISA VKFEYNKGAE
201 NIVGGEHGKP TLLGFEEFEI DYPSEYITAV EGTYDKIFGS DGLIITMLRF
251 KTNKQTSAPF GLEAGTAFEL KEEGHKIVGF HGKASELLHQ FGVHVMPLTN
```

Spot No.: **MR8**

NCBI accession No.: **AAM66134.1**

Plant species: ***Arabidopsis thaliana***

Protein name: **Translationally-controlled tumor protein-like protein**

Peptide sequences: K.VVDIVDTFR.L

PFF Mascot score: **[74]** Sequence coverage %: **[5]**

Matched peptides No.: **[1]**

Calculated Mr: **18925** Calculated *p*I: **4.52**

Annotated PFF **spectra:**

Ions score is -10*Log(P), where P is the probability that the observed match is a random event.
Individual ions scores > 35 indicate identity or extensive homology (p<0.05).
Protein scores are derived from ions scores as a non-probabilistic basis for ranking protein hits.

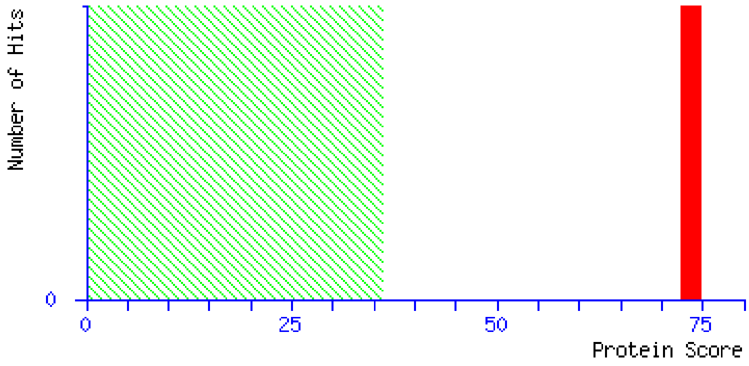

Matched peptide sequences: shown in **Bold Red**

```
  1 MLVYQDLLTG DELLSDSFPY KEIENGILWE VEGKWVTVGA VDVNIGANPS
 51 AEEGGEDEGV DDSAQKVVDI VDTFRLQEQP TYDKKGFIAY IKKYIKLLTP
101 KLSEEDQAVF KKGIEGATKF LLPRLSDFQF FVGEGMHDDS TLVFAYYKEG
151 STNPTFLYFA HGLKEVKC
```

Spot No.: **MR9**

NCBI accession No.: **NP_180766.1**

Plant species: ***Arabidopsis thaliana***

Protein name: **Ribosomal protein L7Ae/L30e/S12e/Gadd45 family protein**

Peptide sequences: R.NAQLCVLAEDCNQPDYVK.L; K.ALCADHSIK.L; K.TLGEWAGLCK.I

PFF Mascot score: **[227]** Sequence coverage %: **[25]**

Matched peptides No.: **[3]**

Calculated Mr: **15661** Calculated *p*I: **5.62**

Annotated PFF spectra:

Ions score is -10*Log(P), where P is the probability that the observed match is a random event.
Individual ions scores > 35 indicate identity or extensive homology (p<0.05).
Protein scores are derived from ions scores as a non-probabilistic basis for ranking protein hits.

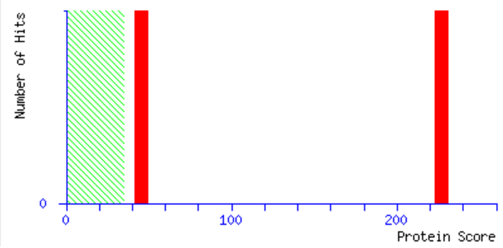

Matched peptide sequences: shown in Bold Red

```
  1 MSGDEAVAAP VVPPVAEAAV IPEDMDVSTA LELTVRKSRA YGGVVRGLHE
 51 SAKLIEKRNA QLCVLAEDCN QPDYVKLVKA LCADHSIKLL TVPSAKTLGE
101 WAGLCKIDSE GNARKVVGCS CLVIKDFGEE TTALNIVKKH LDSN
```

Spot No.: **MR10**

NCBI accession No.: **AAM66134.1**

Plant species: ***Arabidopsis thaliana***

Protein name：**Translationally controlled tumor protein-like protein**

Peptide sequences: **K.VVDIVDTFR.L**

PFF Mascot score: **[74]** Sequence coverage %: **[5]**

Matched peptides No.: **[1]**

Calculated Mr: **18925** Calculated *p*I: **4.52**

Annotated PFF spectra:

Ions score is -10*Log(P), where P is the probability that the observed match is a random event.
Individual ions scores > 35 indicate identity or extensive homology (p<0.05).
Protein scores are derived from ions scores as a non-probabilistic basis for ranking protein hits.

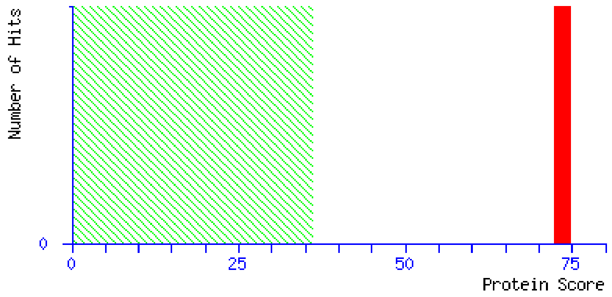

Matched peptide sequences: shown in Bold Red

```
  1 MLVYQDLLTG DELLSDSFPY KEIENGILWE VEGKWVTVGA VDVNIGANPS
 51 AEEGGEDEGV DDSAQKVVDI VDTFRLQEQP TYDKKGFIAY IKKYIKLLTP
101 KLSEEDQAVF KKGIEGATKF LLPRLSDFQF FVGEGMHDDS TLVFAYYKEG
151 STNPTFLYFA HGLKEVKC
```

Spot No.: **MR12**

NCBI accession No.: **NP_565265.1**

Plant species: ***Arabidopsis thaliana***

Protein name: **MLP-like protein 329**

Peptide sequences: K.EIDDENMVITFR.G + Oxidation (M)

PFF Mascot score: **[66]** Sequence coverage %: **[7]**

Matched peptides No.: **[1]**

Calculated Mr: **17705** Calculated *p*I: **5.30**

Annotated PFF **spectra:**

Ions score is -10*Log(P), where P is the probability that the observed match is a random event.
Individual ions scores > 35 indicate identity or extensive homology (p<0.05).
Protein scores are derived from ions scores as a non-probabilistic basis for ranking protein hits.

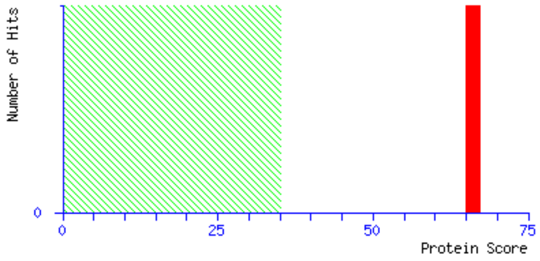

Matched peptide sequences: shown in Bold Red

```
  1 MATSGTYVTE VPLKGSADKH YKRWRDENHL FPDAIGHHIQ GVTVHDGEWD
 51 SHEAIKIWNY TCDGKPEVFK ERKEIDDENM VITFRGLEGH VMEQLKVYDL
101 IYQFSQKSPD DIVCKITMIW EKRTDDSPEP SNYMKFLKSV VADMDEHVLK
151 A
```

Spot No.: **MR13**

NCBI accession No.: **NP_194098.1**

Plant species: ***Arabidopsis thaliana***

Protein name: **Polyketide cyclase/dehydrase and lipid transport superfamily protein**

Peptide sequences: R.SWDYTYDGK.K; R.SWDYTYDGKK.E; R.GLDGHVMEHLK.V; K.ITMIWEKR.N; K.RNDDFPEPSGYMK.F; R.NDDFPEPSGYMK.F; K.QMVVDIEGHVNKA.-

PFF Mascot score: **[414]**   Sequence coverage %: **[35]**

Matched peptides No.: **[7]**

Calculated Mr: **17621**   Calculated *p*I: **5.91**

Annotated PFF **spectra:**

Ions score is -10*Log(P), where P is the probability that the observed match is a random event.
Individual ions scores > 35 indicate identity or extensive homology (p<0.05).
Protein scores are derived from ions scores as a non-probabilistic basis for ranking protein hits.

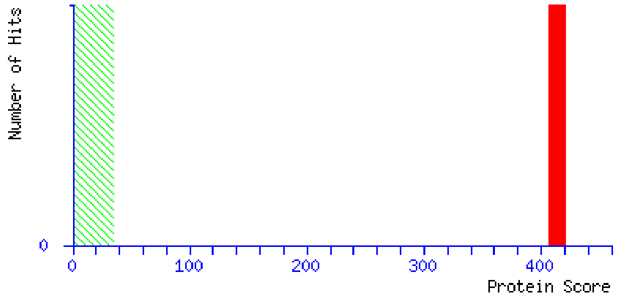

Matched peptide sequences: shown in **Bold Red**

1 MATSGTYVTE VPLKGSAEKH YKSWKSENHV FADAIGHHIQ NVVVHEGEHD
51 SHGSIR**SWDY** **TYDGKK**EMFK EKREIDDENK TLTKR**GLDGH** **VMEHLK**VFDI
101 IYEFIPKSED SCVCK**ITMIW** **EKRNDDFPEP** **SGYMK**FVK**QM** **VVDIEGHVNK**
151 **A**

Spot No.: **MR14**

NCBI accession No.: **NP_565264.1**

Plant species: ***Arabidopsis thaliana***

Protein name： **MLP-like protein 328**

Peptide sequences: K.IWNYTCDGKPEVFK.E;

R.REIDDENMAVTFR.G; R.EIDDENMAVTFR.G;

R.EIDDENMAVTFR.G + Oxidation(M); R.GLEGHVMEQLK.V;

R.GLEGHVMEQLK.V + Oxidation (M)

PFF Mascot score: **[369]**　　　Sequence coverage %: **[25]**

Matched peptides No.: **[6]**

Calculated Mr: **17616**　　　　　Calculated *p*I: **5.42**

Annotated PFF **spectra:**

Ions score is -10*Log(P), where P is the probability that the observed match is a random event.
Individual ions scores > 35 indicate identity or extensive homology (p<0.05).
Protein scores are derived from ions scores as a non-probabilistic basis for ranking protein hits.

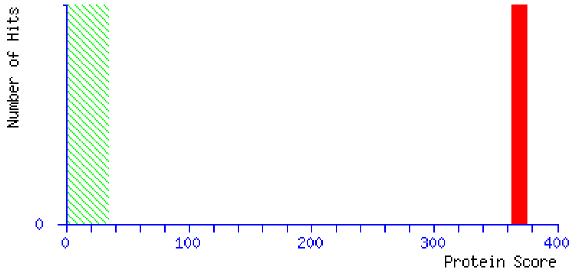

Matched peptide sequences: shown in Bold Red

```
  1 MATSGTYVTE VPLKGSAEKH YKRWRSENHL FPDAIGHHIQ GVTIHDGEWD
 51 SHGAIKIWNY TCDGKPEVFK ERREIDDENM AVTFRGLEGH VMEQLKVYDV
101 IFQFIQKSPD DIICKITMIW EKQNDDMPEP SNYMKFVKSL AADMDDHVLK
151 A
```
